# An ab initio foundation model of wavefunctions that accurately describes chemical bond breaking

**DOI:** 10.1038/s41467-026-76604-2

**Published:** 2026-08-21

**Authors:** Adam Foster, Zeno Schätzle, P. Bernát Szabó, Lixue Cheng, Jonas Köhler, Gino Cassella, Nicholas Gao, Jiawei Li, Frank Noé, Jan Hermann

**Affiliations:** 1 Microsoft Research AI for Science, Cambridge, UK; 2 Freie Universität, Berlin, Germany; 3 Present Address: The Hong Kong University of Science and Technology, Hong Kong, China; 4 Present Address: CuspAI, Cambridge, UK; 5 Present Address: Imperial College London, London, UK; 6 Present Address: Tsinghua University, Beijing, China

**Keywords:** Quantum chemistry, Computational chemistry, Computational science

## Abstract

Reliable description of bond breaking remains a major challenge for quantum chemistry due to the multireference character of the electronic structure in dissociating species. Multireference methods in particular suffer from large computational cost, which under the normal paradigm has to be paid anew for each system at a full price, ignoring commonalities in electronic structure across molecules. Quantum Monte Carlo with deep neural networks uniquely offers to exploit such commonalities by pretraining transferable wavefunction models, but all such attempts were so far limited in scope. Here, we bring this paradigm to fruition with Orbformer, a transferable wavefunction model pretrained on 22,000 equilibrium and dissociating structures that can be fine-tuned on unseen molecules reaching an accuracy–cost ratio rivalling classical multireference methods. On established benchmarks as well as more challenging bond dissociations and Diels–Alder reactions, Orbformer is the only method that consistently converges to chemical accuracy (1 kcal/mol). This work turns the idea of amortizing the cost of solving the Schrödinger equation over many molecules into a practical approach in quantum chemistry.

## Abstract (web summary)

Orbformer is a quantum Monte Carlo foundation model pretrained on thousands of molecular structures. It can be fine-tuned to yield accurate wavefunctions, including for multireference phenomena, with dramatically reduced cost per system.

## Introduction

A major impediment to predicting and optimising the properties of molecules and materials in silico is the computational cost of accurate electronic structure calculations. Ab initio methods that explicitly represent the many-body electronic wavefunction^1^ are the most accurate, but also the most computationally demanding. Further complicating the picture is the dividing line between weakly and strongly correlated systems. For some systems, e.g., most molecules close to their equilibrium geometry, the electronic wavefunction is weakly correlated, allowing the use of ‘single-reference’ methods such as coupled cluster^2^ to compute the wavefunction to very high accuracy. Yet the weakly correlated systems represent only an island in the space of all chemistry and one whose exact boundary is not precisely known. For strongly correlated systems, there is no de facto standard wavefunction method, and calculations often require expert study and individual tuning for each system. Worse still, many multireference methods face a more unfavourable cost scaling than their single-reference counterparts. A scalable method for multireference calculations would therefore be of immense value, not only for making direct predictions, but also for calibrating faster and more approximate methods to handle strongly correlated phenomena such as many chemical reactions and much of transition-metal chemistry.

Ab initio methods are called such because they do not use any information other than the first-principles electronic Schrödinger equation and a choice of numerical approximations. But they can also be characterised by each single calculation being done from scratch, with no computation shared between calculations on similar molecules, despite chemistry owning much of its success to the recognition of recurring patterns in the electronic structure of molecules. The key insight is that these two qualities can be decoupled, and this can be used to drastically lower the cost of ab initio methods. A method derived exclusively from first principles can still share computation between similar molecules, not only within simultaneous calculations but also over time, effectively storing and reusing recurring computation patterns. One might worry that the results could be then affected by the choice of such molecules, but this can be rendered moot if the sharing affects only the rate at which the method arrives at a solution, not the solution itself. It is this shift in paradigm for ab initio methods that is the topic of this work. This new paradigm is also not entirely without precedent—many traditional methods are iterative in nature and use various initial guesses, but these never go beyond the most rudimentary consideration of electronic structure and are typically built with heuristics rather than from first principles. In contrast, the kind of foundation model considered here is fully ab initio, has the same complexity as any final solution it yields, and even before any computation is done on a new molecule, it already encodes its fully correlated electronic structure at a semi-quantitative level. Throughout this work, we use the term foundation model in the specific sense of a single wavefunction model that is pretrained across a broad range of molecules and then serves as a reusable starting point that is adapted to each new system by fine-tuning, rather than in the looser sense of an all-purpose model that is applied to any task without further training.

How can such foundation models be constructed? This challenge has been picked up by a new entrant on the quantum chemistry scene, deep quantum Monte Carlo^3^ (deep QMC), which represents the wavefunction with a deep neural network and trains it towards the ground state by the principle of energy minimisation. The earliest work in this field for molecular systems^4,5^ achieved essentially exact solutions on small molecules. Various new network architectures^6–10^ and extensions^11–19^ have since been proposed. Successful applications to strongly correlated molecules have been particularly encouraging^20^. Whilst the theoretical cost scaling of this approach with system size is favourable, its large prefactor has prevented wider adoption for practical calculations. But thanks to its reliance on deep neural networks, deep QMC is ideally suited to profit from the new paradigm of computation sharing and cost amortisation between molecules. Recent work has explored such new ideas by employing parameter sharing to capture common features in the wavefunctions corresponding to different molecules and geometries^11,21–25^, as well as pretraining a wavefunction model on up to 700 molecular structures before fine-tuning^26–28^. However, we argue that a critical threshold of scale in this endeavour has not yet been crossed. Achieving practical generalisation needs scale in the training throughput, size and diversity of the molecular dataset covered and an architecture that can properly make use of this training signal. With a view to establishing deep QMC as a practical method, one must also take great care when comparing with existing quantum chemistry methods. We believe that the cost–accuracy trade-off must be assessed front and foremost, and that deep QMC has the greatest potential to bring value to quantum chemistry in the area of multireference problems that are challenging for established methods.

In this work, we introduce Orbformer, a neural network wavefunction ansatz. Orbformer operates across molecules of different sizes, compositions, and geometries, but can attain or surpass the same energy accuracy as state-of-the-art single-point ansätze^7^. Orbformer is designed to learn composable features that generalise over different molecules. Modifications of the sampling and variational training procedures allow pretraining a model across 22,000 molecular structures with high geometric and compositional diversity. Orbformer achieves excellent agreement with experimental results on transition states of a Diels–Alder reaction. Compared to classical quantum chemistry methods including DFT, NEVPT2, MRCI, and MRCC on five bond dissociation curves, Orbformer demonstrates a favourable trade-off between cost and accuracy, has a built-in indicator of uncertainty in the form of the variance of the local energies, and can be systematically improved with more fine-tuning, whereas converging classical methods is not always straightforward. Compared to earlier deep QMC approaches, our innovations reduce the cost to reach chemical accuracy by about two orders of magnitude with respect to best single-point calculations, and result in Orbformer being more accurate and benefiting more from pretraining with respect to earlier chemically transferable ansätze. The model learns recognisable features of electronic structure directly from the variational principle, such as the existence of exactly two localised ‘core’ orbitals per carbon atom, without these being hardcoded into the model architecture. Orbformer, though by no means the end of the story, marks significant progress towards a model that generalises the electronic structure of molecules. Given its favourable cost–accuracy trade-off for multireference problems, Orbformer is a useful tool for quantum chemistry applications today.

## Results

### Orbformer: a foundation model for quantum chemistry

We introduce Orbformer, a chemically transferable wavefunction ansatz that simultaneously approximates solutions to the electronic Schrödinger equation for a wide range of molecules. The model accepts as inputs a molecular configuration, **M**, and the spatial-spin co-ordinates of electrons, **x**. Treating **M** as an input to the wavefunction network, rather than a parameter describing an optimisation problem, is at the heart of our approach to sharing computation between similar molecules, and is to the best of our knowledge, an option unique to deep QMC^22,27^. Orbformer is constructedfrom a Jastrow factor and a sum of generalised Slater determinants

1 $$\Psi ({{\bf{x}}}| {{\bf{M}}})={e}\,^{J({{\bf{x}}})}\sum_{d}\det \left[{A}^{d}({{\bf{x}}}| {{\bf{M}}})\right]$$

ensuring that, for every **M**, the model as a function of **x** is a valid antisymmetric wavefunction.

**Fig. 1:**
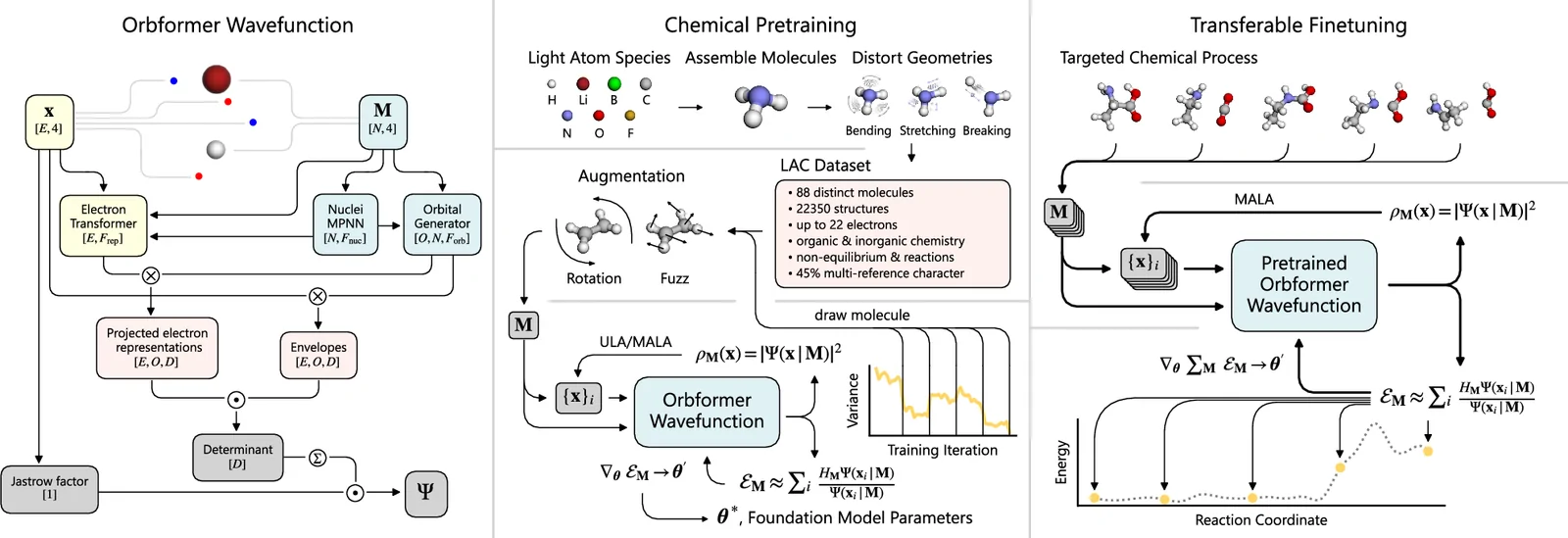
Orbformer pretraining and fine-tuning. The Orbformer wavefunction takes nuclear positions and charges, **M**, and electron positions and spins, **x**, as inputs to generate generalised Slater matrices. Linear combinations of these generalised Slater determinants, combined with an electron–electron Jastrow factor, give a chemically transferable wave function, *Ψ*, that can generalise across systems of varying size. Orbformer is pretrained on a diverse dataset of small molecules and fragments, yielding a foundation model for non-equilibrium quantum chemistry. To achieve accuracies of 1 kcal/mol, the pretrained Orbformer model is then jointly fine-tuned on a process-specific dataset. Finally, the energy and other observables are evaluated for each of the involved molecular geometries.

The major components of the Orbformer ansatz are: an Electron Transformer, which processes electron features through a sequence of attention layers; and the Orbital Generator, which dynamically creates generalised orbitals for **M**, each consisting of an envelope and a projection of the electron representations (Fig. 1 left). A guiding principle in the design of the ansatz is locality—the interactions between any two particles are carefully crafted to decay with increasing distance, and the Orbital Generator is constrained to produce localised orbitals. We show that this facilitates the re-use of orbitals when similar chemical environments arise in different molecules. Full details of our architecture are given in “Orbformer wavefunction architecture” section.

We train the Orbformer wavefunction to minimise the expectation value of the Hamiltonian operator

2 $${{\mathbb{E}}}_{{{\bf{M}}} \sim {p}_{{{\rm{train}}}}({{\bf{M}}}),{{\bf{x}}} \sim | \Psi ({{\bf{x}}}| {{\bf{M}}}){| }^{2}}\left[\frac{{\hat{H}}_{{{\bf{M}}}}\Psi ({{\bf{x}}}| {{\bf{M}}})}{\Psi ({{\bf{x}}}| {{\bf{M}}})}\right],$$

where the expectation is taken over molecular configurations sampled from a training distribution and electron co-ordinates sampled from the unnormalized distribution defined by the current model. This self-generative scheme does not require any external input data apart from the choice of molecular training distribution. Our set-up generalises standard variational Monte Carlo (VMC) by the addition of an outer expectation over molecular configurations.

Orbformer’s amortisation of computational cost arises from the combination of chemical pretraining and transferable fine-tuning. During pretraining, we present the model with an extremely diverse set of molecules and train variationally to encode the electronic structure of a swathe of chemical space to a semi-quantitative level. We specifically created a pretraining dataset of 22,350 molecular configurations with up to 24 electrons composed of H, Li, B, C, N, O, F in various geometric configurations, as illustrated in Fig. 1 (centre). This Light Atom Curriculum (LAC) dataset is specifically designed to involve multireference out-of-equilibrium geometries. To maximise speed and improve stability, we pretrain in three phases of increasing size/complexity, as detailed in Table 1. We introduce innovations in the deep QMC training protocol to aid efficient, large-scale training, which we detail in Methods. The same set of model parameters can be transferred from smaller to larger systems, and there is no baked-in maximum number of particles. In this paper, our largest pretraining system had 24 electrons, whereas the largest fine-tuning system had 106 electrons.

**Table 1:** Phases of Orbformer chemical pretraining using the Light Organic Curriculum (LAC) dataset

Phase	Data	Steps	M batch size	Electrons per M	A100 hours (est.)
1a	LAC ≤ 10 electrons, bend & stretch geometries	200 k	8	1024	320
1b	LAC ≤ 10 electrons, all geometries	200 k	8	1024	320
2	All LAC	400 k	16	1024	4000

To attain high-accuracy wavefunctions, we start from the pretrained model and fine-tune to convergence. We do not resort to separate fine-tuning for every structure; instead, we group target structures together and fine-tune them using a single set of model parameters (Fig. 1 right). For example, we routinely fine-tune all geometries with the same set of nuclei together. The amortisation from this approach can lead to a cost reduction proportional to the number of geometries, over and above the benefits of pretraining.

### Diels–Alder reaction pathways

We compare Orbformer predictions to experimental data on a practically relevant chemical problem. The task of characterising the mechanism of the iconic Diels–Alder reaction^29^ (cycloaddition of ethene and butadiene) is chosen for this purpose, as its exact mechanism was subject to debate until recently^30,31^, and its accurate description requires careful treatment of both static and dynamic correlation^31,32^.

**Fig. 2:**
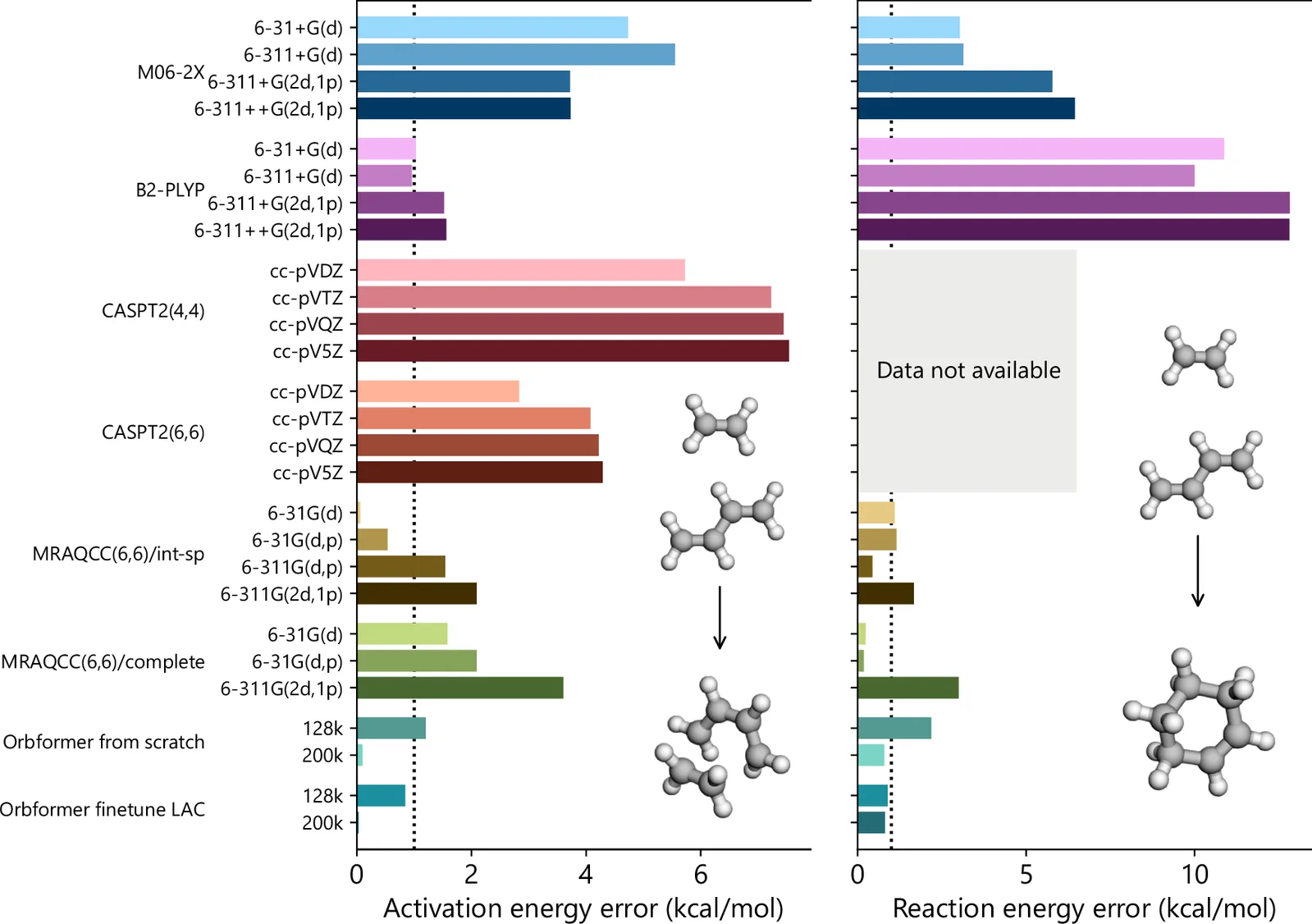
Accuracy for activation and reaction energies of the iconic Diels–Alder reaction. Activation energies use the transition state of the concerted pathway. The experimental values are computed from the forward and backward activation enthalpies measured by Rowley^45^ and Uchiyama^33^, respectively, applying zero-point and thermal corrections computed at the B3LYP/6-31G** level^32^. The M06-2X and B2-PLYP density functional results are taken from the work of Cui et al. ^30^, complete active space second order perturbation theory values are obtained from Scarborough^31^ but are not published for the reaction energy, while multireference coupled cluster results were computed by Lischka and coworkers^32^, from where the molecular geometries utilised for the Orbformer calculations are also taken. The colour gradients represent results obtained with increasingly large single-particle basis sets or QMC steps, as applicable. The multireference coupled cluster results employed an active space composed of six orbitals and six electrons, with two sets of configuration state functions: a complete one, and a smaller one built by applying the interacting space restriction from reference configurations with the same symmetry as the electronic state^32^.

Two major pathways have been identified along which the reaction might proceed: a concerted one where the two bonds between the reactants form synchronously, and a two-step mechanism involving biradical intermediates, where the bonds form sequentially^30^. Using the concerted transition state, Orbformer converges to almost perfect agreement with the experimental activation energy (Fig. 2). Chemical pretraining improves the convergence rate, despite the transition state being well outside of the pretraining distribution at 46 electrons and involving the simultaneous breaking of two bonds. Of the traditional wavefunction based methods, only multireference coupled cluster achieves results agreeing with experiment, but only if a small basis set or an artificially restricted set of configuration state functions are employed. For the two-step pathway, Orbformer predicts an activation energy that is about 9.9 kcal/mol higher than the experimentally measured activation energy (see Supplementary Note 11.1 for detailed results), clearly indicating that this is not the favoured mechanism. Our predicted energy difference between the two pathways is in good agreement with the current theoretical and experimental consensus^30,33^. For the overall reaction energy, we also found that Orbformer reliably converges to within 1 kcal/mol of the experimental value. For this calculation, where strong correlation is expected to be less important, classical methods fare better, but still exhibit a troubling sensitivity to the choice of basis set.

These results show that Orbformer is a reliable and highly accurate method for dealing with transition state calculations in realistic settings. For additional benchmarking against experiments, we compared Orbformer predictions against an experimental reference for N_2_ dissociation (Supplementary Note 11.2) and against experimental atomisation energies of small molecules (Supplementary Note 11.6). In both cases, Orbformer matches extremely well with experimental references.

### Cost–accuracy trade-offs of high-accuracy multireference calculations on bond breaking

**Fig. 3:**
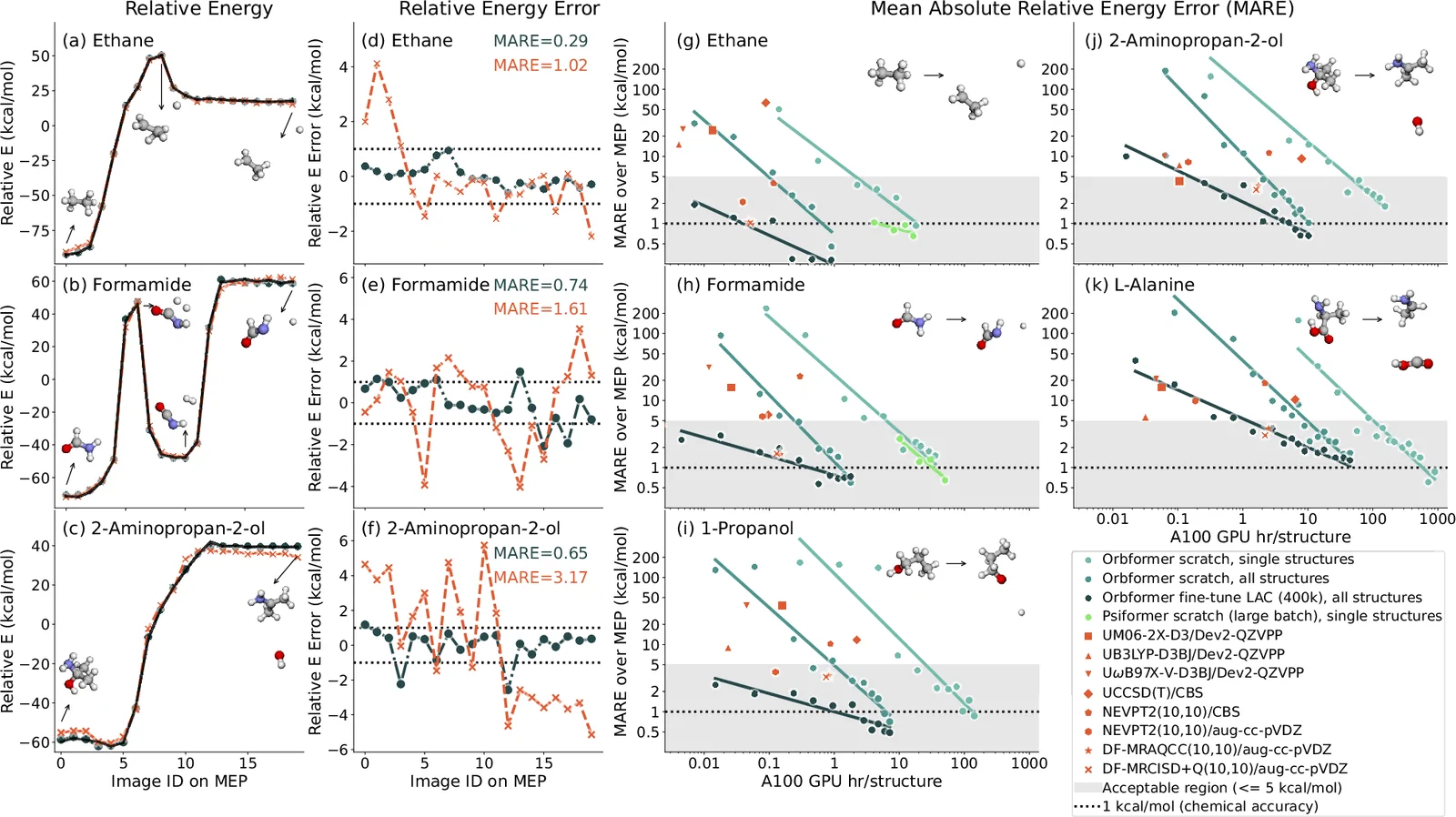
Accuracy on minimum energy paths (MEPs) of five molecular dissociations. The colour and marker for each method are the same across all panels (**a–k**) and are defined in the shared key. Left column: Reaction profile plotted as mean-centred relative energy for (**a**) Ethane (**b**) Formamide and (**c**) 2-Aminopropan-2-ol. The solid black line shows the reference relative energies; the other two curves show the longest fine-tuned Orbformer prediction (Orbformer fine-tune LAC (400 k)) and the DF-MRCISD+Q(10,10)/aug-cc-pVDZ result. Middle column: Relative energy error of each structure in the corresponding MEPs for (**d**) Ethane (**e**) Formamideand (**f**) 2-Aminopropan-2-ol. Panels (**a**–**f**) together show how the mean absolute error in relative energy is calculated across the reaction curve. Right column: Mean absolute error in relative energy for a wide range of computational methods for (**g**) Ethane (**h**) Formamide (**i**) 1-Propanol (**j**) 2-Aminopropan-2-ol, and (**k**) L-Alanine. The circles in blue/green colours with best-fit lines represent results from different deep QMC variants. Traditional electronic structure theories are shown in orange with different marker symbols indicating the precise method used. The dashed line indicates chemical accuracy of 1 kcal/mol. The shaded area represents an acceptable accuracy region of 5 kcal/mol. For a method that does not run on a GPU, we convert the computational cost using 1 GPU hour = 68.6 CPU core hours, computed from the power ratings of an A100 GPU and an AMD EPYC 7763 CPU (cf. Supplementary Note 9.2). Where a method uses multiple CPU cores or multiple GPU devices, this is accounted for in the cost.

For a more detailed examination of the cost–accuracy trade-off of Orbformer, we investigate relative energy errors at 20 points along a bond dissociation pathway in five different molecules with up to 48 electrons. Fig. 3 (left column) illustrates some of the chosen dissociations, which are intended to give good coverage of the strongly correlated intermediate region. For such detailed coverage of the potential energy surface, experimental references are not available. Reference energies for this experiment were therefore obtained from independent, single-point deep QMC calculations run at very high cost to convergence, using a deliberately different and more expensive setup than the Orbformer calculations under test. We validated this protocol against an established single-reference benchmark by comparing the difference in energy between the two dissociation endpoints (the subset of structures where single-reference methods are reliable) to literature (RO)CBS-QB3^34^ dissociation energies. We found that the dissociation energies from our reference QMC protocol matched (RO)CBS-QB3 with a tolerance of 1 kcal/mol for all five dissociation curves (cf. Supplementary Note 9.2, Supplementary Tables 6 and 7 for further details). Throughout this section, accuracy is judged with respect to these reference energies.

For each candidate method, we compute the relative energy error to the reference by removing any fixed offset between the energy profiles, and calculating the mean absolute relative error (MARE, defined in Supplementary Note 8.3) across all geometries (Fig. 3 middle column). Comparison is made to the MRCISD, MRAQCC, NEVPT2, and CCSD(T) wavefunction methods, and several DFT functionals, as these all have reasonable software support to treat systems with up to 48 electrons. For the computational cost, we measure the machine hours consumed and use the maximum power ratings of the respective devices as our conversion factor between CPU and GPU hours.

It is apparent in each of Fig. 3g–k that Orbformer fine-tuned from LAC pretraining is either on, or significantly ahead of, the Pareto front formed by the set of orange points, i.e., from cheaper density functional methods and increasingly costly complete active space-based wavefunction approaches. Even more striking is the fact that Orbformer exhibits a strict monotonic convergence with expended computational effort in all systems presented, while achieving systematic convergence with traditional methods is often difficult or impossible, see e.g., Diels–Alder reaction pathways. We therefore believe that Orbformer represents the best available method for the modelling of strongly correlated molecular systems in dissociations of this nature.

The three Orbformer variants shown in Fig. 3g–k isolate the improvements brought about from joint fine-tuning and pretraining. Optimising the ansatz jointly across all geometries of a given minimum energy pathway (MEP) yields an approximately 20 × efficiency gain over independent, single-point optimisation, amounting to almost perfect amortisation of cost over the 20-point dissociation curve. On top of this, starting the joint fine-tuning from the Orbformer model pretrained on the LAC dataset, one obtains additional gains in efficiency. Here, we see that proximity to the pretraining distribution is important, with about a 16 × decrease in cost to reach chemical accuracy on ethane (in the LAC dataset), a 6 × decrease on 1-propanol (somewhat larger than LAC). For L-Alanine (much larger than LAC), the pretrained model reaches a 5 kcal/mol error 8 × faster than its non-pretrained counterpart, but a 1 kcal/mol error only about 10% faster. Thus, pretraining is always beneficial, but decreases in importance with longer fine-tuning, as the network shifts to be more specialised to the fine-tuning task. Nevertheless, the benefits from pretraining on out-of-distribution molecules persist down to chemical accuracy.

Not shown here is training without our engineering improvements, which would be up to 2.6 × slower across all Orbformer variants (see Supplementary Note 11.3). In totality, our approach recovers the results of independent, single-point deep QMC reference calculations at a fraction of the cost and so brings deep QMC to the cost–accuracy Pareto frontier formed by the range of classical methods that we have studied here.

### Comparison with existing deep QMC and the impact of pretraining

We now compare Orbformer to previous attempts at chemical transferability within the deep QMC framework^22,26,27^. For this, we employ the TinyMol dataset^27^, which has been used as a benchmark for medium-scale chemical transferability^22^. This is an easier dataset without strong correlation and features CCSD(T)/CBS reference energies. The TinyMol benchmark comes with its own small pretraining dataset, while the test set is divided into in-distribution (different geometries of pretraining molecules) and out-of-distribution (molecules not present during pretraining). In addition to our main model pretrained from the LAC, we pretrained a separate Orbformer using the TinyMol pretraining set.

**Fig. 4:**
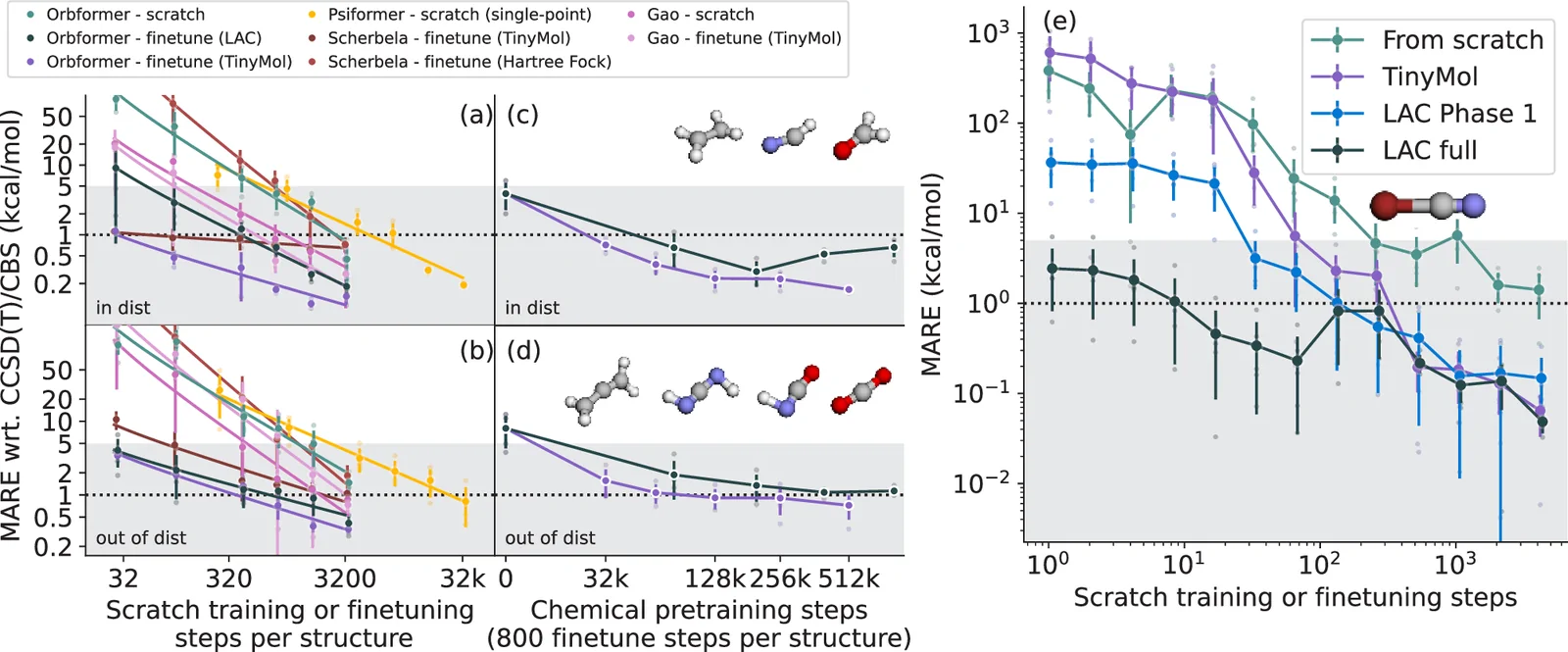
Convergence with respect to pretraining and fine-tuning. Mean absolute error in relative energy on the TinyMol in-distribution (**a**) and out-of-distribution (**b**) test sets. The error is computed with respect to reference energies obtained with CCSD(T) extrapolated to the complete basis set limit^27^. Each data point in panels (**a**–**d**) is averaged over ten geometries of three (in-distribution) or four (out-of-distribution) molecules; the error bars show the standard deviation across those molecules, and the individual per-molecule values are overlaid as faint points. The dotted horizontal line indicates chemical accuracy. Panels (**c**, **d**) compare the two pretraining datasets used for Orbformer and the impact of the length of the pretraining, at 800 fine-tune steps per structure, on the in-distribution and out-of-distribution test sets, respectively. The effect of the pretraining dataset composition on the speed of fine-tuning on a new LiCN test set is shown on panel (**e**), where each point is averaged over five LiCN geometries, the error bars show the standard deviation across those geometries, and the individual per-geometry values are overlaid as faint points. Note that Li is not present in the TinyMol pretraining dataset but is present in the LAC.

The obtained MAREs are compared to the results of Scherbela et al.^26^, Gao et al. ^22^ and a single-point Psiformer ^7^ baseline, on the left panel of Fig. 4. We see clearly that Orbformer, pretrained on either the LAC or the TinyMol pretraining set, is the best performing model for both in- and out-of-distribution test sets. On both test sets, the pretrained Orbformer reaches chemical accuracy at least an order of magnitude faster compared to training from scratch. The single-point Psiformer is accurate but more costly because it does not exploit the joint fine-tuning methodology. Compared to the baseline of ref. ^28^ we see improvement, both in terms of accuracy at each fine-tuning iteration, as well as time to convergence to chemical accuracy. Compared to ref. ^23^, we see that for out-of-distribution test structures, their model is actually harmed by more pretraining, whereas Orbformer still benefits from it.

On the centre panel of Fig. 4, we see in addition that longer fine-tuning of Orbformer is beneficial, although the rate of improvement slows beyond about 100 k steps. On the TinyMol test sets, pretraining using the TinyMol pretraining dataset is clearly better than pretraining using the much larger LAC dataset. We believe that this is because the TinyMol pretraining distribution is much more aligned to the TinyMol test distributions. To confirm this hypothesis, we created an additional small test set of five geometries of LiCN undergoing bond stretching. This molecule is in the pretraining distribution of the LAC, is missing from the Phase 1 LAC, while the TinyMol pretraining dataset doesn’t even contain the Li species. As seen on the right panel of Fig. 4, LAC pretraining is now the most effective, followed by LAC Phase 1. This indicates, as expected, that molecules that are closer to the pretraining distribution can be fine-tuned faster, and is an encouraging sign that scaling up pretraining datasets will result in improved coverage of chemical space.

### Inner workings of the Orbformer model

**Fig. 5:**
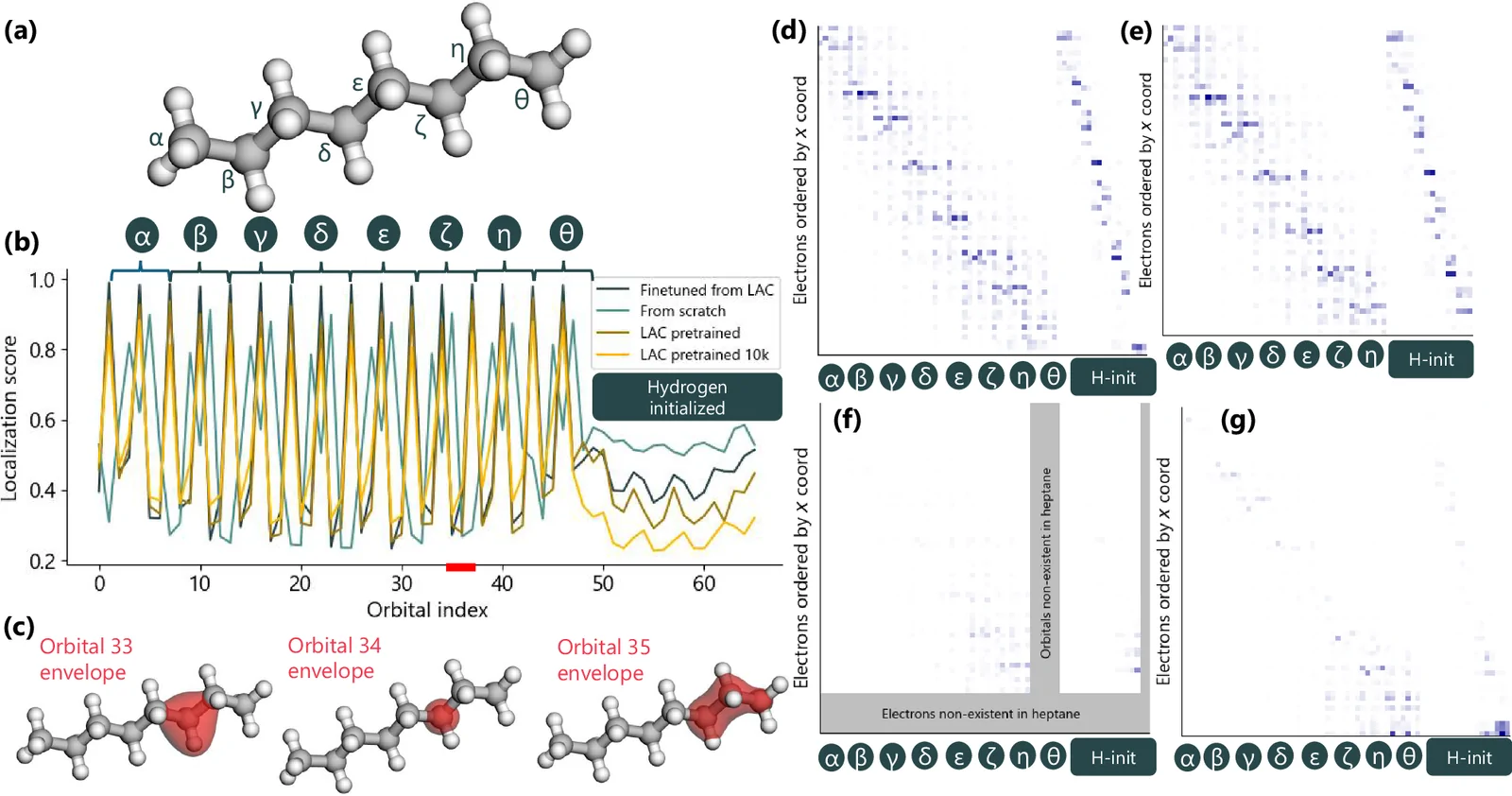
Learning electronic structure patterns from data. Orbformer was fine-tuned on a dataset of alkanes with 6 to 13 carbons inclusive. **a** Octane molecule with carbons labelled. **b** Orbformer orbital envelopes for octane ordered by the nucleus on which they are initialised: first all carbons, then all hydrogens. The localisation score indicates whether a given orbital envelope places all weight on a single nucleus. The LAC pretrained and LAC pretrained 10 k checkpoints were used as is on this molecule with no fine-tuning. **c** Isosurfaces of some of the envelopes shown above, here Orbital 34 has a localisation score close to 1.0. **d** One of the Orbformer Slater matrices for the octane molecule (using the LAC fine-tuned model), where we plot the pointwise absolute value, ∣*A*^*d*^∣, and white denotes 0. The electrons are ordered by their *x* co-ordinate which approximately corresponds to the order of the carbons from *α* to *θ*. Orbitals are ordered exactly as in (**b**). **e** Using the same Orbformer parameters, we now compute the corresponding Slater matrix for the heptane molecule. Electron positions and spins are the same as for octane but with 8 electrons removed based on *x* co-ordinate. **f** The pointwise absolute difference between the Slater matrices for heptane and octane, ∣*A*^*d*^(⋅ ∣oct) − *A*^*d*^(⋅ ∣hept)∣. The orbitals are arranged so that analogous orbitals that are initialised on the same nucleus in both molecules appear in the same place. Grey shading indicates electrons and orbitals which no longer exist in heptane. White colour denotes a difference of 0, and the scaling is the same as in (**d**). **g** The absolute value of the gradient of the Slater matrix presented in (**d**) with respect to the *x*-position of the rightmost electron.

To analyse the properties of electronic structure learned by Orbformer, we fine-tune a single Orbformer model on a dataset of systematically generated alkane chains with 6 to 13 carbon atoms (transferable fine-tuning). Several orbital envelopes—simple single-particle functions that control the spatial extent of Orbformer’s generalised orbitals—are illustrated in Fig. 5c. The first intriguing property we found was that, without direct supervision, Orbformer learns to assign exactly two completely localised orbitals on every carbon atom, as seen in Fig. 5b. It therefore appears that something akin to core electron orbitals are learned by the model for the carbon species, directly from transferable application of the variational principle. We found that this orbital localisation pattern actually emerges after as few as 10k Orbformer pretraining steps. When training from scratch, some localisation does emerge, but not to the same degree as the model fine-tuned from LAC pretraining. This is not surprising, considering that for a single molecule, properties of the determinant mean that there is no particular need for orbital localisation, yet when attempting to generalise across similar molecules, the property appears highly beneficial. One may wonder whether orbital localisation has the potential to harm performance on conjugated systems. In Supplementary Note 11.5 we fine-tuned Orbformer on azulene–naphthalene isomerization and showed that our reaction energy agrees very well with CCSD(T) at the complete basis set limit, indicating that the degree of localisation can be adapted to the electronic structure required and does not practically limit the network’s expressivity.

Returning to our systematic investigation of alkanes, we further investigate the extent to which the orbitals are transferable across molecules with the same local structure. We extract the Slater matrices created by Orbformer for octane in Fig. 5d, but also apply the same network to the smaller heptane molecule, using the octane electron positions after removing enough electrons to maintain electric neutrality, shown in (e). In Fig. 5f, we now compute the pointwise difference between the octane Slater matrix in (d) and the heptane one in (e). What we see is a striking confirmation of the composability property of Orbformer. Namely, for electrons close to the *α*-labelled carbon, which have essentially the same local environment in both octane and heptane, we see that the corresponding rows of the Slater matrix are identical for octane and heptane. Electrons at the other end of the molecule see much larger variation in corresponding orbital values, because their local environments are not the same. This provides firm evidence that orbitals in Orbformer are re-used between different molecules. For an additional confirmation, panel (g) shows the gradient of the Slater matrix in (d) with respect to the position of the rightmost electron (nearer the *θ*-labelled carbon). It is clear that the Orbformer wavefunction is primarily local, with the strongest gradient occurring for the orbitals and electrons nearest to the perturbation. However, it is evident that the model does selectively retain a few longer-range interactions. Overall, the requirement of chemical transferability forces the model to develop a theory of electronic structure in a way that is different from models that are optimised anew for every structure.

## Discussion

Orbformer demonstrates the possibility of large-scale amortisation of computational cost in high-accuracy quantum chemistry through pretraining and transferable fine-tuning of a neural network wavefunction model. We saw that Orbformer is competitive with traditional wavefunction methods, not only because it is eventually more accurate, but because it can achieve a favourable cost–accuracy trade-off. This is only possible because of a drastic reduction in the per-structure computational cost brought about by learning to exploit recurring patterns in the electronic wavefunctions for different molecules and geometries.

For large-scale usage, we envisage Orbformer as a data generation method for a much cheaper model, such as a reactive force field applicable to multireference systems. With deep QMC approaching the cost of traditional methods, it can become a powerful and unique addition to the world of electronic structure.

## Methods

### Orbformer wavefunction architecture

The Orbformer model introduced in Orbformer: a foundation model for quantum chemistry is a chemically transferable ansatz, meaning it accepts the molecular configuration **M** as an explicit input, rather than as a parameter which defines a particular optimisation problem. A trained Orbformer therefore represents the wavefunction for various molecules using a single set of neural network parameters. The architectural changes required to implement a chemically transferable ansatz of this form are substantial when compared to single-molecule methods for molecular ground states with neural network ansätze^4,5^. In particular, a subnetwork that treats the molecular configuration input, which in Orbformer is the Orbital Generator, is now required. The basic form of the Orbformer ansatz combines a scalar Jastrow factor, *J*_***θ***_, with a sum over generalised Slater determinants,

3 $${\Psi }_{{{\boldsymbol{\theta }}}}({{{\bf{x}}}}_{1:E}| {{\bf{M}}})={e}\,^{{J}_{{{\boldsymbol{\theta }}}}({{{\bf{x}}}}_{1:E})}\sum_{d}\det \bigg[{A}^{d}_{{{\boldsymbol{\theta }}}} ({{{\bf{x}}}}_{1:E}|{{\bf{M}}})\bigg],$$

where each ${A}_{{{\boldsymbol{\theta }}}}^{d}$ is an all-electron *E* × *E* matrix. In contrast to traditional Slater determinants built from single-particle orbitals, these generalised Slater matrices contain permutation-equivariant many-body functions: each row depends not only on the position and spin of a single electron but on those of all electrons. The permutation equivariance then guarantees that *Ψ*_***θ***_ is antisymmetric under electron exchange.

The Jastrow factor *J*_***θ***_ enforces the electron–electron cusp conditions^35^ and is given by

4 $${J}_{{{\boldsymbol{\theta }}}}({{{\bf{x}}}}_{1:E})=-\frac{1}{4}\sum _{e < {e}^{{\prime} },{\sigma }_{e}={\sigma }_{{e}^{{\prime} }}}^{E}{\alpha }_{{{\rm{par}}}}{e}^{-\parallel {{{\bf{r}}}}_{e}-{{{\bf{r}}}}_{{e}^{{\prime} }}\parallel /{\alpha }_{{{\rm{par}}}}}-\frac{1}{2}\sum _{e < {e}^{{\prime} },{\sigma }_{e}\ne {\sigma }_{{e}^{{\prime} }}}^{E}{\alpha }_{{{\rm{anti}}}}{e}^{-\parallel {{{\bf{r}}}}_{e}-{{{\bf{r}}}}_{{e}^{{\prime} }}\parallel /{\alpha }_{{{\rm{anti}}}}},$$

where *α*_par_, *α*_anti_ are learnable parameters, electrons are indexed by *e* = 1, …, *E* and *σ*_*e*_ denotes the spin of electron *e*. This exponentially decaying form allows the function *J*_***θ***_ to become additive over well-separated subsystems.

Each generalised Slater matrix is formed as a pointwise product ${A}_{{{\boldsymbol{\theta }}}}^{d}={\Omega }_{{{\boldsymbol{\theta }}}}^{d}\odot {\Phi }_{{{\boldsymbol{\theta }}}}^{d}$ of envelopes, *Ω*, and projected electron representations, *Φ*. The envelopes dictate the spatial extent of each orbital. For each nuclear charge we associate a set of learnable exponents ${\zeta }_{f}^{Z}$ that define radially symmetric primitive envelopes ${P}_{enf}={e}^{-{\zeta }_{f}^{{Z}_{n}}\parallel {{{\bf{r}}}}_{e}-{{{\bf{R}}}}_{n}\parallel }$ on the electron–nuclei distances; these are combined using coefficient matrices ${W}_{donf}^{\,{\rm{env}}\,,\sigma }$ that are an output of the Orbital Generator,

5 $${({\Omega }_{{{\boldsymbol{\theta }}}}^{d})}_{eo}=\sum _{n=1}^{N}\sum _{f=1}^{{F}_{{{\rm{env}}}}}{P}_{enf}{W}_{donf}^{{\rm{env}}\,,{\sigma }_{e}}.$$

Here, *d*,  *o*,  *n* and *f* index determinants, orbitals, nuclei and feature dimensions, respectively. The projected electron representations are obtained from the permutation-equivariant electron representations *T*_*ef*_ generated by the Electron Transformer together with coefficient matrices ${W}_{dof}^{\,{\rm{repr}}\,,\sigma }$ from the Orbital Generator,

6 $${({\Phi }_{{{\boldsymbol{\theta }}}}^{d})}_{eo}=\sum _{f=1}^{{F}_{{{\rm{repr}}}}}{T}_{ef}{W}_{dof}^{{\rm{repr}}\,,{\sigma }_{e}}.$$

The electron representations are generated from an Electron Transformer. Our Electron Transformer network first pools incoming signals from each nucleus to each electron, to create a starting electron representation. These representations are then passed through a number of self-attention layers with an attention bias term that is computed from the electron–electron distances such that the attention logits decay to zero between very distant electrons. Through the attention mechanism, every electron representation depends on the positions and spins of every other electron, and the set of electron representations is equivariant to a permutation of the electrons. The former property means that Orbformer ‘orbitals’ are a strict generalisation of orbitals in classical quantum chemistry (which only depend on a single electron). The latter property ensures that the ansatz overall is antisymmetric under the exchange of two electrons, as required. Orbformer is, to our knowledge, the first model that successfully combines a transformer^36^ architecture with generalisation across molecules.

The Orbital Generator subnetwork allows Orbformer to model the wavefunction for multiple molecules simultaneously. It produces the coefficient matrices ${W}_{donf}^{\,{\rm{env}}\,,\sigma }$ and ${W}_{dof}^{\,{\rm{repr}}\,,\sigma }$ that are used to form *Ω* and *Φ*. The Orbital Generator depends only on the input molecular configuration, **M**, and not on the electron positions and spins, **x**. The Orbital Generator has two main stages: (i) initialisation, and (ii) message passing. In the initialisation stage, orbital representations are instantiated that are completely localised to a single nucleus and depend only on the atom types that are present in the molecule. For example, in the LiH molecule, the network will initialise three distinct orbitals on the Li atom and one orbital on the H atom. Although crude, this scheme ensures that charges are locally balanced at initialisation. In the message passing stage, a sequence of message passing layers updates the orbital representations to respond to the overall geometry of the molecule. This allows the network to see the local structure of the molecule and to respond accordingly, and it allows orbitals to delocalise away from their nucleus of initialisation.

The property of composability is a constraint that we apply to the network architecture to encourage the network to learn generalisable features that can be re-used between different molecules that share similar sub-structure (e.g., a functional group). Composability guarantees size consistency for a single-determinant Orbformer (but not when multiple determinants are used). Consider electrically neutral molecules **M**_1_, **M**_2_ containing *E*_1_, *E*_2_ electrons respectively. In the limit of infinite distance between **M**_1_, **M**_2_, a composable chemically transferable ansatz will have exactly *E*_1_ orbitals localised to **M**_1_ and *E*_2_ orbitals localised to **M**_2_; the value of the orbitals localised to **M**_1_ will be independent of the nucleus and electron positions at **M**_2_ and the same as would be obtained when running the ansatz on **M**_1_ alone, and vice versa; and the Jastrow factor for the combined system will be the sum of the Jastrow factors for the two separate systems. In Supplementary Note 3.1, we show that composability means that the Slater matrices *A*^*d*^ for the combined system of **M**_1_, **M**_2_ can be brought to a block diagonal form by a sequence of column and row swaps.

Full details of the model architecture are given in Supplementary Note 3.3, and a detailed discussion of how Orbformer relates to existing transferable QMC methods can be found in Supplementary Note 3.8.

### Accelerating and stabilising the model

Large-scale pretraining is only possible if the model is fast enough and stable enough to accommodate pretraining over a large, diverse dataset. We found that many of the ansatz changes introduced to satisfy the composability constraint actually led to significant improvements in model stability as well. In particular, ensuring that all particle–particle interactions decay to zero in the large distance limit, and initialising the orbitals to be partially localised appear to have been very beneficial for training stability.

As a consequence of this, we found that we could train Orbformer at TensorFloat-32^37^ precision, a lower (hence faster) precision than the full Float-32 precision used for all previous deep QMC optimisations. We also found that we could train with substantially smaller electron batch sizes than some previous work, for example, we were able to train Orbformer with batch sizes that were at least 4 × smaller than electron batch sizes used for Psiformer^7^. We also found that Orbformer behaved stably with very large distance separations (e.g., testing size consistency), unlike some other existing models.

To further accelerate the model, we adopted the Forward Laplacian framework that was introduced by ref. ^16^ using the implementation in the folx package^38^. Another avenue of acceleration was to switch to using FlashAttention^39^ for the electron–electron attention layers. FlashAttention both accelerates the attention layers and reduces their GPU memory consumption. Unfortunately, there is no existing FlashAttention algorithm for the Laplacian of the attention layers.

Rather than fall back on the default Laplacian, which is slower and more memory intensive, we introduce a FlashAttention-inspired algorithm to compute the Forward Laplacian updates for the electron–electron attention layers. We implemented this using pallas^40^, a kernel language for JAX. This kernel has now been made available in the folx package. The precise details of our Laplacian implementation are given in Supplementary Note 6.2.

### Generating electron configurations for chemically transferable training

In single-molecule deep QMC training, the training data is generated from running Markov Chain Monte Carlo (MCMC) on the model itself and used to converge the ansatz towards the theoretical ground state wave function. For Orbformer pretraining, we sample molecular configurations from our LAC dataset and apply data augmentation. This presents a problem, however, as we must be able to sample electron configurations from the distribution ∣*Ψ*(**x**∣**M**)∣^2^ for a molecule **M** that has not been previously encountered during pretraining. This requires us to be able to burn in MCMC samplers for new molecules much faster than in previous works.

One natural MCMC algorithm for generating electron configuration data is the Metropolis-Adjusted Langevin Algorithm (MALA)^41^. This combines a Langevin proposal of step-size *τ*

7 $${{{\bf{x}}}}_{1:E}^{(\,{\rm{prop}}\,,t+1)}={{{\bf{x}}}}_{1:E}^{(t)}+{\tau }^{(t)}{\nabla }_{{{{\bf{x}}}}_{1:E}}\log | {\Psi }_{{{\boldsymbol{\theta }}}}|+\sqrt{{\tau }^{(t)}}{\xi }^{(t)}$$

with a Metropolis–Hastings accept/reject step. However, we often observed that the step size *τ* must become very small in order to maintain a reasonable acceptance rate, leading to unacceptably slow burn-in.

Taking our inspiration from the use of Langevin samplers in the generative modelling community, we propose an initial sampling phase using the Unadjusted Langevin Algorithm (ULA) that simply omits the accept/reject step. We combine ULA with step size annealing, and find that this algorithm very rapidly converges to electron samples close to the desired distribution. We then switch to MALA towards the end of equilibration to converge to exactly the right distribution. Supplementary Note 4 contains an ablation study showing the much faster burn-in from using this ULA/MALA strategy.

### Multiple determinants

Unlike much prior work in deep QMC, we do not use Hartree–Fock as a starting point for our ansatz. We found that Hartree–Fock is unreliable for the multireference structures that we are interested in. However, we noticed that training an ansatz from random initialisation led to worse performance, even in the limit of long training. We were able to trace this problem to ‘determinant collapse’—during early phases of training from random initialisation, we saw that one determinant rapidly came to dominate the model, leading to an effective single-determinant model that persisted to the end of training. Indeed, we verified that the worse energy that was obtained without initialising to Hartree–Fock was the same energy that was obtained from a single-determinant version of the model (whether or not Hartree–Fock initialisation was used).

To rectify this problem without the use of a Hartree–Fock baseline, we introduce a penalty term that is minimised when all determinants contribute equally to the wavefunction value

8 $${{{\mathcal{L}}}}_{{{\rm{det}}}}=-{\uplambda }_{{{\rm{det}}}}{\mathbb{E}}\left[\sum_{d}\log \left(\frac{\det [{A}^{d}]}{{\sum }_{d^{\prime} }\det [{A}^{d^{\prime} }]}\right)\right].$$

This penalty term can be directly backpropagated to compute the penalised training gradient.

Training with this penalty switched on, we discovered that starting from a randomly initialised network, we could recover the performance that was obtained when starting from a network initialised to the Hartree–Fock solution. Full details of this ablation study are presented in Supplementary Note 5.3.

### Light atom curriculum

The Light Atom Curriculum (LAC) consists of molecules of up to 24 electrons containing the species H, Li, B, C, N, O, and F. We chose H, C, N, O and F as a basic set of atoms that define a rich space of organic chemistry. We added Li and B to cover molecules beyond organic chemistry, whilst keeping to light atoms. This choice of atoms allows us to convincingly demonstrate that Orbformer can generalise across a large chemical space, whilst keeping the number of electrons low enough to avoid spiralling computational cost in the pretraining. We note that our pretrained Orbformer checkpoint is only suitable for molecules containing these atomic species. On the other hand, it is designed to generalise to systems much larger than 24 electrons, as shown in our experiments.

With the basic set of molecules defined, we sought to generate a more diverse set of geometries than previously considered—moving away from equilibrium geometries to more distorted structures where electronic structure is more likely to exhibit multireference character. We created five distortion operations that could be randomly sampled without human intervention: bond bending, bond breaking, bond stretching, bond bending & stretching, and bond bending & breaking. Additionally, we apply on-the-fly data augmentation to all our structures that consists of (i) random rotation, (ii) adding small Gaussian noise to all atom co-ordinates. We conducted an analysis on the LAC to establish whether multireference electronic structure is indeed likely to occur. On a small subset of our dataset, we computed the commonly used T1, D1 and ∣%TAE∣ diagnostics^42^ using CCSD(T)/cc-pVDZ. We found that between 20% and 45% of structures were considered multireference by these diagnostics, with structures that feature bond breaking the most highly represented in the multireference subset across all diagnostics.

At pretraining time, we took a curriculum learning approach^43^ that operated in three distinct phases, rather than pretraining on the entire LAC from the start. This approach enables us to pretrain efficiently by first learning features on very small molecules, where gradient steps are cheapest, before moving on to somewhat larger molecules. Thanks to our model architecture, features that we learn on the smallest molecules generalise to much larger ones. Indeed, whilst the largest pretraining molecules have 24 electrons, we fine-tune the resulting model on much larger molecules (up to 106 electrons in this paper). We also found that the curriculum learning approach improved pretraining stability. Full details of the curriculum learning are given in Supplementary Note 7.1 and details on the dataset composition and multireference diagnostics are presented in Supplementary Note 9.1.

## Supplementary information

Supplementary Information

Transparent Peer Review file

## Source data

Source Data

## Data Availability

The data that support this project are available at https://github.com/microsoft/oneqmc^44^. Source data are provided in this paper.

## Code availability

The code that supports this project and the model checkpoint that serves as a starting point for users’ calculations are available at https://github.com/microsoft/oneqmc^44^.
