# Supplementary material for: An ab initio foundation model of wavefunctions that accurately describes chemical bond breaking
Source: Nat Commun. 2026 Aug 21;17:9976. doi: 10.1038/s41467-026-76604-2 (PMC13590640; doi:10.1038/s41467-026-76604-2)
Supplement: Supplementary file 1 [file 41467_2026_76604_MOESM1_ESM.pdf]

Supplementary Information

An ab initio foundation model of wavefunctions that accurately describes chemical bond breaking

Suppl. Note 1. Glossary of mathematical symbols

Symbol	Meaning
E	Number of electrons
N	Number of nuclei
$\mathbf{r}$	Electron spatial coordinates
σ	Electron z-axis spin
$\mathbf{x}$	Electron spin-spatial coordinates, $(\mathbf{r}, \sigma)$
$\mathbf{R}$	Nucleus spatial coordinates
Z	Nucleus charge
$\mathbf{M}$	Molecular configuration, $(\mathbf{R}_{1:N}, Z_{1:N})$
$\hat{H}$	Hamiltonian
$\mathcal{E}$	Energy
Ψ	Wave function
$\boldsymbol{\theta}$	Ansatz parameters
$p(\cdot)$	Probability density
A	Slater matrix
S_z	Total z-axis spin of electrons in a molecule
O	Number of orbitals
F	Number of features
H	Number of heads
J	Jastrow factor
α	Jastrow parameters
Ω	Envelope matrix
Φ	Projected electron representation matrix
ζ	Envelope exponent
P	Primitive envelopes, shape $[E, N, F_{\text{env}}]$

Symbol	Meaning
W	Linear weight matrix
T	Electron representations, shape $[E, F_{\text{repr}}]$
φ	Featurization filter function
β, γ	Featurization parameters
κ	Attention distance bias weight
Q	Orbital representations, shape $[O, N, F_{\text{orb}}]$
$\boldsymbol{\eta}$	Initial orbital representations
τ	MCMC step size
ξ	Standard Gaussian random variable $\sim N(0, 1)$
B	Batch size
V	Potential
$\mathcal{L}$	Loss
π_{det}	Determinant relative probabilities
λ	Regularization weight
s	Standard deviation
ℓ_δ	Huber loss function, by default $\delta = 1\,\text{Ha}$
$\boldsymbol{\mathcal{Q}}$	Attention queries
$\boldsymbol{\mathcal{K}}$	Attention keys
$\boldsymbol{\mathcal{V}}$	Attention values
$\boldsymbol{\mathcal{B}}$	Attention edge biases
$\boldsymbol{\mathcal{O}}$	Attention outputs
$\boldsymbol{\mathcal{S}}$	Attention intermediate inner products
$\boldsymbol{\mathcal{P}}$	Attention weights

Suppl. Note 2. Background

2.1 Variational Monte Carlo (VMC)

We consider a molecular system comprising E electrons at positions $\mathbf{r}_1, \ldots, \mathbf{r}_E$ with spins $\sigma_1, \ldots, \sigma_E$, denoting their spatial-spin coordinates $(\mathbf{r}_e, \sigma_e) = \mathbf{x}_e$, and N nuclei at positions $\mathbf{R}_1, \ldots, \mathbf{R}_N$ with charges $Z_1, \ldots, Z_N$, which we refer to as the molecular configuration $(\mathbf{R}_{1:N}, Z_{1:N}) = \mathbf{M}$. If we make the Born–Oppenheimer approximation that the nuclei are fixed point charges, the stationary quantum states for the electrons satisfy the time-independent Schrödinger equation $\hat{H}|\Psi\rangle = \mathcal{E}|\Psi\rangle$ for the molecular Hamiltonian

$$\hat{H} := -\frac{1}{2}\nabla^2_{\mathbf{r}_{1:E}} + \sum_{n=1}^{N}\sum_{n'<n} \frac{Z_n Z_{n'}}{\|\mathbf{R}_n - \mathbf{R}_{n'}\|} - \sum_{n=1}^{N}\sum_{e=1}^{E} \frac{Z_n}{\|\mathbf{R}_n - \mathbf{r}_e\|} + \sum_{e=1}^{E}\sum_{e'<e} \frac{1}{\|\mathbf{r}_e - \mathbf{r}_{e'}\|} \tag{1}$$

in atomic units. Additionally, as electrons are fermions, electronic wave functions must be antisymmetric under the exchange of any two electrons.

The ground state wave function is the eigenstate with the lowest eigenvalue. In molecular variational Monte Carlo in first quantization, we propose an anti-symmetric, parametric, real-valued [1] wave function ansatz $\Psi_{\boldsymbol{\theta}}(\mathbf{x}_1, \mathbf{x}_2, \ldots, \mathbf{x}_E) \in \mathbb{R}$. The ground state can then be approximated by minimizing the Rayleigh quotient with respect to the parameter $\boldsymbol{\theta}$

$$\boldsymbol{\theta}^\star = \min_{\boldsymbol{\theta}} \frac{\langle \Psi_{\boldsymbol{\theta}} | \hat{H} | \Psi_{\boldsymbol{\theta}} \rangle}{\langle \Psi_{\boldsymbol{\theta}} | \Psi_{\boldsymbol{\theta}} \rangle} = \min_{\boldsymbol{\theta}} \int \frac{|\Psi_{\boldsymbol{\theta}}(\mathbf{x}_{1:E})|^2}{\langle \Psi_{\boldsymbol{\theta}} | \Psi_{\boldsymbol{\theta}} \rangle} \frac{\hat{H}\Psi_{\boldsymbol{\theta}}(\mathbf{x}_{1:E})}{\Psi_{\boldsymbol{\theta}}(\mathbf{x}_{1:E})} d\mathbf{x}_{1:E}. \tag{2}$$

The right-hand side of equation (2) shows how this quotient can be approximated by Monte Carlo integration using samples from the distribution $p(\mathbf{x}_{1:E}) \propto |\Psi_{\boldsymbol{\theta}}(\mathbf{x}_{1:E})|^2$. This stochastic approximation lifts the requirements of analytical integrability, allowing VMC ansätze to explicitly model electronic correlation.

Traditional VMC uses ansätze built from Slater determinants of single-particle orbitals. Explicit electron correlation is typically introduced by the inclusion of Jastrow factors, totally symmetric functions under the exchange of electrons, and back-flow transformations, totally symmetric displacements of the electron positions based on the remaining electrons [2].

More recently, neural network ansätze were proposed [3], including PauliNet [4] and FermiNet [5] as the first high-accuracy solutions for molecular ground states via the ab-initio neural network approach. Various new network architectures and algorithmic improvements have since been proposed to further improve accuracy and efficiency [6–14].

From the perspective of deep learning, three salient points of difference between training (fermionic) neural network wave function ansätze and training standard neural networks are

1. the network architecture must enforce the anti-symmetry constraint,
2. the training data is *self-generated* [15] using Markov Chain Monte Carlo (MCMC) to compute the loss (2), so no external source of data beyond the equations of physics is required, and
3. the loss function involves the Laplacian of the wave function with respect to the electron co-ordinates.

2.2 Orbital localization and size consistency

In classical quantum chemistry, the term *orbital* generally refers to a single-particle function $\psi(\mathbf{x}_e)$. In the context of molecular orbital theory [16], a localized orbital [17] is a single-particle orbital that is significantly different from zero only when $\mathbf{r}_e$ lies within a small region of space, e.g. close to a specific nucleus or between a pair of nuclei. By default, most classical quantum chemistry methods operate with non-localized orbitals that have support over the entire molecule [18]. However, exploiting the "nearsightedness of electronic matter", localized orbitals have been employed to scale classical quantum chemistry methods to very large systems [19–21].

The term *size consistency* generally refers to two non-interacting subsystems [22], such as two molecules $\mathbf{M}_1$ and $\mathbf{M}_2$ separated by a large distance. A method is said to be size consistent if the energy is additive for these non-interacting systems: $\mathcal{E}(\mathbf{M}_1)+\mathcal{E}(\mathbf{M}_2) = \mathcal{E}(\mathbf{M}_1+\mathbf{M}_2)$. Since the interaction terms in the molecular Hamiltonian (1) tend to zero in the large distance limit, size consistency will hold if the wave function can be expressed as a tensor product of non-interacting wave functions of the subsystems $|\Psi_{\mathbf{M}_1+\mathbf{M}_2}\rangle = |\Psi_{\mathbf{M}_1}\rangle \otimes |\Psi_{\mathbf{M}_2}\rangle$, which can be suitably antisymmetrized. Perhaps the most straightforward approach to constructing a size consistent ansatz is to employ a single Slater-determinant built from orbitals *localized* on either one of the two subsystems, with the correct number of orbitals assigned to each. Then, if the subsystems are sufficiently distant, the determinant will factorize into the above-mentioned tensor product form. We pick up on this thread in the next section when we discuss composability.

Suppl. Note 3. Orbformer Architecture

3.1 Basic principles and desiderata for a transferable ansatz

The original work on molecular ground states with neural network ansätze [4, 5] trained a fresh network for each molecule and each molecular geometry, so $\mathbf{M}$ were treated as configuration parameters and not inputs to the network. Given the remarkable capacity of neural networks to generalize, it makes intuitive sense to attempt the construction of a *transferable ansatz*, $\Psi_{\boldsymbol{\theta}}(\mathbf{x}_{1:E} \mid \mathbf{M})$, which takes the molecular configuration as an input. However, the architectural changes required to implement a truly transferable ansatz are significant.

To discuss such issues in more detail, we introduce the basic form of the Orbformer wavefunction

$$\Psi_{\boldsymbol{\theta}}(\mathbf{x}_{1:E} \mid \mathbf{M}) = e^{J_{\boldsymbol{\theta}}(\mathbf{x}_{1:E})} \sum_d \det[A^d_{\boldsymbol{\theta}}(\mathbf{x}_{1:E} \mid \mathbf{M})]. \tag{3}$$

In common with recent ansätze [4, 5, 14, 23, 24], Orbformer combines the sum of determinants of several all-electron $E \times E$ Slater matrices, $A^d_{\boldsymbol{\theta}}$, with a scalar Jastrow factor, $J_{\boldsymbol{\theta}}$. In contrast to traditional Slater-determinants built from single particle orbitals, these generalized Slater matrices contain permutation equivariant many body functions, composed of orbital envelopes and a projected latent space encoding of electrons by deep neural networks.

Differently sized inputs. For a chemically transferable ansatz, not only do the values of inputs $\mathbf{R}_{1:N}, Z_{1:N}$ and $\mathbf{x}_{1:E}$ change, but so do the number of those inputs E and N. The network must therefore admit a variable number of inputs and construct Slater matrices with variable sizes. To build such a model that can be JAX-compiled [25], we take an approach based on masking. For a given compilation of the model, we first set maximum particle numbers $E_{\max}$ and $N_{\max}$. Any non-existent particles are masked out, with the model adapting different operations accordingly. For example, the $E \times E$

Slater matrices are promoted to $E_{\max} \times E_{\max}$ matrices, with non-existent electrons filled in with identity submatrices, thereby preserving the determinant value.

Parameter dimensions. We further require that no parameter size depends on the number of electrons or nuclei, nor on $E_{\max}$ or $N_{\max}$. This is particularly beneficial when we train on a dataset of small molecules, setting $E_{\max}$ and $N_{\max}$ small to reduce computation time, but then transfer those parameters to a larger molecule.

Nuclei permutations. Considering the molecular configuration as an explicit input to the neural network we design the architecture to respect the molecular symmetries, i.e. permutations of the nuclei. While not a strict necessity, the permutation invariance helps with generalization, removing arbitrary indexing of nuclei.

Orbformer is implemented in such a way that a permutation of the nuclei may, at worst, lead to an arbitrary permutation of the *columns* of $A^d_{\boldsymbol{\theta}}$ (the same permutation across all electrons and all determinants), leading to a physically unimportant possible change of overall sign.

Composability. We use the term composability to refer to a property that is related to, yet distinct from, the classical concepts of orbital localization and size consistency (cf. Supplementary Note 2.2).

In Orbformer, single-particle orbitals are replaced with many-body orbitals, functions that depend on the positions of all electrons, constructed so that the rows of each Slater matrix are equivariant to electron permutations via the formula $\mathbf{A}^{d,e}_{\boldsymbol{\theta}}(\mathbf{x}_e, \{\mathbf{x}_{\setminus e}\} \mid \mathbf{M})$. Extending the idea of orbital localization, a composable many-electron orbital with input $(\mathbf{x}_e, \{\mathbf{x}_{\setminus e}\})$ should be non-zero only when an electron $\mathbf{r}_e$ is found in a small region of space, and additionally the dependence of its value on the positions of other electrons should decay as the distance between that electron, $\mathbf{r}_e$, and other electrons increases. Note that, by construction, electrons are indistinguishable and in practice there is no assignment of specific electrons to orbitals.

Without being fully prescriptive, we add a constraint to guarantee size consistency for a single-determinant Orbformer. If we consider two electrically neutral molecules $\mathbf{M}_1$ and $\mathbf{M}_2$ that are separated by a large distance, then we require E_1 orbitals to be localized to $\mathbf{M}_1$ and E_2 orbitals to be localized to $\mathbf{M}_2$ in the combined system $\mathbf{M}_1 + \mathbf{M}_2$. We also require that the value of the orbitals localized to $\mathbf{M}_1$ is independent of the nucleus and electron positions at $\mathbf{M}_2$ and is the same as would be obtained when running the ansatz on $\mathbf{M}_1$ alone, and *vice versa*. To see explicitly that this gives size consistency, let us also assume that the first E_1 electron samples are in the vicinity of molecule $\mathbf{M}_1$ whilst the latter E_2 electrons are near to $\mathbf{M}_2$. Then for a suitable ordering of the columns of $A^d_{\boldsymbol{\theta}}$ for the combined system, we will have

$$A^d_{\boldsymbol{\theta}}(\mathbf{x}_{1:E_1+E_2} \mid \mathbf{M}_1 + \mathbf{M}_2) = \begin{pmatrix} A_1 & 0 \\ 0 & A_2 \end{pmatrix} \tag{4}$$

where submatrices A_1 and A_2 should exactly match with what we would get if we evaluated Orbformer on either subsystem with the appropriate subset of electron inputs, i.e. $A_1 = A^d_{\boldsymbol{\theta}}(\mathbf{x}_{1:E_1} \mid \mathbf{M}_1)$ and $A_2 = A^d_{\boldsymbol{\theta}}(\mathbf{x}_{E_1+1:E_1+E_2} \mid \mathbf{M}_2)$. We also require that the Jastrow factor must be additive

$$J_{\boldsymbol{\theta}}(\mathbf{x}_{1:E_1+E_2}) = J_{\boldsymbol{\theta}}(\mathbf{x}_{1:E+1}) + J_{\boldsymbol{\theta}}(\mathbf{x}_{E_1+1:E_1+E_2}). \tag{5}$$

We stress that these result must hold for a single parameter $\boldsymbol{\theta}$ for the transferable ansatz evaluated on differently sized systems. We also note that the block diagonal form (4) depends on the arbitrary ordering of the orbitals and on the ordering of the electrons, so column and row swaps may be required to arrive at the block diagonal form.

This composability requirement gives size consistency for a single determinant model, because the determinant of the block diagonal Slater, (4), is a product of the determinants of the submatrices A_1 and A_2 and the exponential of the Jastrow factor, (5), is also a product, so that $\Psi_{\boldsymbol{\theta}}$ decomposes into the product of wavefunction values for subsystems $\mathbf{M}_1$ and $\mathbf{M}_2$. Row and column swaps lead, at worst, to an overall change of sign. Hence for fixed set of model parameters, single-determinant Orbformer *is* size consistent.

Composability does not lead to size consistency for multi-determinant wavefunction models because the sum of products is not equal to the product of sums. We were not able to find a simple way to obtain zero-shot size consistency whilst using a fixed number of determinants for differently sized systems. To the best of our knowledge, traditional size consistent quantum chemistry methods either use a single determinant (e.g. Hartree–Fock), or use an exponential ansatz parametrization at the cost of losing direct access to the wavefunction.

Previous work on transferable deep QMC also discussed size consistency [24]. We note that the conclusions of Gao & Günnemann [24] are similar to ours in the sense that size consistency is only guaranteed for a single determinant model but they used a multi-determinant ansatz in practice. Our approach to implementing localized orbitals differs from the approach of Globe—rather than anchoring each orbital to a single spatial location, from which localization can be defined, we allow our orbitals to spread across multiple nuclei; however, we limit the distance that messages can propagate to

maintain localization. We also note that Gao & Günnemann [24] state all inter-particle interactions decay exponentially in their model; however, by the inclusion of the Jastrow factor of Glehn et al. [14], this is not true, since

$$\exp\left(-c\,\frac{\alpha^2}{\alpha+r}\right) \sim 1 - c\,\frac{\alpha^2}{\alpha+r} \text{ as } r\to\infty. \tag{6}$$

An amended Jastrow similar to the one we use in Orbformer would be required to give an exponential decay rate.

Geometric invariances. Orbformer consumes only relative positions of particles, e.g. $\mathbf{r}_e - \mathbf{R}_n$, which grants us invariance to an overall translation of the system. We consciously choose not to incorporate any special (equivariant) treatment of rotations into our ansatz. While any observable of the molecule is indeed invariant under symmetry transformations of the nuclei accord to Curies principle, the wavefunction itself, not being an observable but a mathematical construct, *cannot be treated* as invariant. The ground state wavefunction of the boron atom serves as an illustrative example. In this system, the equation $\hat{H}|\Psi\rangle = \mathcal{E}|\Psi\rangle$ admits multiple, linearly independent solutions with the same ground state energy. Each solution corresponds to a particular physical orientation of the angular momentum vector of the electronic state. Thus, whilst the ensemble of all solutions is rotation invariant, training a network towards a specific solution requires us to be able to break rotation symmetry.

Spin assignment. Orbformer is a spin-assigned ansatz [26, 27], in which the z component of the electron spins are fixed for a given system throughout training. For transferability, we further simplify to the case in which the number of up- and down-spin electrons are equal (E even) or there is one more up-spin electron (E odd). This latter simplification does not prevent us from expressing wavefunctions with arbitrary total spins, as wavefunctions with $S_z = 0$ or 1 exist for all possible total spin quantum numbers.

Limitation of our formulation. In our work, we treat the nuclear co-ordinates and charges as a complete description of the molecule. This means that we do not consider learning systems with an overall charge (ions), with a spin state different from $S_z = 0$ or $S_z = \frac{1}{2}$ (not a restriction on S^2), and excited states. None of these are fundamental limitations of the ideas we present, but we leave them as topics for future work.

3.2 Architecture overview

As discussed in Supplementary Note 3.1, the Slater matrices $A^d_{\boldsymbol{\theta}}$ have *rows* $\mathbf{A}^{d,e}_{\boldsymbol{\theta}}$ that permute with the ordering of the input electrons $\mathbf{x}_1, \ldots, \mathbf{x}_E$, whilst the columns do not. As a notational aid, we henceforth denote the shape of the Slater matrices as $E \times O$, where O refers to 'orbitals' in a generalized sense. Whilst it must be the case that $O = E$, this choice of notation helps to separate electrons and orbitals logically. The overall structure of Orbformer, using this notation and for a single determinant, is shown in Fig 1.

Jastrow factor. The Jastrow factor is given by

$$J_{\boldsymbol{\theta}}(\mathbf{x}_{1:E}) = -\frac{1}{4}\sum_{e<e',\sigma_e=\sigma_{e'}}^{E} \alpha_{\text{par}} e^{-\|\mathbf{r}_e-\mathbf{r}_{e'}\|/\alpha_{\text{par}}} - \frac{1}{2}\sum_{e<e',\sigma_e\neq\sigma_{e'}}^{E} \alpha_{\text{anti}} e^{-\|\mathbf{r}_e-\mathbf{r}_{e'}\|/\alpha_{\text{anti}}} \tag{7}$$

where $\alpha_{\text{par}}, \alpha_{\text{anti}}$ are learnable parameters. Like earlier works [4, 14], this Jastrow factor ensures the correct electron–electron cusps for the wavefunction [28]. Unlike earlier works, we use an exponentially decaying function as opposed to a harmonically decaying one. Whilst both forms of the Jastrow factor do satisfy the composability requirement (5), we found that the exponential form was necessary for additivity of the Jastrow to also hold at finite distances where we would not expect an appreciable interaction to occur (e.g. 12Å).

Slater matrices. The matrices $A^d_{\boldsymbol{\theta}}$ are constructed as a pointwise product $A^d_{\boldsymbol{\theta}} = \Omega^d_{\boldsymbol{\theta}} \odot \Phi^d_{\boldsymbol{\theta}}$ of envelopes, Ω, and projected electron representations, Φ.

Envelopes. Our orbitals are a development of 'simplified envelopes' [8]. For each nuclear charge $1, \ldots, Z_{\max}$ we associate a set of learnable exponents $\zeta^Z_{1:F_{\text{env}}}$. The primitive envelopes are then formed as $P_{enf} = e^{-\zeta^{Z_n}_f \|\mathbf{r}_e - \mathbf{R}_n\|}$. As an output of the Orbital Generator network, we obtain coefficient matrices $W^{\text{env},\uparrow}_{donf}, W^{\text{env},\downarrow}_{donf}$ where d, o, n, f index the Slater matrices, orbitals, nuclei and primitive envelope features respectively. We then form the envelopes as a contraction

$$(\Omega^d_{\boldsymbol{\theta}})_{eo} = \sum_{n=1}^{N}\sum_{f=1}^{F_{\text{env}}} P_{enf} W^{\text{env},\sigma_e}_{donf}. \tag{8}$$

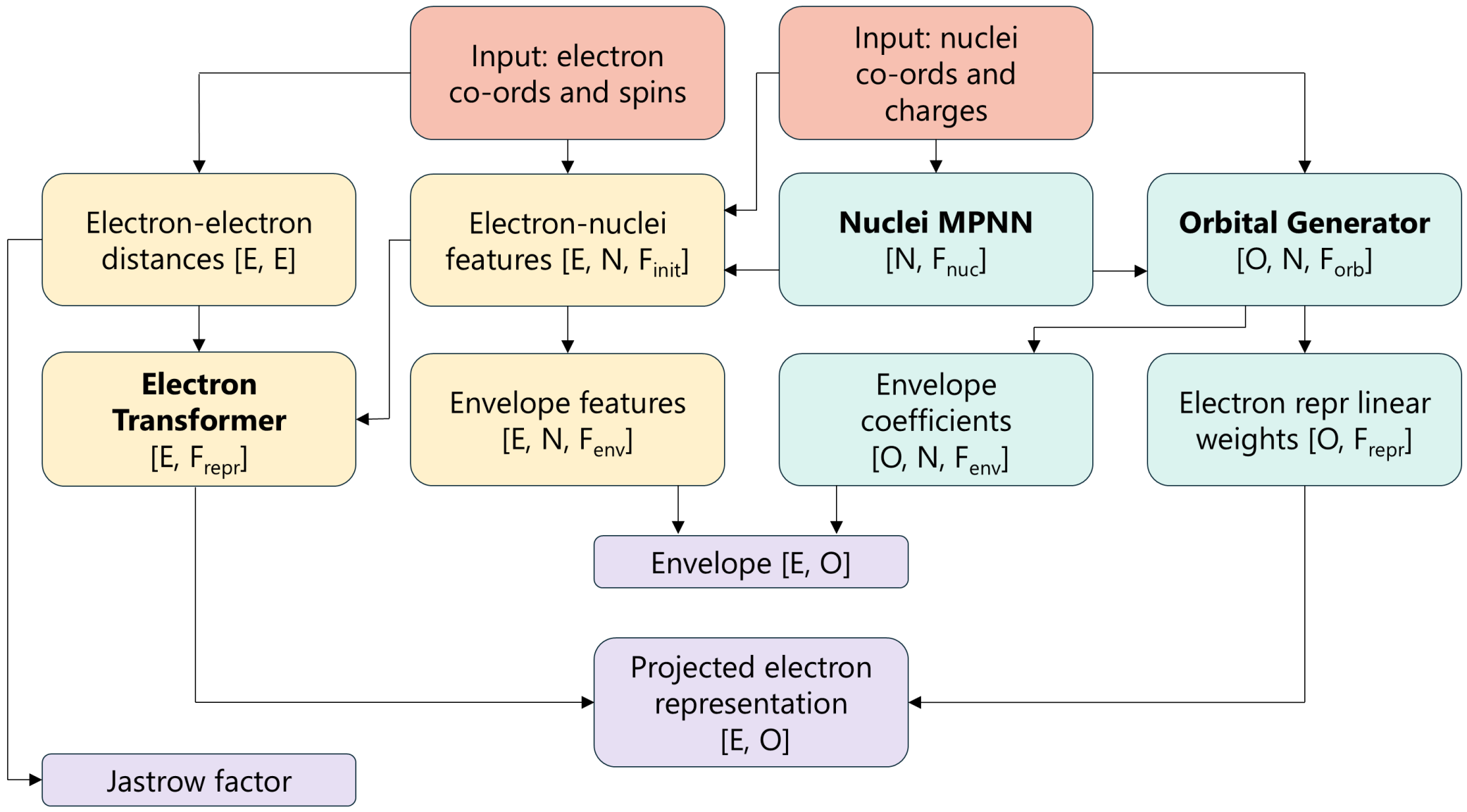

Supplementary Figure 1 | Orbformer overall architecture. Shape hints are written in terms of the maximum number of Nuclei, Electrons, Orbitals and various Feature dimensions. Determinant dimensions are not included on this diagram. See Supplementary Figs. 2 and 3 for detailed descriptions of the Electron Transformer, Orbital Generator and Nuclei MPNN subnetworks. Cyan denotes components of the network that do not depend on the electron inputs, $\mathbf{x}_{1:E}$.

We explored making the exponents ζ the output of either the Orbital Generator or the Nuclei MPNN, but found that this was prone to training instabilities. We also apply a transform that ensures $\zeta > 0.1$ throughout training. Finally, we apply an initialization scheme for ζ inspired by single electron wavefunctions for atomic systems

$$\zeta^{Z}_{f,\mathrm{init}} = \max(1, Z/f) \text{ for } f = 1, \dots, F_{\mathrm{env}}. \tag{9}$$

Projected electron representations. As an output of the Electron Transformer we have representations $T_{ef}(\mathbf{x}_{1:E} \mid \mathbf{R}_{1:N}, Z_{1:N})$ where T is constructed using a Transformer architecture [29] to be equivariant to permutations of the electrons, and $f = 1, \dots, F_{\mathrm{repr}}$. From the Orbital Generator we obtain coefficient matrices $W^{\mathrm{repr},\uparrow}_{dof}, W^{\mathrm{repr},\downarrow}_{dof}$ and then form

$$(\Phi^{d}_{\boldsymbol{\theta}})_{eo} = \sum_{f=1}^{F_{\mathrm{repr}}} T_{ef} W^{\mathrm{repr},\sigma_e}_{dof}. \tag{10}$$

3.3 Electron Transformer

The structure of this subnetwork is shown in Supplementary Fig. 2. Our use of a self-attention mechanism for electron representations is inspired by Psiformer [14], but with several innovations necessary to satisfy the requirements laid out in Supplementary Note 3.1.

Electron–nucleus feature extraction. We use distance-dependent filter functions $\varphi(\|\mathbf{r}_e - \mathbf{R}_n\|)$ to weight electron–nuclei interactions. In contrast to Psiformer, which uses a logarithmic rescaling, we focus on filters such that $\varphi(r) \to 0$ as $r \to \infty$. We found this made composability much easier to implement, because when summing signals from all nuclei we are guaranteed that distant nuclei contribute very little.

We use the following two families of filters

$$\varphi_{\exp}(r \mid \beta) = \beta e^{-\beta r}, \tag{11}$$

$$\varphi_{\mathrm{sigmoid}}(r \mid \gamma) = \frac{1}{\gamma(1 + e^{r-\gamma})}, \tag{12}$$

and we selected non-learnable values $\beta = 1, 3; \gamma = 2, 4$ Bohr. We found that it was important to incorporate filters that extend to a reasonable distance before decaying, which was the motivation for our sigmoid filters. We experimented with learning the β and γ values, but found it was more unstable. Importantly, our filters are non-zero at the origin with non-zero gradient, and thus enable the network to correctly model the nuclear cusp in the same way as the Psiformer features.

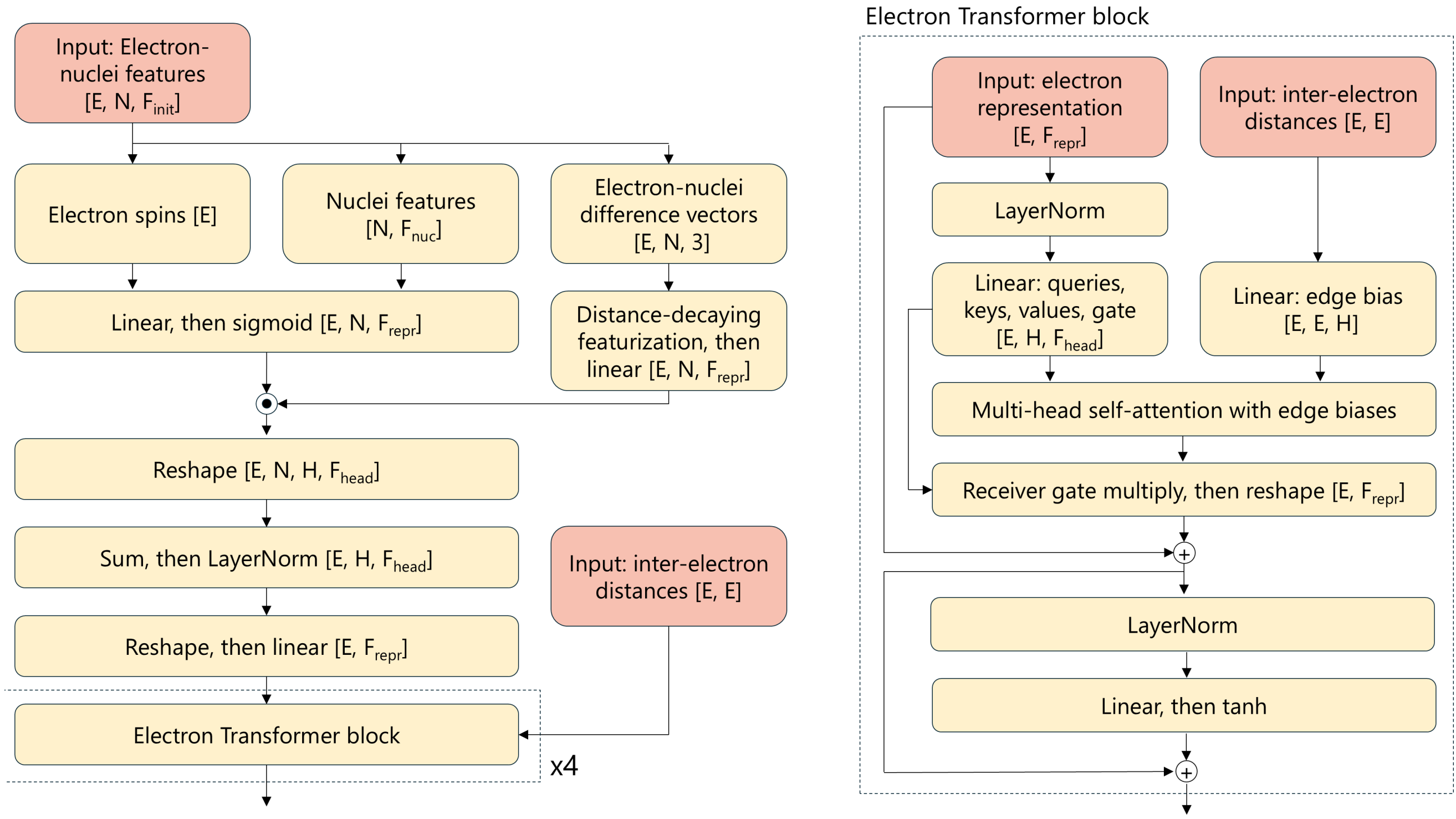

Supplementary Figure 2 | Electron Transformer architecture. Shape hints are given as in Supplementary Fig. 1, and H denotes the number of heads.

For each pair (e, n), we then take the filter itself, along with the rescaled difference vector as features. To facilitate possible rotations based on the molecular geometry, we create matrices W_n^{rot} as an output of the Nuclei MPNN of shape 6×3 and incorporate the 6-dimensional 'rotated' features $W_n^{\text{rot}}(\mathbf{r}_e - \mathbf{R}_n)$, suitably multiplied by our filter functions. There is no constraint for W_n^{rot} to have orthonormal rows. Features from different filters are stacked and then passed through a linear layer.

Separately, we create a spin–nucleus feature using the electron spin and nuclei features from the Nuclei MPNN for every (e, n) pair, which are then passed through a single layer network. This spin–nucleus feature is then multiplied by the filter-based featurization of the vector $\mathbf{r}_e - \mathbf{R}_n$ to give our overall electron–nucleus feature.

Feature aggregation. The electron–nuclei features are summed over the nuclei axis to reduce them to electron feature vectors. The sum operation combined with distance-decaying featurization ensures composability. The exact summation procedure using multiple heads and LayerNorm [30] is shown in Supplementary Fig. 2.

Electron transformer block. Here we only discuss the main differences between a standard Pre-LN transformer block [31] and our block. First, we incorporate an edge bias based on the distance [32] between electrons. Denoting the distance between two electrons as $r_{ee'} := \|\mathbf{r}_e - \mathbf{r}_{e'}\|$ and for attention head h, we include the bias

$$\text{bias}_{ehe'} = -\log\left(1 + e^{r_{ee'} - 8}\right) - |\kappa_h r_{ee'}|. \tag{13}$$

The first term ensures an asymptotic decay rate $\sim e^{-r_{ee'}}$, guaranteeing that no information flows between distant electrons, which is essential for composability. The second uses a learnable coefficient κ_h to control the distance bias of a given head in the non-asymptotic region. Thus κ_h can be seen as controlling how 'myopic' a given attention head is.

Second, unlike in standard transformers, the output of the self-attention computation is multiplied by a receiver gate. Given electron representations $T_{ef}^{(\ell)}$ at layer ℓ, we apply self-attention over the electron axis, giving $T_{ef}^{(\ell,\text{attn})}$. We also apply a linear layer to $T_{ef}^{(\ell)}$ to give $T_{ef}^{(\ell,\text{gate})}$. The self-attention output is then reweighted by componentwise multiplication to give

$$T_{ef}^{(\ell,\text{gated attn})} = T_{ef}^{(\ell,\text{attn})} \odot \text{sigmoid}\left(T_{ef}^{(\ell,\text{gate})}\right). \tag{14}$$

We found that this gating leads to improvements in transferability performance. Our hypothesis is that the receiver gate allows electrons that are very close to a nucleus to ignore incoming updates from other electrons. Thirdly, we use a simplified tanh-activated MLP as in Psiformer, but we combine it with Pre-LN. See the right-hand side of Supplementary Fig. 2 for a complete description.

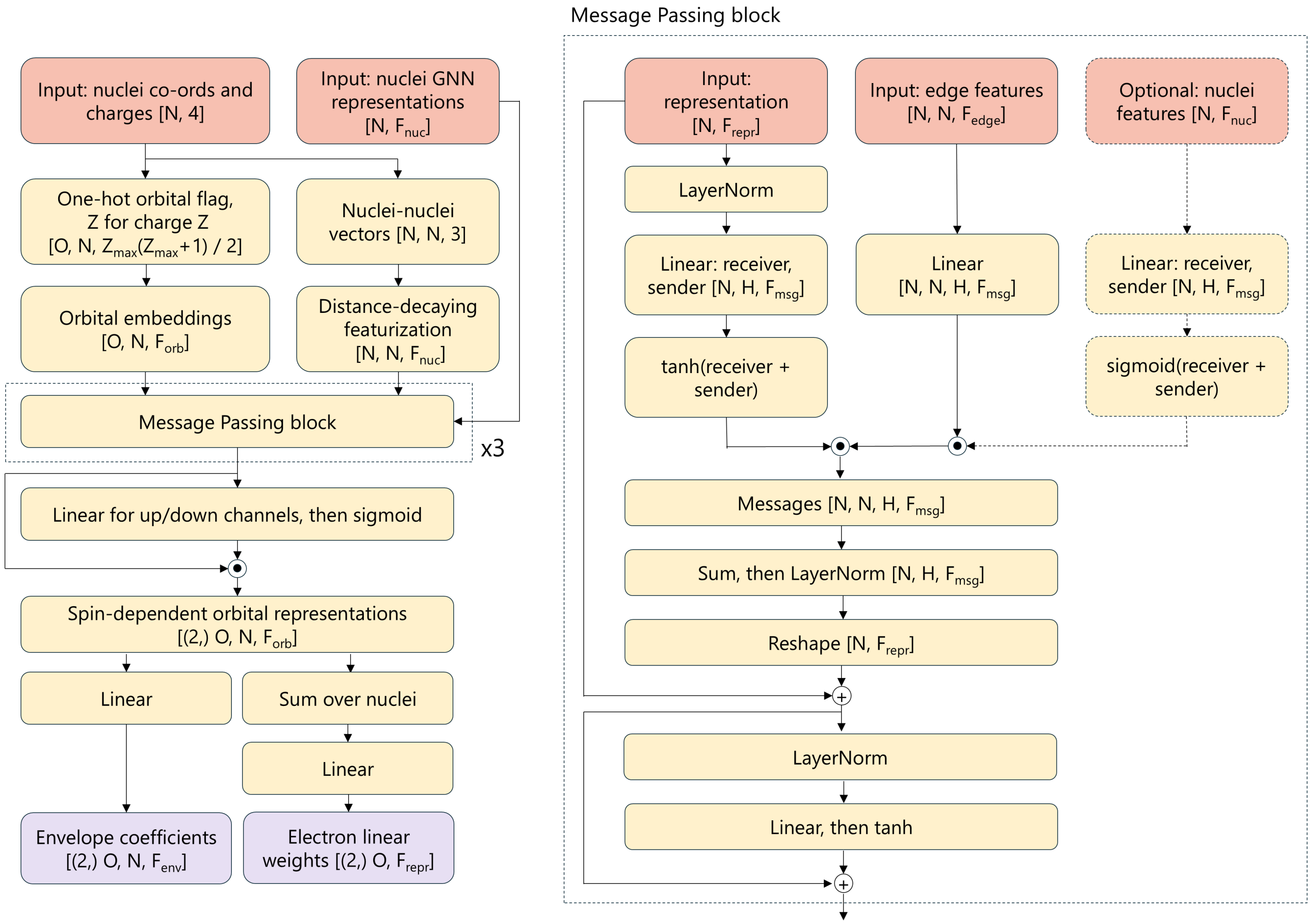

Supplementary Figure 3 | Orbital Generator architecture. Shape hints are given as in Supplementary Figs. 1 and 2. The Message Passing layers are completely parallel over the Orbital dimension. The Nuclei MPNN is a simplified version of this subnetwork without the orbital dimension. In the Nuclei MPNN, initial features are simple embeddings of the nuclear charge, the third input (nuclei features) in the Message Passing blocks is not present, and there are no separate spin-dependent representations formed at the end.

FlashAttention. We identified the attention layers as the most computationally demanding component of the entire Orbformer network. To accelerate the attention computations, we implement a variant of the FlashAttention algorithm [33] that is suitable for our architecture. We used `pallas` [34] for the implementation. Significantly, as well as acceleration of the forward and backward passes, we also develop a FlashAttention-inspired implementation for the Laplacian, see Supplementary Note 6.2 for full details.

3.4 Orbital Generator

The structure of the Orbital Generator is presented in Supplementary Fig. 3. At a high level, the tensors that propagate through the Orbital Generator network have shape $[O, N, F_{\text{orb}}]$. This means that each feature vector corresponds to an orbital–nucleus pair. The advantage of this approach is that it allows us to control the spatial extent of different orbitals and enforce orbital localization. This comes at the price of a relatively sparse orbital representation, particularly for larger molecules.

Initial orbital embeddings. Under the assumption of a molecule with no overall charge, the number of electrons, and hence the number of orbitals, must equal the sum of nuclear charges $\sum_{n=1}^{N} Z_n$. To featurize these orbitals, we use an algorithm inspired by the linear combination of atomic orbitals (LCAO) approach. Initially, the features of each orbital, which are of shape $[N, F_{\text{orb}}]$, are zero for all but one nucleus. We can therefore speak of an orbital being initialized 'on' a certain nucleus. Using the assumption of electric neutrality, we initialize exactly Z_n orbitals on nucleus n. Furthermore, we use a set of initial orbital embeddings that are determined solely by the atom type. This is accomplished through a set of learnable initial feature vectors $\boldsymbol{\eta}_i^Z$ with $Z = 1, ..., Z_{\max}$ defining the atom type, and $i = 1, ..., Z$ indexing that atom type's initial orbital embeddings.

Given an arbitrary ordering for the nuclei, we assign the first Z_1 orbitals to the first atom, the following Z_2 orbitals to the second, etc. Supposing the oth orbital is initially assigned as the ith orbital of the nth atom, it's initial embedding of shape $[N, F_{\text{orb}}]$ will be

$$Q^{(0)}_{on'f} = \left(\eta_i^{Z_n}\right)_f \delta_{nn'} \text{ where } \begin{cases} n = 1, \dots, N, \\ i = 1, \dots, Z_n, \\ o = i + \sum_{m=1}^{n-1} Z_m, \\ n' = 1, \dots, N, \\ f = 1, \dots, F_{\text{orb}} \end{cases} \tag{15}$$

where δ is the Kronecker delta, encoding the atom to which the given orbital is assigned and setting coefficients to zero on all other nuclei.

We note that the ordering of the orbitals depends on the ordering of the nuclei. It is this indeterminate ordering that gives rise to the possible permutation of the columns of the Slater matrices $A^d_{\boldsymbol{\theta}}$ that was discussed in Supplementary Note 3.1 'Nuclei permutations'.

Orbitals do not remain localized to a specific nucleus after initialization, instead we allow interaction through a sequence of message passing layers. These layers allow the fixed initial embeddings to slowly adapt to the overall molecular geometry. The message passing layers operate in parallel over the orbital dimension with information exchanged between different nuclei. We tried some limited experiments incorporating orbital–orbital interaction, but we did not find that it improved performance. Message passing steps on the nuclei axis utilize nucleus–nucleus edge features, which we now describe.

Nucleus–nucleus feature extraction. We re-use the filter-based featurization approach that was used for the electron–nuclei features in Supplementary Note 3.3. Specifically, we compute filters $\varphi(\|\mathbf{R}_N - \mathbf{R}_{N'}\|)$ using the exponential and sigmoid functions in (11) and (12). For the nuclei, we use non-learnable filter hyperparameters $\beta = 1; \gamma = 2, 4, 6$ Bohr. We set self-edges to 0. As with the electrons, we concatenate the filter itself with the rescaled difference vectors .We then concatenate over the different filter functions. We do not use the rotated features that were used in the Electron Transformer.

Orbital LayerNorm Since orbital representations are presumed to be sparse, applying standard LayerNorm in parallel over the orbitals and nuclei axes is not appropriate. Instead, we apply mean-centering as usual, and then rescale features using the maximum variance across nuclei within a given orbital

$$Q^{(\ell,\text{LN})}_{onf} = Q^{(\ell)}_{onf} \left(\max_{n'} \left[\frac{1}{F_{\text{orb}}} \sum_{f=1}^{F_{\text{orb}}} \left(Q^{(\ell)}_{on'f} \right)^2 \right] + \epsilon \right)^{-1/2}. \tag{16}$$

All references to LayerNorm in Supplementary Fig. 3 refer to this variant. Similarly, all linear layers have no bias unless otherwise specified, to retain sparsity.

Message passing block. We use a Message Passing Neural Network (MPNN)[35, 36] to exchange information using the set of nuclei $\mathbf{M}$, in parallel over different orbitals.

Our message passing layer is designed so that the message between nuclei n and n' on orbital o tends to zero if either

- the distance $\|\mathbf{R}_n - \mathbf{R}_{n'}\|$ is large, or
- the input features for both n and n' are zero on that orbital.

These restrictions mean that orbital information 'propagates outward' from the initial single nucleus centre of a certain orbital. To implement this concretely, we define the message $\mathbf{M}_{o,n\to n'}$ as the pointwise product of three terms

1. a linear function of the nuclei–nuclei edge features which decay to zero in the large-distance limit,
2. a linear function of the current representations of the receiver and sender with a tanh activation, $\tanh\left(W^{\text{sender}}\mathbf{Q}^{(\ell)}_{on} + W^{\text{receiver}}\mathbf{Q}^{(\ell)}_{on'}\right)$, which will be zero if both receiver and sender are zero, and
3. a linear function of nuclei embeddings for the receiver and sender nuclei (derived from the Nuclei MPNN), with a sigmoid activation.

Messages are split into multiple heads and summed across the sender dimension. Additionally, we use the same tanh-activated MLP layer with Pre-LN as we use in the Electron Transformer. Note that this gives a zero update when the input is zero, as required.

Orbital-dependent coefficients As a final step of the Orbital Generator, we apply two different gating layers to produce separate representations for up- and down-spin electrons, denoted Q^{σ}_{onf} where $\sigma = \uparrow, \downarrow$. The orbital representations are fed back into the main ansatz by extracting envelope coefficients $W^{\text{env},\sigma}_{donf}$ and electron representation coefficients $W^{\text{repr},\sigma}_{dof}$. We form $W^{\text{env},\sigma}_{donf}$ by applying linear layers to Q^{σ}_{onf}; we use different layers for the two spins, and for each determinant. For $W^{\text{repr},\sigma}_{dof}$, we first sum Q^{σ}_{onf} over the nuclei axis, and then apply a linear layer, with multiple layers for spins and determinants as before.

We found that with standard training from a random initialization, a single determinant rapidly dominates the others, leading to an effective single-determinant wavefunction. To partially mitigate this, we design an initialization scheme so that $W^{\text{env},\sigma}_{donf}$ and $W^{\text{repr},\sigma}_{dof}$ are (almost) equal for different determinants d at initialization. We defer a full discussion of this issue to Supplementary Note 5.3.

3.5 Nuclei MPNN

The Nuclei MPNN uses the same architecture as the Orbital Generator, but without the presence of the orbital axis. The initial representations are simple embeddings of atom types. We use standard LayerNorm because sparsity is not a requirement for this network. The Message Passing blocks use only two inputs: current nuclei representations and nucleus–nucleus edge features. The final nuclei representations are returned after the last Message Passing block. With these simplifications, we see that our Nuclei MPNN is similar to a standard MPNN for molecular graphs.

The matrices W^{rot}_{n} that are fed from the Nuclei MPNN into the Electron Transformer are simple linear projections of the final nuclei representations. In the Orbital Transformer, the nuclei representations are fed directly into each Message Passing layer as seen in Supplementary Fig. 3.

3.6 Single molecule mode

The three points of exchange between the nuclei-only parts of the network and the electron-dependent part of the network are: the nuclei representations, the envelope coefficients $W^{\text{env},\sigma}_{donf}$ and the electron representation linear weights $W^{\text{repr},\sigma}_{dof}$. When training or fine-tuning Orbformer on a single molecule, it is possible to make these tensors simple, trainable parameters. Thus, the Orbital Generator and Nuclei MPNN are simply used to generate *initializations* for these parameters, and these subnetworks can then be discarded. We found that this results in more memory efficient single-point fine-tuning, but that the number of gradient steps required to converge could be larger than when fine-tuning the entire network on a single molecule. This mode was only used to generate single-point reference deep QMC energies for the ethane, formamide, 1-propanol, 2-aminopropan-2-ol, and L-alanine dissociation curves presented in the main text. Single molecule mode is not suitable for training on multiple molecules simultaneously.

3.7 Table of architecture hyperparameters

Architecture settings are shown in Supplementary Table 2.

Supplementary Table 2 | Dimensions and parameter values used for the Orbformer architecture.

Component	Parameter	Value
Electron Transformer	Attention heads	8
	Featurization heads	16
	Feature dimension (concat over heads), F_{repr}	256
	Attention layers	4
	Featurization filters	4
	Initial electron–nuclei feature dimension, F_{init}	40
Orbital Generator	Message passing heads	4
	Feature dimension (concat over heads), F_{orb}	128
	Message passing layers	3
Nuclei MPNN	Message passing heads	2
	Feature dimension (concat over heads), F_{nuc}	64
	Message passing layers	3
Envelopes	Exponents per nucleus, F_{env}	8
Determinants	Number of determinants	16

3.8 Relation to existing transferable QMC methods

Orbformer builds on recent deep QMC work that has explored the possibility of transferability across molecules by taking the molecular configuration as input [37, 38], most notably Gao & Günnemann [24] and Scherbela et al. [23]. Here we summarize how the design of Orbformer relates to, and differs from, these existing efforts.

Orbital construction. Comparing to the Globe model [24], our orbital construction does not hardcode core, valence or bonding orbitals and avoids any discontinuities or jumps in the orbital representations. Thus, when we discuss 'core' orbitals in Sec. 2.5 of the main text, these are an emergent phenomenon rather than an architectural design choice. We achieve this by allowing a single atom type to define multiple initial orbitals, and relying on the message passing layers to identify 'bonding' orbitals if and when they exist. Our approach avoids assumptions that are associated with closed shell molecules, enabling us to better deal with bond dissociations. Comparing to Scherbela et al. [23], we do not use Hartree–Fock when setting orbital initializations. This allows our network greater flexibility and avoids entangling our orbital generation with any problems of Hartree–Fock in multireference settings. Our message passing algorithm also differs from all previous work in its specifics, such as the normalization layers and the formation of messages as a product of three terms (cf. Supplementary Note 3.4).

Composability and size consistency. Our approach to constructing localized orbitals is similar to Gao & Günnemann [24], who showed that the block diagonal form of a Slater matrix, (4), can be achieved using localization constraints. We prefer the term 'composability' to emphasize the fact that these constraints do not lead to size consistency in the multi-determinant case. We note some slight differences between the two formulations in Supplementary Note 3.1.

Suppl. Note 4. Sampling

4.1 Self-generating electron configuration data for transferable training

One of the most promising aspects of single-molecule VMC training is that training data is generated from the model itself and used to converge the ansatz towards the theoretical ground state wave function, as discussed in Supplementary Note 2.1. This property of VMC marks a major departure from statistical learning paradigms that currently predominate in machine learning for natural science.

Here, we consider extending the self-generative training procedure of VMC to train our transferable Orbformer ansatz across a region of chemical space. Specifically, we consider transferable training in which molecules $\mathbf{M}$ are sampled from a training distribution, p_{train}. The capability to train against an arbitrary training distribution allows us to contemplate using very large datasets of molecular structures with data augmentation. This is a significant change from previous work where Scherbela *et al.* [38] using a dataset of 700 molecular structures was the largest molecular dataset yet considered in the field, to the best of our knowledge.

In order to train on a dataset of unlimited size, we must find a way to self-generate training data for new, unseen molecules. That is, we must be able to sample $\mathbf{x}_{1:E} \sim |\Psi_{\boldsymbol{\theta}}(\mathbf{x}_{1:E} \mid \mathbf{M})|^2$ for a molecule $\mathbf{M}$ that has not been previously encountered during training. Previous work [23] side-stepped this problem by maintaining separate MCMC samplers for each training molecule in their training dataset, but this solution does not scale.

Multiple optimizer steps. The naive solution to this problem is to burn in new MCMC walkers from scratch at each training step. However, with the standard MCMC sampling approaches, the number of steps required to reach equilibrium is large and becomes even larger as molecule size increases. As a first step to reduce the cost of re-equilibration, we fix the training molecules $\mathbf{M}$ for multiple optimization steps, allowing us to re-use MCMC walkers that have already burned in by running only a few additional MCMC steps between optimizer steps on the same molecules.

Faster burn-in with Unadjusted Langevin Algorithm. Previous work has used either Metroplis–Hastings [39] with Gaussian proposals or Metroplis–Adjusted Langevin Algorithm (MALA) [40] to sample electron configurations. MALA combines a Langevin proposal with step-size τ

$$\mathbf{x}_{1:E}^{(\text{prop},t+1)} = \mathbf{x}_{1:E}^{(t)} + \tau^{(t)} \nabla_{\mathbf{x}_{1:E}} \log |\Psi_{\boldsymbol{\theta}}| + \sqrt{\tau^{(t)}} \xi^{(t)} \tag{17}$$

with a Metropolis–Hastings accept/reject step.

When using MALA, we observed that the step size τ must become very small in order to maintain a reasonable acceptance rate. Sample rejection also tends to slow down mixing. A large number of steps are therefore required to reach the equilibrium distribution.

In other fields of machine learning, Langevin sampling has seen remarkable success, for example, in the field of diffusion models [41]. These applications use Unadjusted Langevin Algorithm (ULA) [42, 43], i.e. they do not apply an accept/reject

step. In the transferable VMC context, we therefore propose an initial sampling phase using ULA with step size annealing [44, 45] to get close to the correct distribution. We switch to MALA towards the end of sampling to reach perfect equilibrium, and we use MALA exclusively when updating samplers between multiple optimization steps on the same molecules. The benefits of this strategy are illustrated in Supplementary Note 4.2.

Improved Langevin sampler. We made several other modifications to the standard algorithm. First, we apply norm clipping to the gradient term in (17). Second, we modify the gradients when electron samples are very near to nuclei to prevent electron samples passing 'through' a nucleus. Third, we observed that it is possible for some MCMC chains to get stuck in low probability regions. This is common in partially dissociated molecules, where the probability of having charges imbalanced between the dissociated fragments becomes low as the wave function is trained. Our solution to this problem is to simply remove walkers with very low $\log|\Psi_{\boldsymbol{\theta}}|$ values and replace them with a uniform sample from the surviving walkers. Given $\log|\Psi_{\boldsymbol{\theta}}|$ values $L^{(b)}$ for a batch $b = 1, \dots, B$, we let $L^{(\mathrm{med})}$ denote the median value over the batch and $L^{(\mathrm{spread})} = \frac{1}{B}\sum_{b=1}^{B}|L^{(\mathrm{med})} - L^{(b)}|$. We prune the walkers where $L^{(b)} < L^{(\mathrm{med})} - 4L^{(\mathrm{spread})}$.

Electron configuration sampling for fine-tuning When fine-tuning on single molecules or reaction curves, we use Metropolis–Hastings and we maintain separate MCMC chains for each molecule.

4.2 Details of ablation study comparing ULA and MALA sampling

For this study, we used a randomly initialized Orbformer network in single-molecule mode on the benzene molecule in its equilibrium geometry. When running ULA, we annealed the step size from 0.15 to 0.005 using a geometric scheduler. We clipped the gradient norm to a maximum of 5. As can be seen on Supplementary Fig. 4, a pure MALA sampler begins with a small step size and makes slow progress by first moving samples to a high probability region and then diffusing outwards. Initial ULA sampling very rapidly moves samples near to equilibrium, with MALA completing the equilibration.

Suppl. Note 5. Variational Training

5.1 The local energy

Where single-molecule VMC training approaches a ground state wave function by minimizing the energy, we train our transferable ansatz to jointly represent the ground state wave function for an entire class of molecules by minimizing the expected value of the energy over the training distribution of molecules. Following Supplementary Note 2.1, this expected energy can be written as a nested expectation

$$\mathcal{L}_{\mathrm{energy}} = \mathbb{E}\left[\mathcal{E}_{\mathrm{loc}}\left(\mathbf{x}_{1:E}, \mathbf{M}\right)\right] \tag{18}$$

where the expectation involves sampling molecules $\mathbf{M} \sim p_{\mathrm{train}}$ and then sampling electron configurations conditional on those molecules $\mathbf{x}_{1:E} \sim |\Psi_{\boldsymbol{\theta}}(\mathbf{x}_{1:E} \mid \mathbf{M})|^2$ using the technique outlined in Supplementary Note 4. The local energy is

$$\mathcal{E}_{\mathrm{loc}}\left(\mathbf{x}_{1:E}, \mathbf{M}\right) = \frac{\hat{H}\Psi_{\boldsymbol{\theta}}(\mathbf{x}_{1:E} \mid \mathbf{M})}{\Psi_{\boldsymbol{\theta}}(\mathbf{x}_{1:E} \mid \mathbf{M})}, \tag{19}$$

which can be rewritten more explicitly using (1) as

$$\mathcal{E}_{\mathrm{loc}}\left(\mathbf{x}_{1:E}, \mathbf{M}\right) = -\frac{1}{2}\frac{\nabla^2_{\mathbf{r}_{1:E}}\Psi_{\boldsymbol{\theta}}(\mathbf{x}_{1:E} \mid \mathbf{M})}{\Psi_{\boldsymbol{\theta}}(\mathbf{x}_{1:E} \mid \mathbf{M})} + V(\mathbf{x}_{1:E}, \mathbf{M}) \tag{20}$$

where ∇^2 is the Laplacian operator and V is the Coulomb potential. Whilst evaluating the potential given samples is fast, the computation of the Laplacian of the network $\Psi_{\boldsymbol{\theta}}$ with respect to the electron coordinate inputs is a significant computational bottleneck. Our efforts to improve the efficiency of the Laplacian computation are discussed in Supplementary Note 6 in detail.

5.2 Energy gradient computation

To compute the gradient of the energy expectation value it is common to employ the "gradient trick". Utilizing the hermiticity of the Hamiltonian, the naive gradient of the energy is replaced with an unbiased estimator that involves at most second derivatives (instead of third derivatives arising due to taking the derivative of the kinetic energy operator

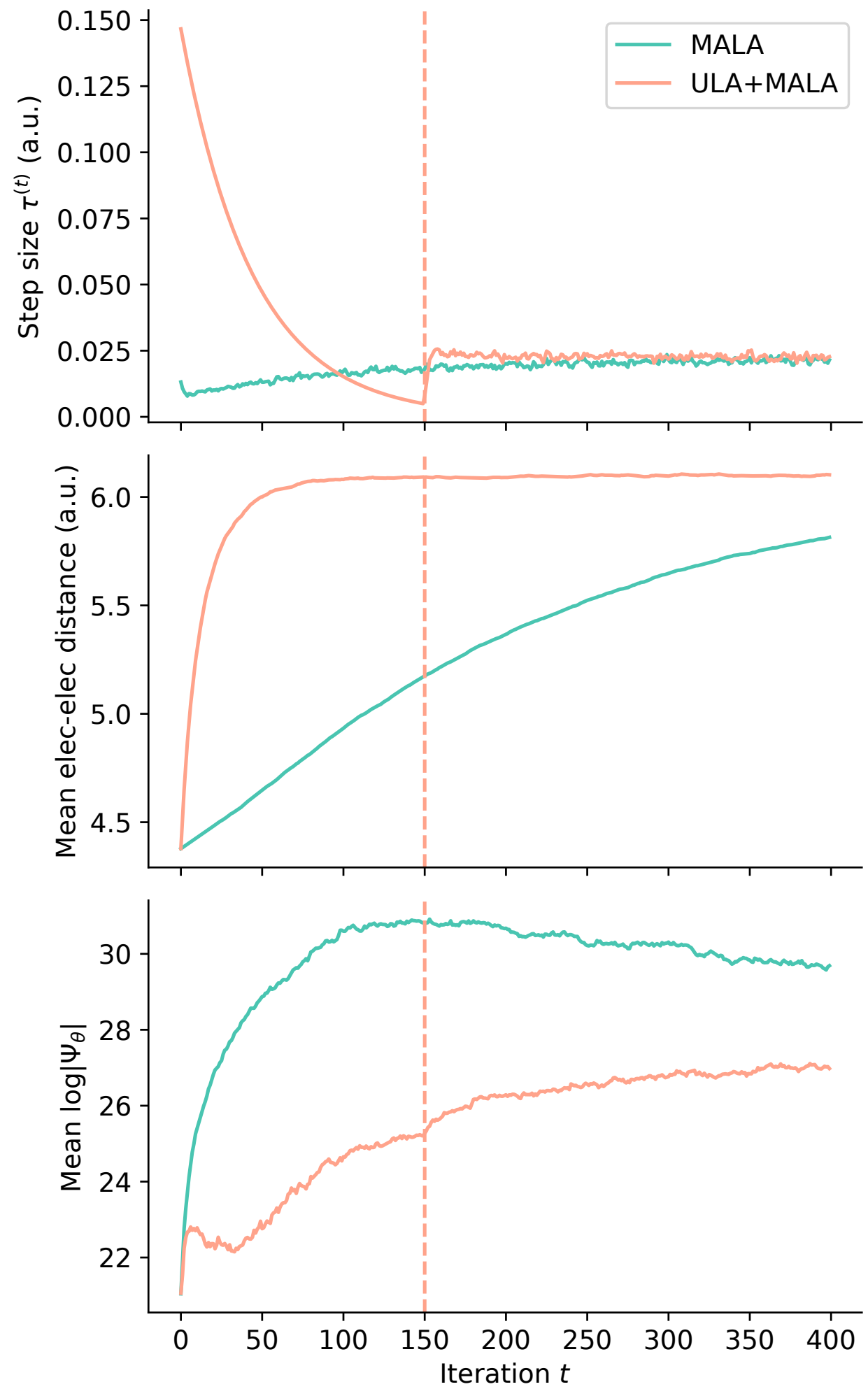

Supplementary Figure 4 | Illustrating the benefits of initial ULA sampling. We ran Langevin sampling with 512 parallel chains on a randomly initialized Orbformer wave function for the benzene molecule at equilibrium geometry. We ran standard MALA for 400 steps with a target acceptance of 57%, and we separately ran 150 steps of ULA followed by 250 steps of MALA. Distance units are Bohr (a.u.). Source data are provided as a Source Data file.

with respect to the ansatz parameters). For completeness we provide the derivation of the energy gradient

$$\nabla_{\boldsymbol{\theta}} \langle \hat{H} \rangle = \nabla_{\boldsymbol{\theta}} \frac{\int \Psi_{\boldsymbol{\theta}} \hat{H} \Psi_{\boldsymbol{\theta}}}{\int |\Psi_{\boldsymbol{\theta}}|^2} \tag{21}$$

$$= \frac{\int \nabla_{\boldsymbol{\theta}} \Psi_{\boldsymbol{\theta}} \hat{H} \Psi_{\boldsymbol{\theta}}}{\int |\Psi_{\boldsymbol{\theta}}|^2} - \frac{\int \Psi_{\boldsymbol{\theta}} \hat{H} \Psi_{\boldsymbol{\theta}}}{\int |\Psi_{\boldsymbol{\theta}}|^2} \frac{\int \nabla_{\boldsymbol{\theta}} \Psi_{\boldsymbol{\theta}}^2}{\int |\Psi_{\boldsymbol{\theta}}|^2} \tag{22}$$

$$= \frac{\int \Psi_{\boldsymbol{\theta}} \hat{H} (\nabla_{\boldsymbol{\theta}} \Psi_{\boldsymbol{\theta}}) + (\nabla_{\boldsymbol{\theta}} \Psi_{\boldsymbol{\theta}}) \hat{H} \Psi_{\boldsymbol{\theta}}}{\int |\Psi_{\boldsymbol{\theta}}|^2} - \langle \hat{H} \rangle \frac{\int 2 \Psi_{\boldsymbol{\theta}} (\nabla_{\boldsymbol{\theta}} \Psi_{\boldsymbol{\theta}})}{\int |\Psi_{\boldsymbol{\theta}}|^2} \tag{23}$$

$$= \frac{2 \int \Psi_{\boldsymbol{\theta}} \frac{\hat{H} \Psi_{\boldsymbol{\theta}}}{\Psi_{\boldsymbol{\theta}}} (\nabla_{\boldsymbol{\theta}} \Psi_{\boldsymbol{\theta}}) - 2 \mathcal{E} \ \Psi (\nabla_{\boldsymbol{\theta}} \Psi_{\boldsymbol{\theta}})}{\int |\Psi_{\boldsymbol{\theta}}|^2} \tag{24}$$

$$= \frac{2 \int \Psi_{\boldsymbol{\theta}}^2 (\frac{\hat{H} \Psi_{\boldsymbol{\theta}}}{\Psi_{\boldsymbol{\theta}}} - \mathcal{E}) \nabla_{\boldsymbol{\theta}} \log \Psi_{\boldsymbol{\theta}}}{\int |\Psi_{\boldsymbol{\theta}}|^2} \tag{25}$$

$$= 2 \mathbb{E}_{\Psi_{\boldsymbol{\theta}}^2} \left[\left(\mathcal{E}_{\text{loc}} - \mathcal{E} \right) \nabla_{\boldsymbol{\theta}} \log \Psi_{\boldsymbol{\theta}} \right], \tag{26}$$

where $3 \to 4$ utilizes the hermiticity of the Hamiltonian $\hat{H}$, $4 \to 5$ adopts the equality $\nabla_{\boldsymbol{\theta}} \log \Psi = \frac{\nabla_{\boldsymbol{\theta}} \Psi}{\Psi}$ and $5 \to 6$ introduces the local energy $\mathcal{E}_{\text{loc}} = \frac{H \Psi_{\boldsymbol{\theta}}}{\Psi_{\boldsymbol{\theta}}}$. For better readability we use $\Psi_{\boldsymbol{\theta}}$ as shorthand for $\Psi_{\boldsymbol{\theta}}(\mathbf{x}_{1:E} \mid \mathbf{M})$ and integrals are taken over the full domain of the wave function.

The generalization of the energy gradient to the transferable setting is straightforward. With the loss function being an expectation over contributions of molecular configurations $\mathbf{M}$, the gradient becomes

$$\nabla_{\boldsymbol{\theta}} \mathbb{E}_{\mathbf{M}} \langle \hat{H}^{\mathbf{M}} \rangle = 2 \mathbb{E}_{\mathbf{M}} \mathbb{E}_{\mathbf{r} \sim \Psi_{\boldsymbol{\theta}}^2} \left[\left(\mathcal{E}_{\text{loc}}^{\mathbf{M}} - \mathcal{E}^{\mathbf{M}} \right) \nabla_{\boldsymbol{\theta}} \log \Psi_{\boldsymbol{\theta}}(\mathbf{x}_{1:E} \mid \mathbf{M}) \right], \tag{27}$$

where $\hat{H}^{\mathbf{M}}$ refers to the Hamiltonian implementing the nuclear potential of the molecule $\mathbf{M}$, $\mathcal{E}^{\mathbf{M}}$ is the corresponding energy expectation value, $\mathcal{E}_{\text{loc}}^{\mathbf{M}}$ is the local energy for that molecular configuration and the wave function is a function of (a varying number of) electrons with a parametric dependence on the molecular configuration.

5.3 Multiple determinants

Departing from almost all existing work in the area [4, 8, 14, 23, 24], we do not pretrain Orbformer towards a Hartree–Fock wave function. We found that the Hartree–Fock solution can be very far from the correct wave function for molecules that are not near to their equilibrium geometry (e.g. bond breaking). Creating a Hartree–Fock pretraining mechanisms for a transferable ansatz is also significantly more burdensome than using it for single molecule training.

However, when training from a random initialization, we encountered the problem that a single determinant rapidly comes to dominate the wave function, leading to an effective single determinant model. To overcome this, we first initialize all determinants to be equal as outlined in Supplementary Note 3.4, but we found that this alone was not sufficient to prevent determinant collapse. The relative weighting of determinants can be described by the probability distribution

$$\pi_{\text{det},d} = \frac{\det[A_{\boldsymbol{\theta}}^d]}{\sum_{d'} \det[A_{\boldsymbol{\theta}}^{d'}]}. \tag{28}$$

To encourage all determinants to play a role in the wave function, we introduced the following regularization term to the training objective

$$\mathcal{L}_{\text{det}} = -\lambda_{\text{det}} \mathbb{E} \left[\sum_d \log \pi_{\text{det},d}(\mathbf{x}_{1:E} \mid \mathbf{M}) \right] \tag{29}$$

where the expectation is with respect to the training data, as in (18). This regularization term is minimized when all determinants contribute equally to the wave function. Its gradient can be computed directly by automatic differentiation.

Surprisingly, we discovered that *without Hartree–Fock pretraining* but using this multi-determinant penalty and an equal-determinant initialization we could obtain the same energy as was obtained when using Psiformer with Hartree–Fock pretraining.

5.4 Ablation study on determinant collapse and Hartree–Fock pretraining

For this study, we trained from scratch on the CH_4 molecule. We used an electron batch size of 4096 split across 4 GPUs. When Hartree–Fock pretraining was used, we ran 20k steps using the same settings as outlined in the original work introducing the Psiformer architecture [14]. When the multi-determinant penalty was applied, we used the regularization weight $\lambda_{\text{det}} = 0.001$. In Supplementary Fig. 5, we see that training Psiformer without Hartree–Fock pretraining is equivalent to training a single determinant model (with or without pretraining). It appears, therefore, that the benefit of Hartree–Fock pretraining has little to do with the quality of Hartree–Fock itself, but arises simply from preventing determinant collapse.

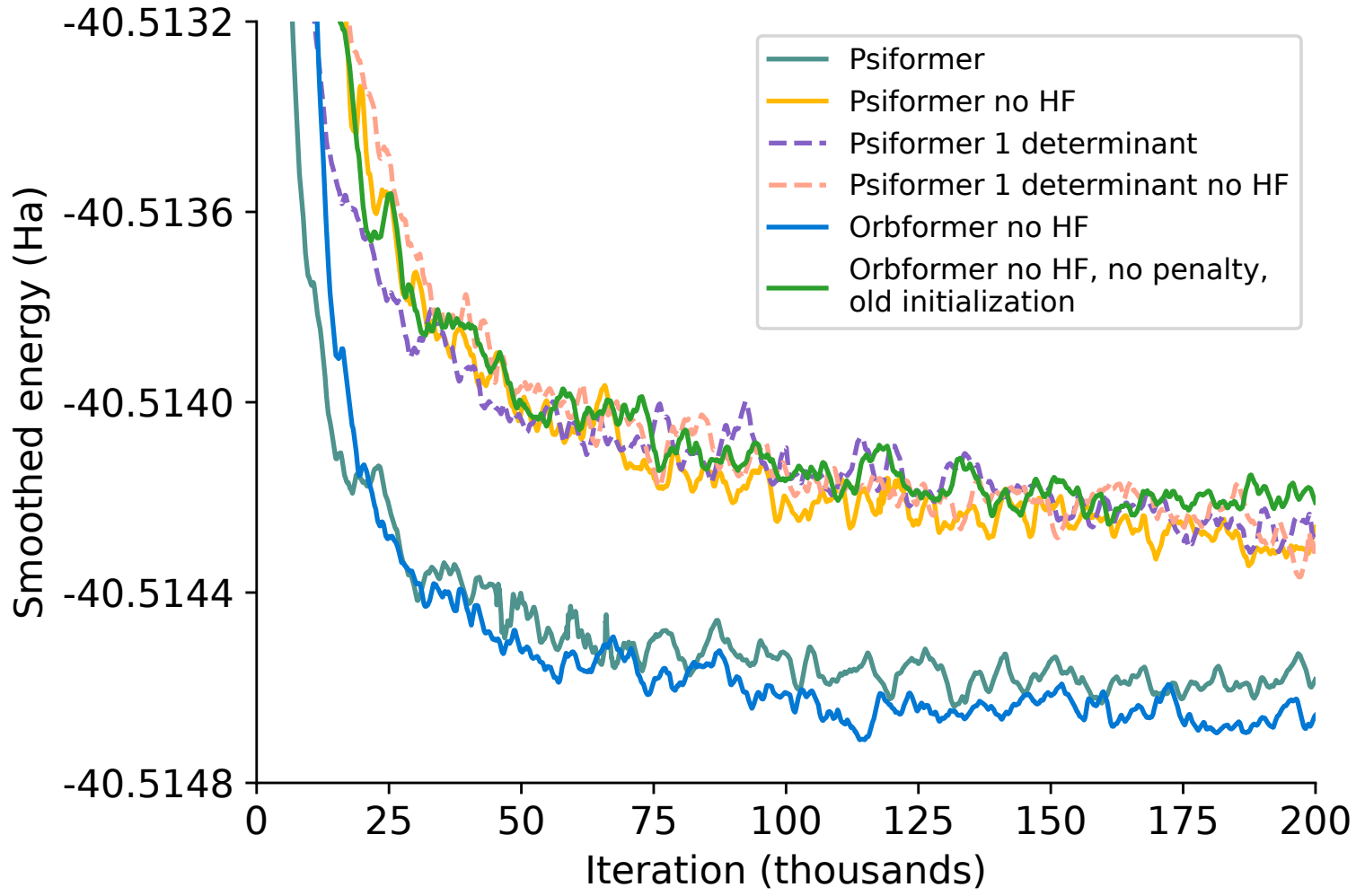

Supplementary Figure 5 | Determinant collapse. We study the connection between Hartree–Fock pretraining and determinant collapse on the CH_4 molecule at equilibrium geometry. We compare standard Psiformer training to variants with a single determinant or without Hartree–Fock (HF) pretraining. Orbformer is trained from scratch without Hartree–Fock but uses our multi-determinant penalty and initialization. Source data are provided as a Source Data file.

5.5 Optimization with K-FAC

The ansatz parameters $\boldsymbol{\theta}$ are updated to minimize the combined loss $\mathcal{L}_{\text{energy}} + \mathcal{L}_{\text{det}}$ using the K-FAC optimizer [46] implemented in the `kfac-jax` package [47]. Computing the gradient of $\mathcal{L}_{\text{energy}}$ with respect to $\boldsymbol{\theta}$ is analogous to the single molecule case as shown in Supplementary Note 5.2. We explored several alternatives to K-FAC but were unable to find one that matched its stability and speed of training.

We also apply clipping to the local energies, as this has shown itself to be essential in earlier VMC work with neural networks [4, 5, 14]. We apply clipping separately to local energies from different molecules. Furthermore, for multi-molecule training, we observe that local energies may be in very different scales for different molecules. During chemical pretraining, we therefore reweight the local energies in a similar manner to Globe [24] by computing the molecule-conditional local energy standard deviation, $s(\mathbf{M})$, and rescaling the local energies for that molecule by $\min(2, 1/s(\mathbf{M}))$; additionally we scale up the gradient of $\mathcal{L}_{\text{det}}$ by a factor of $s(\mathbf{M})$.

5.6 Tables of settings

Training settings are shown in Supplementary Table 3.

Supplementary Table 3 | Hyperparameter values used for the Orbformer training and sampling.

Component	Parameter	Value
Local energy	Clipping width	5.0
Multi-determinant loss	Regularization weight λ_{det}	0.001
Optimizer (K-FAC)	Damping	0.001
	Learning rate	0.05
	Norm constraint	0.001
Sampling	ULA steps at re-equilibration	150
	ULA initial step size	0.15
	ULA final step size	0.005
	MALA steps at re-equilibration	150
	MALA target acceptance rate	57%
	Decorrelation steps	60
	Langevin gradient norm clipping	5.0
	Pruning width	4.0

Suppl. Note 6. Efficient wavefunction Laplacians

The widespread success of foundation models is due in large part to the outstanding computational efficiency with which they can be trained on increasingly larger datasets. For chemically transferable VMC to even approach the scaling behaviour of foundation models, reducing the compute time required to evaluate the Laplacian is essential.

6.1 Computing the Laplacian in a single forward pass

The Forward Laplacian framework is a new approach to computing the Laplacian of a neural network which can result in VMC training that is significantly faster than a naive implementation [9]. The essential principle of the Forward Laplacian is encapsulated in a chain rule for the Laplacian

$$\frac{\partial^2 v}{\partial x^2} = \frac{\partial v}{\partial u}\frac{\partial^2 u}{\partial x^2} + \frac{\partial u}{\partial x}\frac{\partial^2 v}{\partial u^2}\frac{\partial u}{\partial x}. \tag{30}$$

where $v = v(u(x))$. The Forward Laplacian framework propagates the value, gradient and Laplacian forwards through the computational graph of a neural network. For further details, see Li et al. [9]. We use the Forward Laplacian implementation in the `folx` package [48].

6.2 Forward Laplacian of edge-biased attention

For most layers of Orbformer, we were able to rely on standard Forward Laplacian update rules implemented in `folx`. However, we early on identified the electron–electron attention layers (cf. Supplementary Note 3.3) as some of the most demanding computations in our model. Our use of a variant of FlashAttention [33] accelerated the forward and backward passes through the attention layers. However, no existing FlashAttention implementation exists for the Laplacian.

We therefore introduce a FlashAttention-inspired algorithm that computes the forward Laplacian update rule of the attention layers. This was possible by making use of the `pallas` kernel language in JAX [34]. The resulting kernel evaluates the Laplacian update rule for multi-head attention significantly faster than a naïve Forward Laplacian implementation applied to an un-optimized attention layer. Our kernel is publicly accessible in the `folx` package, while full details of the update rule are given in the following.

We parallelize all computations over the batch and head dimensions of the attention computation, distributing these to separate streaming multiprocessors. Here, we therefore deal only with the attention algorithm for a single head and without any batch dimensions.

6.2.1 Forward pass

We denote the number of electrons as E and the feature dimension as F. The forward pass of edge-biased attention takes as input: queries $\boldsymbol{\mathcal{Q}} \in \mathbb{R}^{E\times F}$, keys $\boldsymbol{\mathcal{K}} \in \mathbb{R}^{E\times F}$, values $\boldsymbol{\mathcal{V}} \in \mathbb{R}^{E\times F}$, and an edge bias $\boldsymbol{\mathcal{B}} \in \mathbb{R}^{E\times E}$ and computes the output $\boldsymbol{\mathcal{O}} \in \mathbb{R}^{E\times F}$ as follows

$$\mathcal{S}_{qk} = \sum_f \mathcal{Q}_{qf}\mathcal{K}_{kf} + \mathcal{B}_{qk}, \tag{31}$$

$$\mathcal{P}_{qk} = \frac{\exp(\mathcal{S}_{qk})}{\sum_{k'}\exp(\mathcal{S}_{qk'})}, \tag{32}$$

$$\mathcal{O}_{qf} = \sum_k \mathcal{P}_{qk}\mathcal{V}_{kf}. \tag{33}$$

6.2.2 Forward gradient

We use the notation $\dot{x}$ to represent the gradient of any tensor with respect to a scalar input. Our forward gradient operates in parallel over the $3E$ coordinates of the electron inputs.

$$\dot{\mathcal{S}}_{qk} = \sum_f \dot{\mathcal{Q}}_{qf}\mathcal{K}_{kf} + \sum_f \mathcal{Q}_{qf}\dot{\mathcal{K}}_{kf} + \dot{\mathcal{B}}_{qk}, \tag{34}$$

$$\dot{\mathcal{P}}_{qk} = \sum_{k'}\frac{\partial \mathcal{P}_{qk}}{\partial \mathcal{S}_{qk'}}\dot{\mathcal{S}}_{qk'} = \sum_{k'}\mathcal{P}_{qk'}(\delta_{kk'} - \mathcal{P}_{qk})\dot{\mathcal{S}}_{qk'} = \mathcal{P}_{qk}\left(\dot{\mathcal{S}}_{qk} - \sum_{k'}\mathcal{P}_{qk'}\dot{\mathcal{S}}_{qk'}\right), \tag{35}$$

$$\dot{\mathcal{O}}_{qf} = \sum_k \dot{\mathcal{P}}_{qk}\mathcal{V}_{kf} + \sum_k \mathcal{P}_{qk}\dot{\mathcal{V}}_{kf}. \tag{36}$$

6.2.3 Forward Laplacian

The second derivatives $\ddot{x}$ can be calculated similarly

$$\ddot{\mathcal{S}}_{qk} = \sum_f \ddot{\mathcal{Q}}_{qf}\mathcal{K}_{kf} + \sum_f \mathcal{Q}_{qf}\ddot{\mathcal{K}}_{kf} + \ddot{\mathcal{B}}_{qk} \qquad + 2\sum_f \dot{\mathcal{Q}}_{qf}\dot{\mathcal{K}}_{kf}, \tag{37}$$

$$\ddot{\mathcal{P}}_{qk} = \mathcal{P}_{qk}\left(\ddot{\mathcal{S}}_{qk} - \sum_{k'} \mathcal{P}_{qk'}\ddot{\mathcal{S}}_{qk'}\right) \qquad + \dot{\mathcal{P}}_{qk}\left(\dot{\mathcal{S}}_{qk} - \sum_{k'} \mathcal{P}_{qk'}\dot{\mathcal{S}}_{qk'}\right) - \mathcal{P}_{qk}\sum_{k'} \dot{\mathcal{P}}_{qk'}\dot{\mathcal{S}}_{qk'}, \tag{38}$$

$$\ddot{\mathcal{O}}_{qf} = \sum_k \ddot{\mathcal{P}}_{qk}\mathcal{V}_{kf} + \sum_k \mathcal{P}_{qk}\ddot{\mathcal{V}}_{kf} \qquad + 2\sum_k \dot{\mathcal{P}}_{qk}\dot{\mathcal{V}}_{kf}. \tag{39}$$

The Laplacian is then defined as the sum of second derivatives with respect to every electron coordinate

$$\Delta x = \sum_{e=1}^{E}\sum_{i=1}^{3} \frac{\partial^2 x}{\partial r_{ei}^2}. \tag{40}$$

In our algorithm, we separate the parts of the Laplacian that can be computed directly from $\Delta\mathcal{Q}, \Delta\mathcal{K}, \Delta\mathcal{V}, \Delta\mathcal{B}$ from the parts of the Laplacian involving some terms from the Jacobian.

To accelerate this algorithm using `pallas`, we note that every operation of the Forward Laplacian update is fully parallel over the query axis (indices labelled as q). This means that we can break inputs into blocks along the q-axis and distribute them to separate streaming multiprocessors. Thus, intermediate tensors such as $\boldsymbol{\mathcal{S}}$, $\boldsymbol{\mathcal{P}}$ and their derivatives are not materialized explicitly, leading to significant performance improvements.

We note that we have not explored writing a `pallas` kernel for the cross-attention mechanism that was introduced by Li et al. [9], this has the potential to be even faster than our current set-up, whilst deviating further from standard transformers.

6.2.4 Backward gradient

We also implemented a simplified version of the backward gradient that is found in FlashAttention. The exact calculations, for a terminal scalar loss denoted $\mathcal{L}$, are

$$\frac{\partial\mathcal{L}}{\partial\mathcal{V}_{ka}} = \sum_q \mathcal{P}_{qk}\frac{\partial\mathcal{L}}{\partial\mathcal{O}_{qa}}, \tag{41}$$

$$\frac{\partial\mathcal{L}}{\partial\mathcal{P}_{qk}} = \sum_a \mathcal{V}_{ka}\frac{\partial\mathcal{L}}{\partial\mathcal{O}_{qa}}, \tag{42}$$

$$\frac{\partial\mathcal{L}}{\partial\mathcal{S}_{qk}} = \sum_{k'} \frac{\partial\mathcal{L}}{\partial\mathcal{P}_{qk'}}\frac{\partial\mathcal{P}_{qk'}}{\partial\mathcal{S}_{qk}} \tag{43}$$

$$= \sum_a \frac{\partial\mathcal{L}}{\partial\mathcal{O}_{qa}}\mathcal{V}_{ka}\mathcal{P}_{qk} - \sum_a \frac{\partial\mathcal{L}}{\partial\mathcal{O}_{qa}}\mathcal{O}_{qa}\mathcal{P}_{qk}, \tag{44}$$

$$\frac{\partial\mathcal{L}}{\partial\mathcal{B}_{qk}} = \frac{\partial\mathcal{L}}{\partial\mathcal{S}_{qk}}, \tag{45}$$

$$\frac{\partial\mathcal{L}}{\partial\mathcal{Q}_{qa}} = \sum_k \frac{\partial\mathcal{L}}{\partial\mathcal{S}_{qk}}\mathcal{K}_{ka}, \tag{46}$$

$$\frac{\partial\mathcal{L}}{\partial\mathcal{K}_{ka}} = \sum_q \frac{\partial\mathcal{L}}{\partial\mathcal{S}_{qk}}\mathcal{Q}_{qa}. \tag{47}$$

For implementation, the primary difference here is that we *cannot* parallelize the backward pass over the query axis for every input, because we require a summation over this axes for $\partial\mathcal{L}/\partial\mathcal{V}$ and $\partial\mathcal{L}/\partial\mathcal{K}$. For the backward gradient of $\boldsymbol{\mathcal{Q}}$, we parallelize over the query axis and run an inner summation over the key axis (label k). For the backward gradient of $\boldsymbol{\mathcal{K}}, \boldsymbol{\mathcal{V}}$, we parallelize over the key axis and run an inner summation over the query axis. The backward gradient of $\boldsymbol{\mathcal{B}}$ is parallel over both axes.

Suppl. Note 7. Large scale chemical pretraining

7.1 Curriculum learning for chemical space

Our objective is to pretrain a transferable wave function ansatz across a region of chemical space defined by a distribution over molecular structures, p_{train}.

Size-based curriculum. It is well known that *gradient steps are much cheaper for smaller molecules*. The computation of the Slater determinants, for instance, scales as $O(E^3)$ in the number of electrons. The design of Orbformer outlined in Supplementary Note 3 placed significant emphasis on 'composability'. We expect that enforcing composability in our model will allow us to learn generalizable features on small molecules that will transfer to larger ones. Given this, it seems sensible to take as many optimization steps on the smallest possible molecules, where they are cheapest, before training on larger molecules. This led us to use a curriculum learning [49] approach where we train Orbformer on datasets of increasingly large molecules. We created two nested curriculum levels with up to 10 and 24 electrons respectively.

Choice of atomic species and molecules. We train Orbformer on a dataset containing the atomic species H, Li, B, C, N, O, and F. We first selected H, C, N, O, and F as a basic set of atoms that together define a rich chemical space including a significant fraction of all organic molecules [50]. We also elected to include Li and B since they are light atoms that allow us to cover varied and well-studied chemistry beyond standard organic chemistry [51, 52]. This choice of species allows us to convincingly demonstrate wave function generalization across chemical space and allows us to explore a variety of interesting applications, whilst keeping the number of electrons (and hence training cost) to a minimum. Our pretrained Orbformer is limited to operate on this set of light atoms and is not expected to work on other atom types.

Given this set of atoms, we identified a rich set of molecules of up to 24 electrons that contain any of these species. We sought to include all common organic groups [50] as well as commonly studied Li- and B-containing molecules [51, 52]. We restrict ourselves to molecules with no overall charge and with a spin state that is $S_z = 0$ or $S_z = \frac{1}{2}$, as discussed in Supplementary Note 3.1. Several molecules were set aside as an out-of-distribution test set. Supplementary Table 4 lists all molecules selected.

Geometry sampling. As discussed in Sec. 1, the most promising application area for a chemically transferable wave function model is to molecular structures with 'multireference character'. It is calculations on these structures where the absence of a *de facto* standard method from classical quantum chemistry [53, 54] leaves a space for neural network VMC to fill. We expect that geometries that are highly perturbed relative to the equilibrium geometry of a molecule, e.g. by bond breaking, are more likely to exhibit multireference character (we shortly confirm this for our own geometries). Yet the majority of existing work using neural network ansätze for molecules has focused on equilibrium or near-equilibrium geometries [4, 5, 14, 23]. We also expect that generalization is unlikely to occur from training on a few geometries per molecule—a significantly larger number of structures than has previously been considered is likely needed before the benefits of generalization will manifest.

We therefore generate a large number of perturbed geometries programmatically. Our starting point is the Z-matrix [55] of the equilibrium geometry or a known stereoisomer. We apply probabilistic distortion to the angles and bond lengths of the Z-matrix to create new off-equilibrium geometries. When simulating bond-breaking, we allow a single distance entry of the Z-matrix to be distorted by a much larger degree than the others. Finally, we apply rejection sampling [56] to prevent collision between any pair of atoms. For each molecule, we generate 100–300 geometries using the five sampling procedures outlined in Supplementary Note 9.1. In addition, we generate a number of hypothetical reaction mechanisms for small molecules (see Supplementary Table 4).

The resulting Light Atom Curriculum (LAC) contains 22350 structures; its molecular composition along with the proportion of potentially multireference structures are detailed in Supplementary Note 9.1.

We divide LAC into two curriculum levels. Structures with up to 10 electrons with bend, stretch, and break geometries are included in LAC Level 1. The full dataset is referred to as Level 2 and contains all of Level 1.

Finally, we apply on-the-fly data augmentation to all of our structures by (i) applying a random rotation, (ii) adding independent Gaussian noise of standard deviation 0.1 Bohr to every atom coordinate.

7.2 Infrastructure and training phases

We trained Orbformer using up to 16 NVIDIA A100 PCIe GPUs. Thanks to improvements in model stability, we were able to use TensorFloat-32 [57] precision for matrix multiplication during training. We conducted three sequential phases of pretraining. We found that further dividing LAC Level 1 training into two separate phases, the first using only bend and stretch geometries, led to more stable training. During Level 1 training, we used 8 GPUs with one molecule per GPU; for Level 2 training we increased to 16 GPUs. If we encountered a NaN value during training, we wound the model back to the last checkpoint, drew a new batch of training molecules, and re-equilibrated electron samples to continue training. We allowed up to 200 restarts per phase.

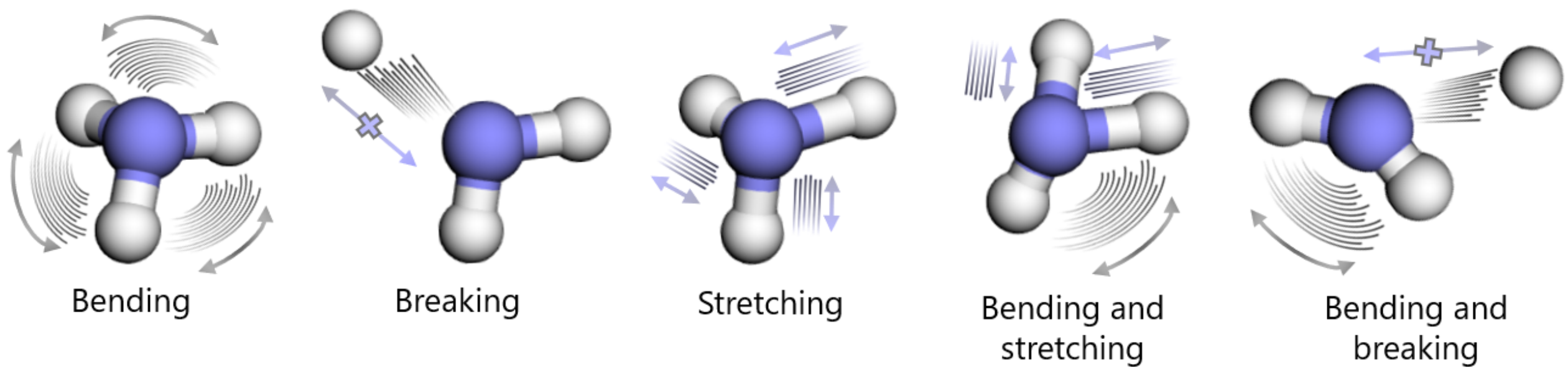

Supplementary Figure 6 | Geometry sampling methods used for programmatic generation of the LAC dataset. We generated 50 structures using bending, breaking, stretching and bending-with-stretching for every molecule, and 100 structures using bending-with-breaking. Note that rotation is applied separately as a data augmentation.

Suppl. Note 8. Fine-tuning and Evaluation

8.1 Transferable fine-tuning

We found that it was efficient to fine-tune similar structures together using a single set of parameters and simultaneous training. This allows us to use the transferable nature of Orbformer twice—once to pretrain the model, and again to share compute when fine-tuning. For example, we fine-tune different geometric structures of the same molecule together, and for the Diels–Alder reaction, we fine-tuned reactants, products, and transition states together. This can often lead to an order of magnitude reduction in cost over and above the benefits of pretraining.

We recommend always fine-tuning Orbformer on target structures, as we found that at least a few steps were always necessary. Even with no fine-tuning, energy evaluation of the Orbformer wave function requires running MCMC and computing local energies on target structures. Thus, in practical terms, if the amount of fine-tuning is of the same order of magnitude as the number of evaluation steps, fine-tuning does not significantly increase the cost of using Orbformer.

8.2 Energy evaluation

To correctly assess the energy expectation value of the wave function, we re-equilibrate new MCMC chains after the fine-tuning and compute the local energy over many MCMC steps. The final energy estimate should be the average over the chains and time steps, however, we found in practice that a few extreme values could lead to significant deterioration in the estimates of the mean. We therefore estimate a *robust mean* of the evaluation data by minimizing the Huber loss [58] over the set of evaluated local energies

$$\ell_\delta(x) := \begin{cases} \frac{1}{2}x^2 & \text{if } |x| < \delta \\ \delta(|x| - \frac{1}{2}\delta) & \text{otherwise,} \end{cases} \tag{48}$$

$$\mathcal{E}_{\text{robust}} = \min_x \sum_b \sum_t \ell_\delta(x - \mathcal{E}_{bt}) \tag{49}$$

where b and t respectively indicate the batch of MCMC samplers and the time step (with suitable decorrelation). The variance between energy estimates from different chains gives an estimate of the statistical error in our energy estimate.

8.3 Metrics

The primary metric we use to compare Orbformer energies to a reference is mean absolute relative error (MARE). Suppose we have a reaction curve with geometries indexed by $i = 1, \dots, n$. Suppose that we have corresponding energy curves $(E_i)_{i=1}^n$ and $(E'_i)_{i=1}^n$. Let $\bar{E} = \frac{1}{n}\sum_{i=1}^n E_i$ and $\bar{E}' = \frac{1}{n}\sum_{i=1}^n E'_i$. Then the mean absolute relative error is defined by $\text{MARE}(E_1, \dots, E_n; E'_1, \dots, E'_n) = \frac{1}{n}\sum_{i=1}^n |(E_i - \bar{E}) - (E'_i - \bar{E}')|$.

Suppl. Note 9. Structure generation and dataset composition

9.1 Light Atom Curriculum dataset

Supplementary Table 4 details the composition of the LAC dataset and Supplementary Fig. 6 illustrates the basic geometry distortion types used to generate a diverse set of configurations for the molecules of Supplementary Table 4.

To assess the multireference character of our molecules, we compute T1, D1 and the percentage of the contribution of (T) to the total atomization energy (|%TAE|) diagnostics [53] using CCSD(T)/cc-pVDZ calculations via PySCF 2.7.0

Supplementary Table 4 | Composition of the Light Atom Curriculum (LAC) dataset of 22350 structures. Formulas marked with a star contribute two stereo-conformers. The excluded molecules are the test molecules from the TinyMol dataset [23].

Subset	Formula	N_{elec}	Geometries
Dimers	H_2	2	50 stretch<br>50 break
	LiH	4	
	HF	10	
	C_2	12	
	LiF	12	
	CO	14	
	N_2	14	
	NO	15	
	O_2	16	
	F_2	18	
Trimers	CH_2	8	50 bend<br>50 stretch<br>50 break<br>50 stretch & bend<br>100 break & bend
	H_2O	10	
	LiOH	12	
	CNH	14	
	HCN	14	
	Li_2O	14	
	CHF	16	
	HNO	16	
	LiCN	16	
	LiOF	20	
	FCN	22	
	N_2O	22	
	O_3	24	
4 atoms	BH_3	8	50 bend<br>50 stretch<br>50 break<br>50 stretch & bend<br>100 break & bend
	NH_3	10	
	$LiNH_2$	12	
	C_2H_2	14	
	BH_2F	16	
	CH_2O	16	
	H_2N_2*	16	
	H_2O_2*	18	
	LiCCLi	18	
	LiCHO	18	
	NH_2F	18	
	LiOOLi	22	
	BHF_2	24	
	CHOF	24	
	HNO_2*	24	
5 atoms	CH_4	10	50 bend<br>50 stretch<br>50 break<br>50 stretch & bend<br>100 break & bend
	$LiCH_3$	12	
	CH_2BH	14	
	CH_2NH	16	
	CH_3F	18	
	HBCBH	18	
	NH_2OH	18	
	$LiCH_2F$	20	
	LiNHOH	20	
	$LiONH_2$	20	
	CH_2BF	22	
	HCOOH	24	
6 atoms	$LiBH_4$	12	50 bend<br>50 stretch<br>50 break<br>50 stretch & bend<br>100 break and bend
	C_2H_4	16	
	CH_3OH*	16	
	$LiCHCH_2$	18	
	N_2H_4*	18	
	H_2BCCH	20	
	$HBCCH_2$	20	
	$LiOCH_3$	20	
	CH_2BOH	22	
	CH_2CHF	22	
	CH_3BO	22	
	CH_3CN	22	
	CH_3NO	24	
	HBO_2H_2*	24	
	$HCONH_2$	24	
7 atoms	CH_3BH_2	16	50 bend<br>50 stretch<br>50 break<br>50 stretch & bend<br>100 break & bend
	CH_3NH_2*	18	
	$LiCH_2NH_2$	20	
	CH_2BNH_2	22	
	CH_3BNH	22	
	CH_3NBH	22	
	C_2H_4O	24	
	CH_2CHOH*	24	
	CH_3CHO*	24	
8 atoms	C_2H_6*	18	50 bend, stretch, break<br>50 stretch & bend<br>100 break & bend
	$LiCH_2CH_3$	20	
	BH_2CHCH_2	22	
	CH_3BCH_2	22	
Reactions	$H_4 \longrightarrow 2H_2$	4	50 structures
	$C_2H_4 \longrightarrow C_2H_2 + H_2$	16	
	$H_2O_2 \longrightarrow H_2O + O\cdot$	18	
	$CH_3OH \longrightarrow CO + 2H_2$	18	
	$N_2H_4 \longrightarrow H_2N_2 + H_2$	18	
	$C_2H_6 \longrightarrow C_2H_4 + H_2$	18	
	$2HF \longrightarrow H_2 + F_2$	20	
H-bonding	$2H_2O$	20	50 structures
	$H_2O + NH_3$	20	100 structures
	$H_2O + HF$	20	
Excluded	C_3H_4	22	n/a
	CN_2H_2	22	
	CNOH	22	
	CO_2	22	

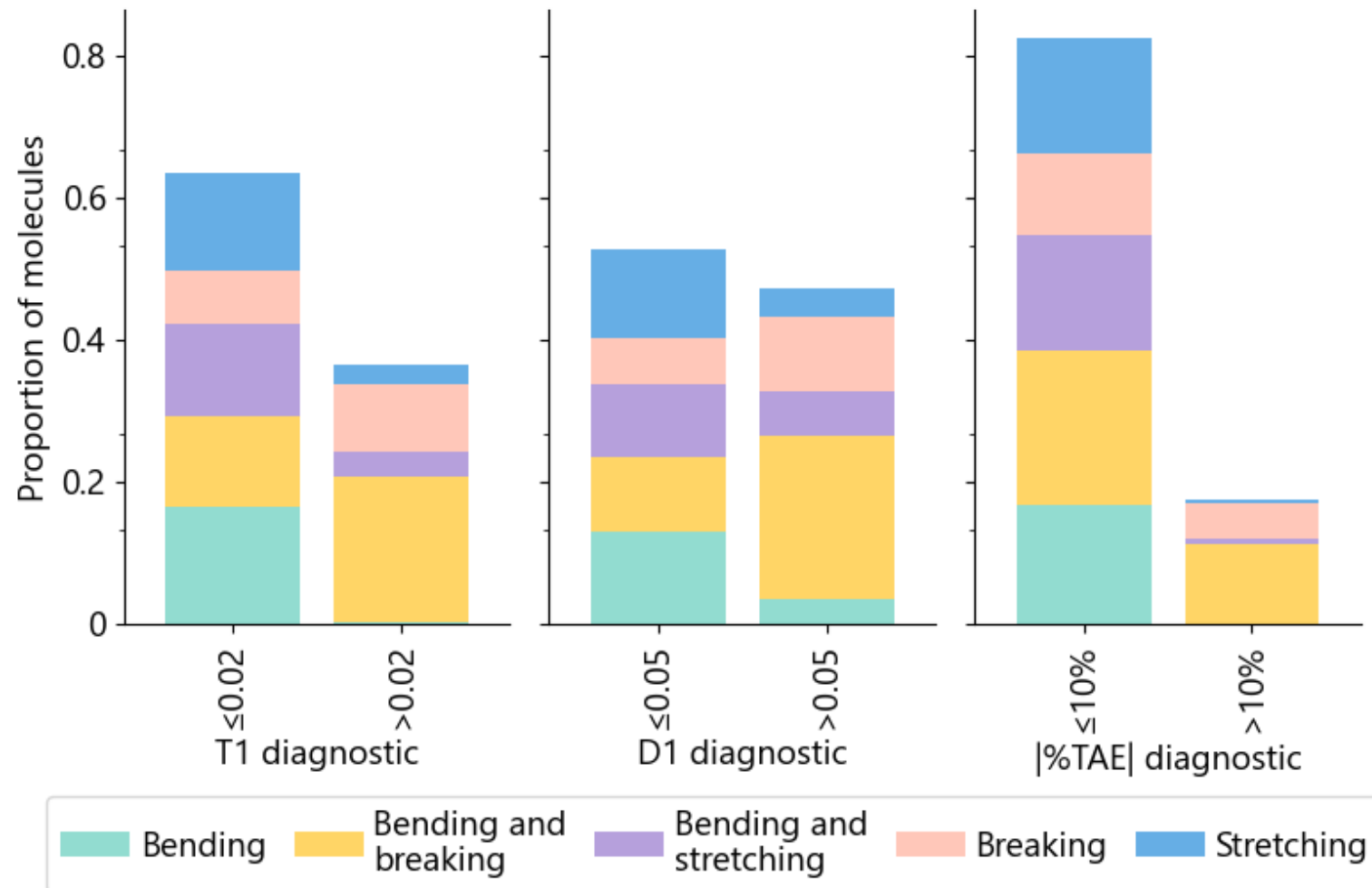

Supplementary Figure 7 | Multireference diagnostics for the LAC. The T1 D1, and |%TAE| diagnostics computed using CCSD/cc-pVDZ wave functions for all the geometries of BH_3, $LiCH_3$, C_2H_4 and NH_2F that are present in the LAC dataset. These established diagnostics indicate that LAC dataset contains a significant fraction of structures with multireference character. Source data are provided as a Source Data file.

[59] for structures of 5 selected molecules with different electron numbers in the LAC dataset (1500 structures in total). The |%TAE| diagnostic is computed using

$$|\%\text{TAE}| = \frac{|\text{TAE}[\text{CCSD(T)}] - \text{TAE}[\text{CCSD}]|}{\text{TAE}[\text{CCSD(T)}]}, \tag{50}$$

where TAE[CCSD(T)] and TAE[CCSD] are the total atomization energies computed via CCSD(T)/cc-pVDZ and CCSD/cc-pVDZ, respectively. We apply the criteria of $\text{T1} \geq 0.02$, $\text{D1} \geq 0.05$, and $|\%\text{TAE}| \geq 10\%$ to indicate if a structure might exhibit multireference character [53]. Supplementary Fig. 7 displays the proportion of structures with multireference character for each geometry distortion. There are a significant number of structures showing multireference character, particularly with geometries distorted via bond breaking. We repeated this analysis for the much smaller TinyMol dataset, finding that very few, if any, structures exhibit multireference character in that dataset.

9.2 Bond breaking MEP

We take the BSE49 [60] dataset as our inspiration when constructing our own BBMEP dataset. BSE49 is a diverse dataset that benchmarks the homolytic dissociation energies of various molecules (A−B) by computing the closed-shell molecules and their corresponding radical fragments (A· and B·). However, it remains challenging to compute structures and energies along the entire dissociation curve due to the multireference character of those structures. In this study, we select five closed-shell molecules with different electron sizes from BSE49 [60] dataset (Supplementary Table 5) and construct their corresponding minimum energy paths (MEPs), i.e., transition paths between initial and final images, of the homolytic bond breaking reactions ($\text{A}{-}\text{B} \longrightarrow \text{A}\cdot + \text{B}\cdot$), which is referred to as the BBMEP dataset. The A−B reactant structures (initial images) are directly extracted from BSE49 dataset and the product structures (final images) are constructed by combining the two radicals along its original bond direction at 6 Å. The bonds that are broken are shown in the Figure 3 in the main text. The Nudged Elastic Band (NEB) [61] method is a commonly used approach to find the MEPs for homolytic cleavage reactions, and we apply the climbing-image [62] AutoNEB [63] implementation in ASE 3.22.1 [64] with 20 total images relaxed by PBE [65] / def2-TZVP [66] with D3BJ [67, 68] corrections computed using ORCA 5.0 [69]. The initial and final images are provided to the AutoNEB and the initial guesses of the intermediate images are generated by image dependent pair potential (IDPP) [70] approach and then optimized with FIRE [71] optimizer and a maximum force of 0.05 (fmax=0.05) along the NEB path. The final collected MEP structrues are included in our GitHub repository. Please note that the endpoint structures that we use in our 20-point dissociation pathways are *not* the same as the BSE49 endpoint structures, but are produced from the AutoNEB procedure.

Deep QMC reference energies. The deep QMC reference energies that we computed for our BBMEP dissociation curves are presented in Supplementary Table 6. The protocol used to compute these values is described in Supplementary Table 8. Note that we use much larger batch sizes for the reference calculations, ran separate single-point calculations for every geometry. To reduce similarity between our own deep QMC calculations and the references, we used the Psiformer ansatz for Ethane and Formamide. However, we struggled to converge Psiformer for the larger molecules. We therefore used

Supplementary Table 5 | Composition of the bond breaking minimum energy path (BBMEP) dataset. Each path contains 20 total images, including the initial and final images.

System	Reaction	Dissociated bond	N_{elec}
Ethane	$CH_3CH_3 \longrightarrow CH_3CH_2\cdot + H\cdot$	C−H	18
Formamide	$HCONH_2 \longrightarrow HCONH\cdot + H\cdot$	N−H	24
1-Propanol	$CH_3CH_2CH_2OH \longrightarrow CH_2CH_2CH_2O\cdot + H\cdot$	O−H	34
2-Aminopropan-2-ol	$(CH_3)_2NCH_2OH \longrightarrow \cdot CH_2N(CH_3)_2 + \cdot OH$	C−O	42
L-Alanine	$HOOCCH(CH_3)NH_2 \longrightarrow HOOC\cdot + \cdot CH(CH_3)NH_2$	C−C	48

Supplementary Table 6 | Reference deep QMC absolute energies of five MEP curves in Hartree. The stochastic errors are also included.

Image ID	Ethane	Formamide	1-Propanol	2-Aminopropan-2-ol	L-Alanine
0	-79.82485(164)	-169.90316(12)	-194.35847(2)	-249.72372(12)	-323.76290(22)
1	-79.82256(55)	-169.90378(1)	-194.35793(4)	-249.72086(9)	-323.77085(26)
2	-79.81550(57)	-169.89904(10)	-194.35672(7)	-249.72220(10)	-323.76719(29)
3	-79.77022(40)	-169.88887(9)	-194.34606(10)	-249.72366(9)	-323.76160(28)
4	-79.70922(50)	-169.86811(8)	-194.31957(9)	-249.72745(5)	-323.76803(33)
5	-79.65521(5)	-169.73234(9)	-194.23575(9)	-249.72454(11)	-323.77344(10)
6	-79.63422(74)	-169.71585(4)	-194.20289(3)	-249.69502(10)	-323.75211(29)
7	-79.60235(143)	-169.83813(6)	-194.18426(4)	-249.63980(8)	-323.75864(3)
8	-79.59743(121)	-169.86235(5)	-194.17617(4)	-249.61615(12)	-323.76477(25)
9	-79.63422(41)	-169.86518(9)	-194.17345(7)	-249.59906(8)	-323.76331(23)
10	-79.64304(153)	-169.86527(6)	-194.17316(5)	-249.58461(7)	-323.75992(11)
11	-79.64785(146)	-169.85041(6)	-194.17274(5)	-249.57176(14)	-323.76476(15)
12	-79.64688(145)	-169.73853(4)	-194.17312(5)	-249.56164(13)	-323.75962(11)
13	-79.64784(161)	-169.69446(8)	-194.17262(9)	-249.56518(14)	-323.76217(5)
14	-79.64879(50)	-169.69352(4)	-194.17324(9)	-249.56474(26)	-323.66576(19)
15	-79.64920(17)	-169.69160(8)	-194.17328(7)	-249.56572(29)	-323.60473(8)
16	-79.64993(97)	-169.69411(14)	-194.17306(7)	-249.56542(8)	-323.52371(5)
17	-79.65030(78)	-169.69243(18)	-194.17617(13)	-249.56586(20)	-323.60698(9)
18	-79.65005(126)	-169.69549(12)	-194.17523(17)	-249.56580(13)	-323.60705(35)
19	-79.64967(84)	-169.69411(6)	-194.17625(3)	-249.56581(14)	-323.61342(9)

Orbformer in single-molecule mode for these reference calculations—this version of the ansatz has no Orbital Generator network making it closer in spirit to the Psiformer while being more stable.

We perform two validations on these reference energies. First, Supplementary Table 8 shows the statistical errors in the Monte Carlo evaluation of the energy that we performed for each of these calculations. All statistical errors are well below 1 mHa. Secondly, we validate our *protocol* by running it on the original BSE49 endpoints. We run our protocol in two different ways—first by running separate calculations on the two dissociated fragments, and second by running on a combined system of the two fragments separated by 1000 Å. We can then compare these values to the literature dissociation energies. Since the overall dissociation energy depends only on the endpoints, it can be computed more reliably with single-reference methods, as was done in Prasad et al. [60] In Supplementary Table 7, we see that the two deep QMC references are in excellent agreement with each other, this gives and also agree well with the literature values. With these validations, we believe that our reference energies for our BBMEP structures, including the multireference structures, are the most accurate obtainable reference values.

Energy conversion calculations. A single NVIDIA A100 GPU is 300 W and a 64 core AMD EPYC 7763 CPU is 280W, therefore the GPU/CPU power conversion factor is 300/(280/64) = 68.571.

9.3 Diels-Alder reaction dataset

We aim to characterize the two possible pathways along which the cycloaddition of ethene and trans-butadiene might proceed. The concerted and step-wise mechanisms naturally share their reactants (ethene, butadiene) and product (cyclohexene). The concerted pathway proceeds through a single transition state structure, while the step-wise mechanism involves two transitions states flanking an intermediate structure. Of the two step-wise transition states, here we only consider the first one, as it has the higher energy of the two [72], and therefore presumably determines the reaction rate along the pathway. In total, this amounts to six considered molecular structures (ethene, butadiene, cyclohexene, concerted transition state, first step-wise transition state, step-wise intermediate). The geometries of these species are taken from the

Supplementary Table 7 | Comparison of dissociation energies for reactant and product structures from literature for five BBMEP curves (kcal/mol) using literature high-quality electronic structure calculations and our deep QMC reference protocol for the BBMEP experiment with large batch sizes and long training times. Note that the underlying geometries are distinct from the geometries in our BBMEP experiment; here we use geometries from the BSE49 dataset. Source data are provided as a Source Data file.

System	Literature (RO)CBS-QB3 [60]	deep QMC reference	deep QMC reference 1000 Å
Ethane	109.29	108.80	108.80
Formamide	124.50	124.50	124.47
1-Propanol	113.00	112.33	112.44
2-Aminopropan-2-ol	98.43†	97.45	97.68
L-Alanine	90.26	89.79	89.83

† This value is reported in the BSE49 dataset "BES49_Existing_1327" but labelled as "Dimethylaminomethanol"

work of Lischka [72], where they are optimized at the MRAQCC(6,6) level of theory. Zero-point vibrational and thermal corrections for all species are also taken from the same work, computed using the B3LYP density functional and the 6-31G** basis set.

Suppl. Note 10. Hyperparameters of each experiment

The hyperparameters of each experiment presented in the main text are summarized in Supplementary Table 8.

Suppl. Note 11. Additional results

11.1 Additional results on the Diels–Alder reaction

After establishing the highly satisfactory match between Orbformer and experimental results on the iconic Diels–Alder reaction energy and concerted activation barrier, we turn our attention to the intricate step-wise mechanism, involving biradical transition states and intermediate structure. Supplementary Fig. 8 summarizes theoretical estimates for the first activation energy of this pathway. As no experimental data is available for this quantity, theoretical results can only be compared among themselves. The Orbformer result agrees best with the density functional estimates, predicting an activation energy somewhere between the M06-2X and B2-PLYP values. Compared to these CASPT2 and MRAQCC predict a significantly lower energy barrier, just as for the concerted reaction pathway. Overall, Orbformer's prediction that the concerted pathway is favoured to the step-wise one by about 10 kcal/mol is in good agreement with the currently available theoretical and experimental data [73, 74].

11.2 Comparing to experimental reference for dinitrogen dissociation

Another valuable feature of Orbformer is the capacity to simultaneously model several molecules, with very different electronic structures, without loss in accuracy. To demonstrate this property we consider the PES of the N_2 dissociation and compare (geometrically) transferable training on a dataset of 38 N_2 geometries with (chemically) transferable training on the same 38 N_2 geometries and an additional ten geometries of ethene (C_2H_4), replicating the setting of Gao & Günnemann [24]. This also gives us an opportunity to compare with one of the very few experimental references for the interior of a bond dissociation curve [75]. Both trainings used a total of 200k iterations split evenly across the datasets of 38 and 48 molecules, respectively. With N_2 forming a triple bond and ethene containing double and single bonds only, these systems show very different characteristics albeit being of similar size. Despite their dissimilarity, our results in Fig 9 show that training Orbformer with ethene as a distractor does not have a negative effect on relative energies and both runs are in perfect agreement with previous results [8]. Moreover, these results confirm that Orbformer can match very well with an experimental reference, including in the strongly correlated regime. We note that the original experimental reference has uncertainty above 1 kcal/mol. The close agreement between deep-learning QMC and r12-MR-ACPF [76] (two completely unrelated theoretical methods) gives us further confidence in the adequacy of our theoretical description. Comparing total energies of the geometrically transferable N_2 optimization and the optimization on the distractor dataset, we obtain a small increase of about 0.2 mHa. We attribute this to the reduced number of training iterations per geometry. Orbformer's capability of representing distinct molecules is all the more remarkable in the context of its generalization properties in chemically transferable pretraining.

11.3 Comparing iteration timing

We compare iteration timing between different versions of Orbformer compared to a naive Psiformer implementation. In this study, we used a batch size of 256 and ran on a single geometry of each of the molecules used in the bond-breaking

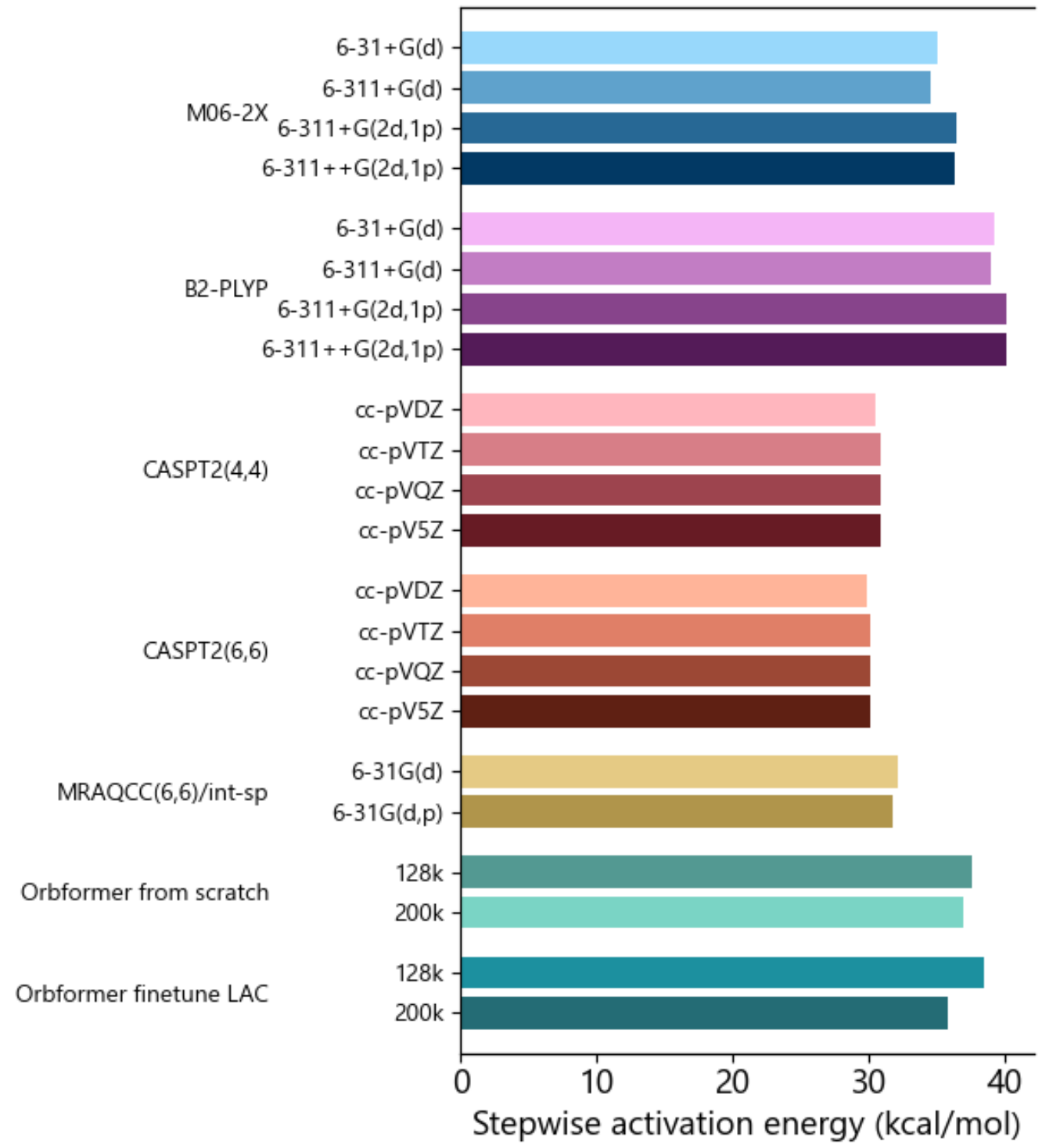

Supplementary Figure 8 | Stepwise pathway of the Diels–Alder reaction. Activation energies of the Diels–Alder reaction studied in Sec. 2.2 via the *stepwise* pathway. All details are the same as in Fig. 2. The experimental activation energy of 25.8 kcal/mol is below all results presented here, strongly indicating that the stepwise mechanism is not energetically favoured. Source data are provided as a Source Data file.

experiments. We clearly see that the larger molecules benefit more from the engineering improvements that we considered, which is consistent with Li et al. [9]. Overall, these improvements give about a 1.7× speed-up compared to a naive Psiformer and a 2.6× speed-up compared to a naive Orbformer.

11.4 Alkanes

The largest molecule in this dataset contains 106 electrons, making it significantly larger than the LAC and larger than most structures previously tackled using deep QMC. The alkanes dataset was constructed systematically with perfectly tetrahedral angles, with CC bond length of 1.5210Å and CH bond lengths of 1.0919Å. This construction ensures that, when examining composability, the local environments are identical between different alkanes.

Beyond the model introspection presented in the main paper, we also sought to establish that our model had indeed converged on this dataset including some large molecules up to 106 electrons. We fine-tuned the model across 32 GPUs with 32 electron samples per GPU. For this training, we used the ULA/MALA sampling scheme that we used for pretraining in order to get a better coverage of electron space.

Given our construction of the molecules, we would expect that the total energy scales almost exactly linearly in the number of carbon atoms. We verified that CCSD(T) indeed gives a linear energy scaling in energy for these molecules. In Supplementary Fig. 11 (left) we fitted a linear model to the total energies of the eight alkanes and saw residuals of order no more than 1 kcal/mol, with an MSE of 0.92. This indicates that the model has converged well, and furthermore it gives an indication that, with joint fine-tuning, it is not the case that smaller molecules converge faster. However, it is also clear that linear scaling of the energy is not an automatically satisfied property for Orbformer. We assume this may be because 16 determinants are used for each molecule, which hampers exact properties like size consistency and size extensivity being present. In Supplementary Fig. 11 (right), we looked at the difference between Orbformer total energies and CCSD(T)/CBS energies. Now it becomes apparent that, whilst both models achieve a linear scaling of energy with system size, the Orbformer total energies are significantly lower across the board, indicating that Orbformer does a much better job of capturing electron correlation.

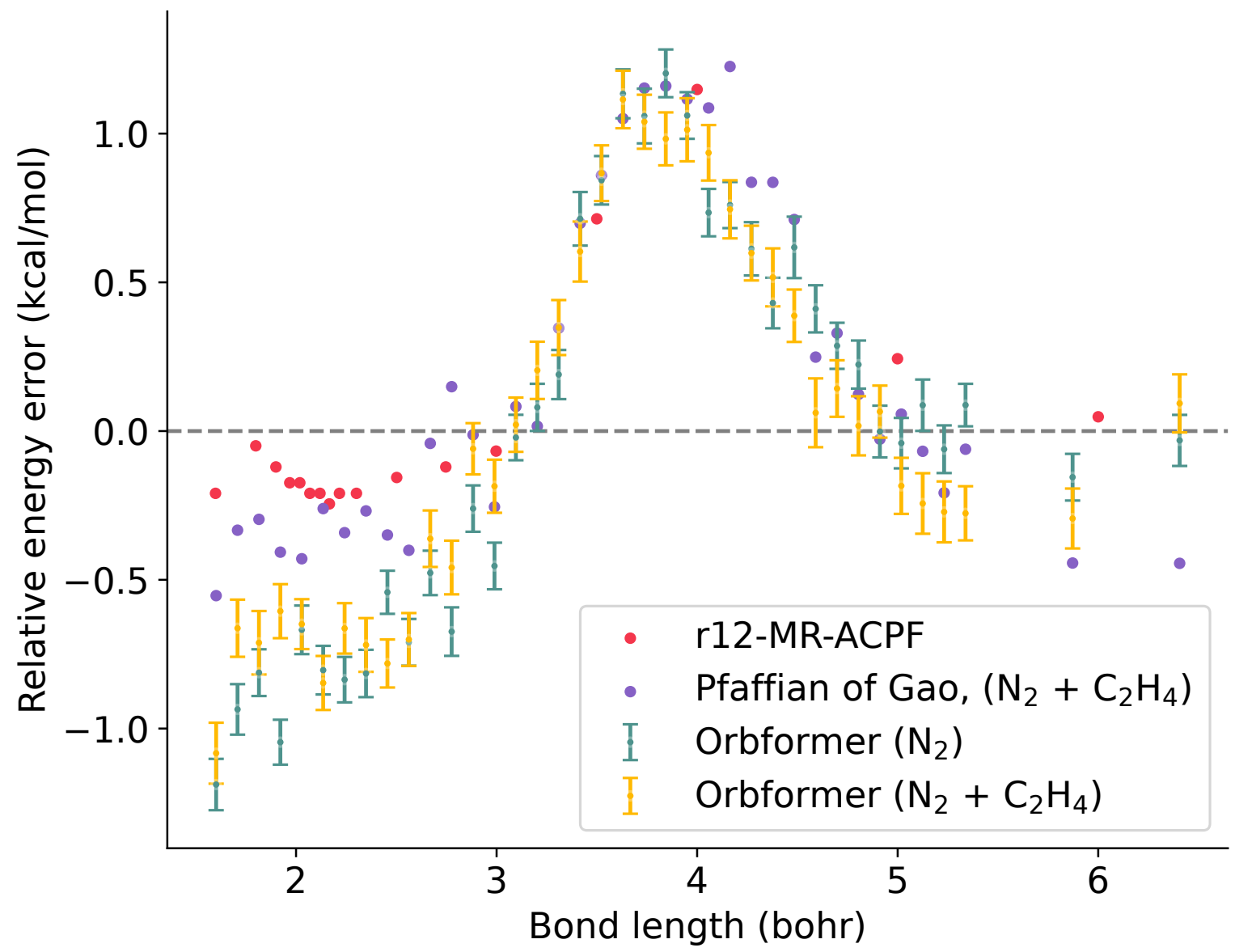

Supplementary Figure 9 | Dinitrogen dissociation with and without distractors. Relative energy of the N_2 dissociation with and without ethene as a distractor molecule in the fine-tuning dataset. Additionally, results obtained with the r12-MR-ACPF reference theoretical method are also shown [76]. The usual mean-shifting is applied to all energies. Error bars show the statistical error (one standard deviation of the estimates from independent MCMC chains, cf. Suppl. Note 8.2). Source data are provided as a Source Data file.

11.5 Azulene–naphthalene isomerization

To showcase the ability of the proposed approach to accurately describe aromaticity and large-scale conjugation, we evaluated the reaction energy of the azulene–naphthalene isomerization using an Orbformer model fine-tuned from the LAC checkpoint. Equilibrium geometries for naphthalene and azulene were obtained from Mirzaei et al. [77]. Fine-tuning was carried out jointly for the two geometries, for 80k steps. The reference reaction energy value was obtained with the W1 composite approach [78] at the CCSD(T)/CBS level of theory. Orbformer's predicted reaction energy converges convincingly within chemical accuracy of the reference 36.8 kcal/mol after 50k fine-tuning iterations. Sampling errors were not obtained, due to the use of the robust mean in computing the energy expectation values. Instead the four separate evaluations along the fine-tuning trajectory are intended to demonstrate the reliability of both sampling and training convergence. The fact that Orbformer can appropriately describe the extended delocalized π-system of naphthalene indicates that our composable architecture does not hinder modelling the behaviour of electron delocalization at this scale. In further support of this, Supplementary Fig. 13 visualizes three selected one-particle envelopes of the fine-tuned Orbformer model for naphthalene. The three orbitals, localized to one, three and four carbon atoms respectively, illustrate how Orbformer can operate on the different length-scales required for the description of the electronic structure of naphthalene. Further, we recall that Orbformer orbitals are pointwise products between envelopes and transformer representations. Transformer representations, unlike classical orbitals, are functions of all electron positions and spins rather than of one electron's position. To guarantee composability for large separations of subsystems, the distance-weighted attention mechanism employed in Orbformer introduces a finite perceptive field for the interactions. However, the electron-electron correlation is not directly mediated by the orbital envelope. This means that, even with a rather localised envelope, some long-range electron correlation can be captured by the transformer representations.

11.6 Atomization energies of small molecules

While other experiments of this manuscript mainly compare the performance of Orbformer against other theoretical methods, here we provide a comparison of Orbformer's predicted atomization energies against experimental references. Reference data was collected for 19 molecules from the active thermochemical tables (ATcT) [79], by subtracting the standard gas phase heat of formation of the molecule from those of the constituent atoms, at 0 K. Geometries and corrections for zero-point vibrational effects were obtained from the theoretical study benchmarking the W4 composite method [80]. Orbformer was finetuned from the LAC checkpoint independently for each considered molecule. The deviations of the zero-point corrected Orbformer total atomization energies (TAE) from experimental references are displayed on Supplementary Fig. 14. All but one of the Orbformer values fall within ±1 kcal/mol of the experimental value. The MAE of 0.51 kcal/mol across the 19 molecules engenders confidence in the quality of Orbformer results as compared to experiment.

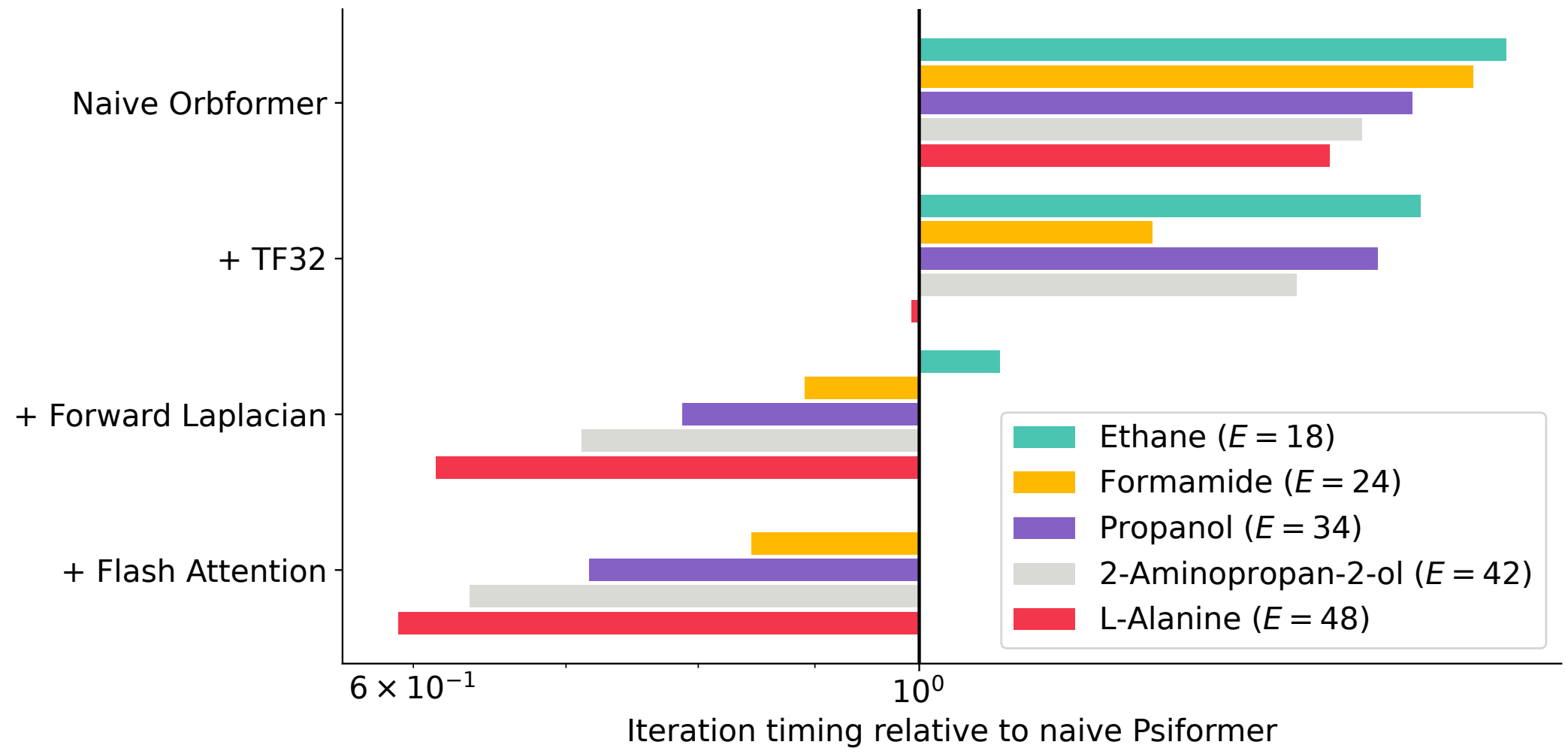

Supplementary Figure 10 | Variational training iteration time reduction from engineering enhancements. We show the relative iteration time of Orbformer compared to an unimproved Psiformer implementation, isolating the effects of TensorFloat-32 precision (TF32), Forward Laplacian and FlashAttention. Source data are provided as a Source Data file.

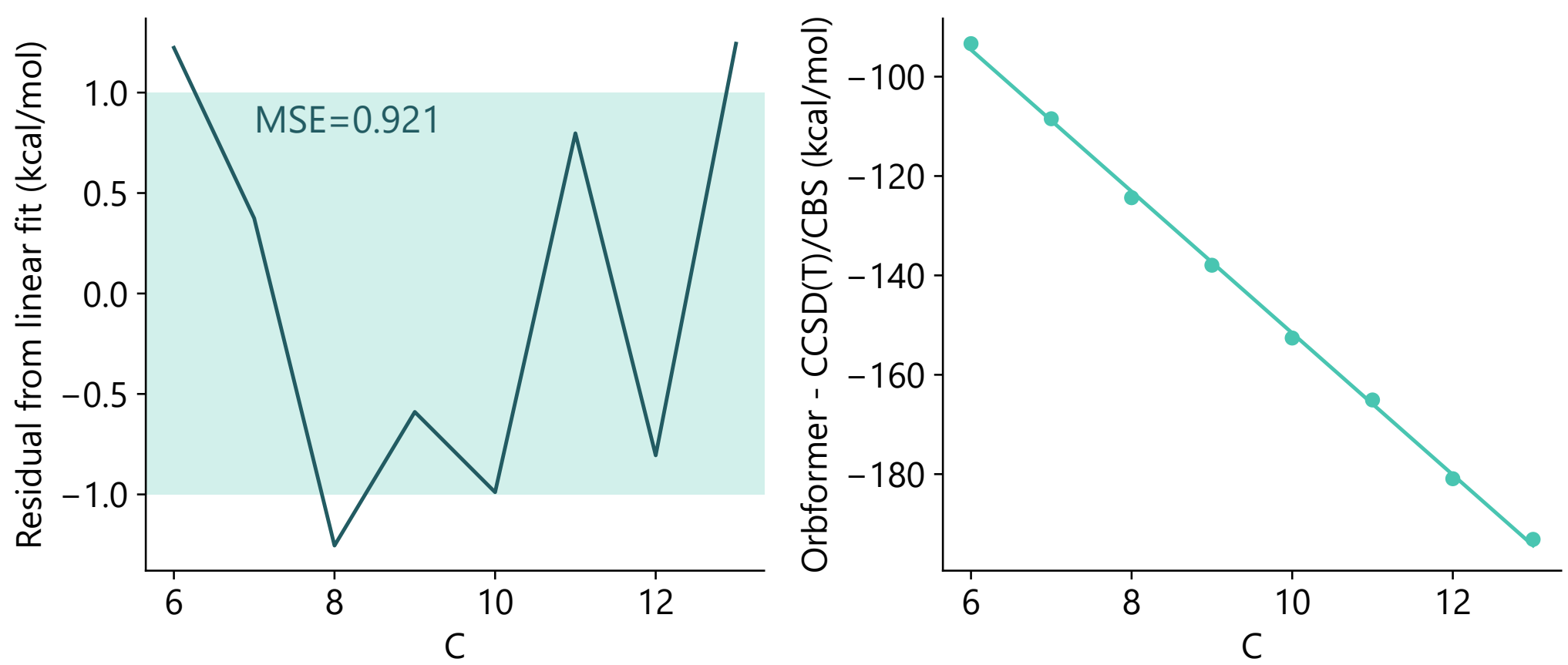

Supplementary Figure 11 | Evaluating model convergence on the alkanes dataset. We use linear scaling of energy as a criterion for convergence. Left: residuals from a linear fit of a model fine-tuned from the LAC for 180k steps. Right: the difference between the aforementioned Orbformer model and CCSD(T)/CBS total energies, with a linear fit line. Here we see that Orbformer obtains much lower energies, whilst both observe a linear scaling in the molecule size. Source data are provided as a Source Data file.

Suppl. Note 12. Traditional electronic structure calculation details

12.1 Energy calculations

To compare the efficiency and accuracy of Orbformer, we perform traditional electronic structure calculations at various costs and accuracy using commonly used theories. Without human expert design and intervention, all the calculations are run under a practitioner perspective to have a more fair comparison. Without any further specification, all the SCF calculations, including KS-DFT [81], restricted open-shell determinant (ROHF) [82] and active space SCF method (CASSCF) [83], are computed using an energy convergence threshold of 10^{-10} a.u. and gradient convergence threshold to 10^{-6} a.u. with a maximum SCF cycle of 500. All the post-SCF calculations, including unrestricted coupled-cluster with single, double and a perturbative treatment of triple excitations (UCCSD(T)) [84], the second-order n-electron valence state perturbation theory (NEVPT2) [85], and density-fitted uncontracted multireference configuration interaction with single and double excitations and Davidson size-extensivity correction (DF-MRCISD+Q) [86], are computed using an energy convergence threshold of 10^{-10} a.u. and a maximum iteration of 600.

In this study, all the complete basis set (CBS) extrapolations are performed with a two-point extrapolation strategy used in ORCA 5.0 [69] in equations 51 and 52. The final total energy is computed as $\mathcal{E}^{\infty}_{\text{total}} = \mathcal{E}^{\infty}_{\text{SCF}} + \mathcal{E}^{\infty}_{\text{corr}}$

$$\mathcal{E}^{X}_{\text{SCF}} = \mathcal{E}^{\infty}_{\text{SCF}} + A\exp(-\alpha\sqrt{X}), \tag{51}$$

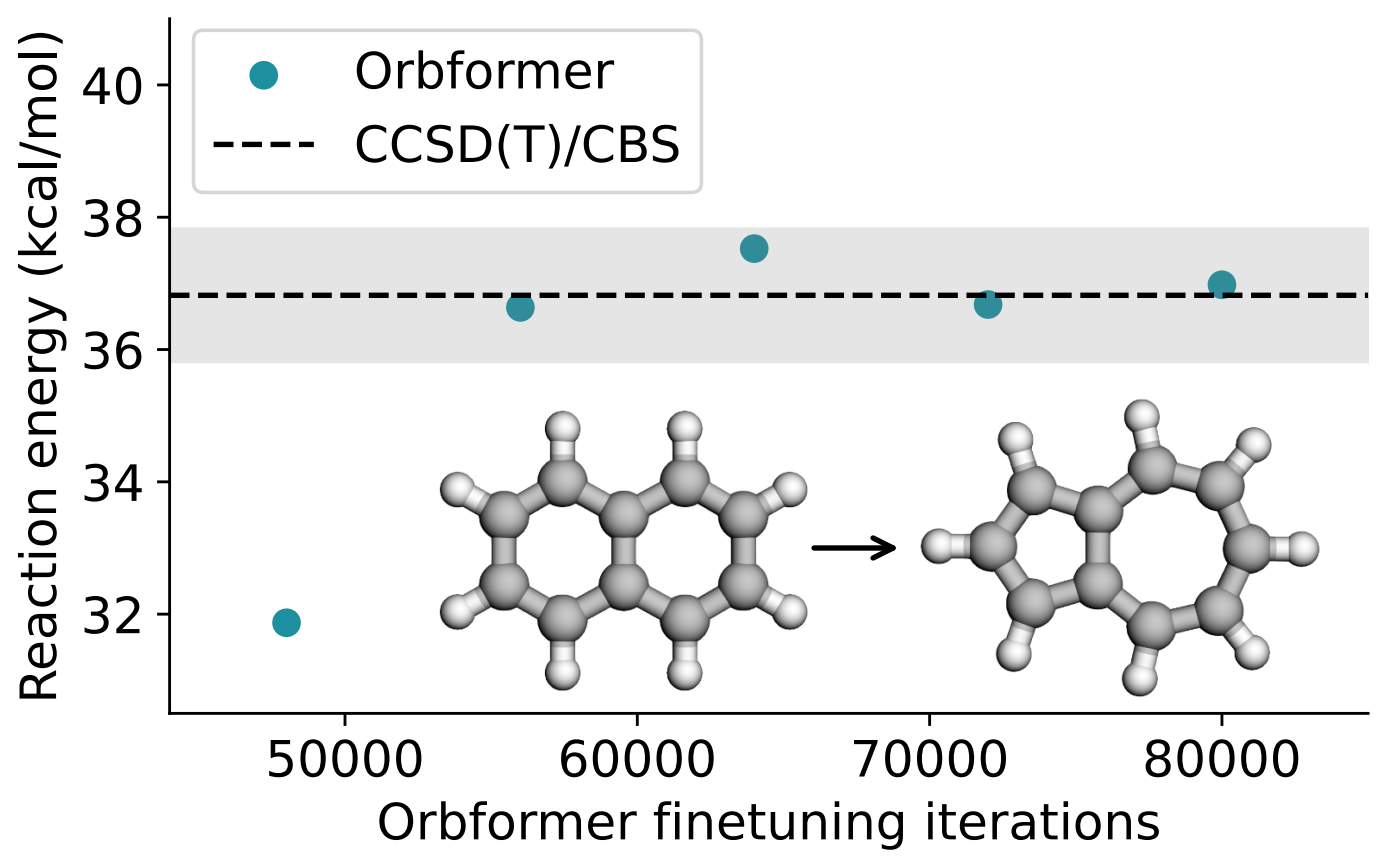

Supplementary Figure 12 | Comparison of the predicted reaction energy for the azulene-naphthalene isomerization between high-level electronic structure methods Orbformer and CCSD(T) at the complete basis set limit. The coupled cluster result was computed in-house, using the W1 thermochemical protocol [78]. The Orbformer checkpoint pretrained on LAC was finetuned on azulene and naphthalene jointly. Orbformer energies were obtained by computing the robust mean of energies obtained in independent evaluation runs. Source data are provided as a Source Data file.

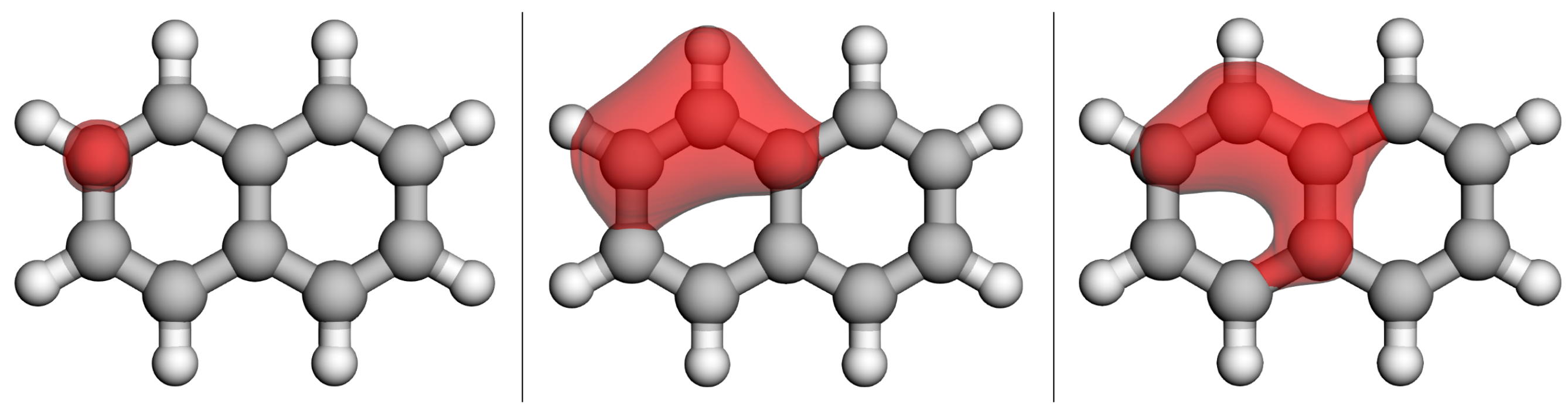

Supplementary Figure 13 | Visualizations of three selected one-particle envelopes of naphthalene from the Orbformer model finetuned on azulene and naphthalene.

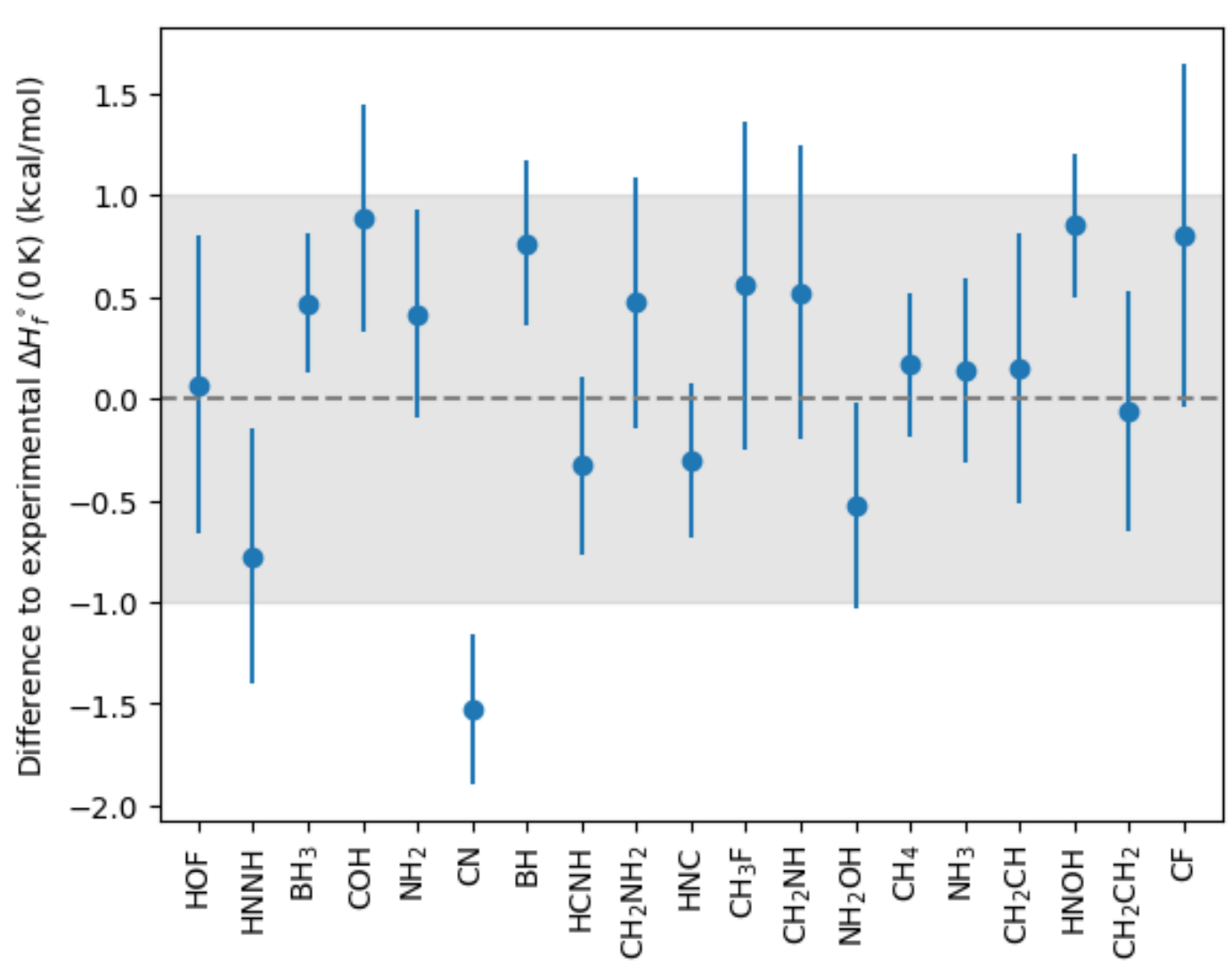

Supplementary Figure 14 | Deviations of the zero-point corrected Orbformer total atomization energies from experimental reference values [79]. Error bars show the statistical error (one standard deviation of the estimates from independent MCMC chains, cf. Suppl. Note 8.2). Source data are provided as a Source Data file.

where X is the basis set cardinal number and in this study we use aug-cc-pVDZ ($X = 2$) and aug-cc-pVTZ ($X = 3$) basis sets [87], A is a parameter to be computed, $\alpha = 4.3$ is a basis-set dependent constant [88], and $\mathcal{E}_{\mathrm{SCF}}^{\infty}$ is the corresponding SCF energy at CBS limit. We compute the extrapolated correlation energy as

$$\mathcal{E}_{\mathrm{corr}}^{\infty} = \frac{X^{\beta}\mathcal{E}_{\mathrm{corr}}^{X} - Y^{\beta}\mathcal{E}_{\mathrm{corr}}^{X}}{X^{\beta} - Y^{\beta}}, \tag{52}$$

where $\beta = 2.51$ is a basis-set dependent constant [88], $X = 2, Y = 3$ are the basis set cardinal numbers for aug-cc-pVDZ and aug-cc-pVTZ, respectively, in this study, and $\mathcal{E}_{\mathrm{corr}}^{\infty}$ is the corresponding correlation energy at CBS limit.

The computational details for each theory at specific basis sets are as follows:

- UB3LYP-D3(BJ)/Def2-QZVPP: The spin-unrestricted B3LYP[89–92] plus D3 dispersion correction with Becke–Johnson damping [67, 68] with the Def2-QZVPP basis set [93] calculations are performed by PySCF 2.7.0 [59] with PySCF-dispersion 1.3.0 plug-in, which interferences Simple-DFTD3 1.2.1 [94].

- UM06-2X-D3/Def2-QZVPP: The spin-unrestricted M06-2X [95] plus D3 dispersion correction [67, 96] with the Def2-QZVPP basis set [93] calculations are performed by PySCF 2.7.0 [59] with PySCF-dispersion 1.3.0 plug-in, which interferences Simple-DFTD3 1.2.1[94]. Note that M06-X has already included mid-range dispersion correction in the parametrization, but literature study suggests a D3 correction could still benefit the calculation [97].

- UωB97X-V-D3(BJ)/Def2-QZVPP: The spin-unrestricted ωB97X-V [98, 99] plus D3 dispersion correction with Becke–Johnson damping [67, 68] with the Def2-QZVPP basis set [93] calculations are performed by PySCF 2.7.0 [59] with PySCF-dispersion 1.3.0 plug-in, which interferences Simple-DFTD3 1.2.1 [94].

- UCCSD(T)/CBS: The UCCSD(T) with aug-cc-pVDZ and aug-cc-pVTZ basis set calculations are performed using ROHF wavefunctions [84, 100]. The two-point CBS extrapolation then is performed by separately extrapolating the SCF and correlation contributions via equations 51 and 52, respectively.

- NEVPT2/CASSCF(10,10)/CBS: In this study, NEVPT2 calculations are computed [85] using the strongly contracted (SC) internal contraction scheme [101, 102] implemented in PySCF 2.7.0 [59] with a CASSCF wavefunction. CASSCF is solved by the FCI solver [103] with 10 electron and 10 active orbitals in the active space and the initial guess of the CASSCF is using ROHF wavefuction. To automatically select an active space with a consistent active space size, we use the initial guess strategy introduced in PySCF, which is not the best but a practical approach to select active space. After ROHF calculations, we localize orbitals for occupied and virtual spaces separately via Pipek-Mezey (PM) localization [104]. The orbitals then are sorted by their MO coefficients and the top 10 MOs are picked as the active space.

- DF-MRCISD+Q/aug-cc-pVDZ: We note that due to inefficient memory for L-Alanine dissociation MEP calculations with aug-cc-pVTZ basis set, only calculations using aug-cc-pVDZ basis set were performed. All MRCISD+Q calculations are performed using frozen-core uncontracted MRCI solved by a Davidson solver implemented in ORCA 5.0 [69]. As is common practice in electronic structure calculations, we apply the density fitting (DF) approximation, also known as the resolution of identity (RI), with the automatically generated auxiliary basis set [105] to speed up the calculations. CASSCF is solved by the default solver and active space selection strategy implemented in ORCA 5.0 with 10 active electrons and 10 active orbitals in the active space and a DF-ROHF wavefuction as initial guess.

- DF-MRAQCC/aug-cc-pVDZ: Similar to the DF-MRCISD+Q/aug-cc-pVDZ calculations, we perform frozen-core uncontracted MRAQCC calculations solved by DIIS solver via ORCA 5.0 [69] with CASSCF wavefunctions initialized by ROHF solutions. Active space selection is performed by the default strategy in ORCA 5.0 an active space with 10 active electrons and 10 active orbitals.

Suppl. Note 13. Extended discussion

Looking to future developments that may follow on from Orbformer, we saw that pretraining reduces the amount of fine-tuning required to achieve chemical accuracy, often very substantially, yet in very few cases was it possible to evaluate energies directly from the pretrained model with no fine-tuning. We believe that with a further improved and much larger model than Orbformer, it will be possible to converge to chemical accuracy across a dataset as large as the LAC without the need for fine-tuning. Yet increasing model capacity without significantly slowing down training and remaining within memory constraints of current hardware presents a serious challenge. One direction would be to do more processing that is independent of the electron positions, since any computations here bypass the Laplacian. It would also be beneficial to improve the single-determinant version of the model to match the current 16-determinant Orbformer, since a single-determinant model is size consistent by construction, and this may lead to improvements in

generalization. Engineering development of the K-FAC [46] optimizer would also be necessary for further scaling of this technology. Another axis of development would be to extend Orbformer to operate on charged systems and systems with a total z-spin that is different to 0 or $\frac{1}{2}$. Here, the localization of Orbformer orbitals presents a conceptual challenge, since it is unclear where in space any orbitals should be added or removed when forming an ion. For certain molecules, say R−COO⁻, we can form a good guess as to where the negative charge will be localized. However, for a more obscure ion such as any R⁻, the new orbital may not be localized at all. The case of spin is likely to be somewhat easier to handle, as the total number of orbitals does not change. We envisage that communicating the overall spin of the molecule to all the molecule-dependent parts of the network would allow it to correctly adapt to changes in the overall molecular spin.

Supplementary Table 8 | Training hyperparameters for all the experiments. [a]Orbformer trained in single molecule mode (see Supplementary Note 3.6). [b]Trained across 32 GPUs using the ULA/MALA sampling scheme: the same molecule will be sampled on multiple GPUs but with an independent batch of electron samples.

Experiment	Dataset	Ansatz	Training mode	Electron batch size	Molecule batch size	Hartree–Fock train steps	Training steps	Evaluation steps per structure
TinyMol	TinyMol pretrain	Orbformer	chemical pretraining	1024	4	-	512 k	-
	TinyMol test sets	Orbformer	geom-transferable fine-tuning	512	4	-	32 k	8k
	TinyMol test sets	Psiformer	single-point training	2048	1	20 k	32 k	2k
BBMEP reference	Ethane	Psiformer	single-point training	2048	1	20 k	200 k	10 k
	Formamide	Psiformer	single-point training	4096	1	20 k	250 k	10 k
	1-Propanol & 2-Aminopropan-2-ol	Orbformer[a]	single-point training	4096	1	-	250 k	10 k
	L-Alanine	Orbformer[a]	single-point training	4096	1	-	300 k	10 k
BBMEP Psiformer scratch (large batch), single structures	Ethane	Psiformer	single-point training	1024	1	-	40 k	6 k
	Formamide	Psiformer	single-point training	2048	1	-	50 k	6 k
BBMEP Orbformer scratch, single structures	Ethane	Orbformer	single-point training	1024	1	-	32 k	6 k
	Formamide	Orbformer	single-point training	1024	1	-	100 k	6 k
	1-Propanol	Orbformer	single-point training	1024	1	-	120 k	6 k
	2-Aminopropan-2-ol	Orbformer	single-point training	1024	1	-	160 k	6 k
	L-Alanine	Orbformer	single-point training	1024	1	-	500 k	6 k
BBMEP Orbformer scratch or LAC fine-tuning, all structures	Ethane	Orbformer	geom-transferable training/fine-tuning	256	4	-	32 k	6 k
	Formamide	Orbformer	geom-transferable training/fine-tuning	256	4	-	100 k	6 k
	1-Propanol	Orbformer	geom-transferable training/fine-tuning	256	4	-	120 k	6 k
	2-Aminopropan-2-ol	Orbformer	geom-transferable training/fine-tuning	256	4	-	160 k	6 k
	L-Alanine	Orbformer	geom-transferable training or fine-tuning	256	4	-	500 k	6 k
Diels–Alder	all structures	Orbformer	single-point training	4096	1	-	200k	8k
	all structures	Orbformer	geom-transferable fine-tuning	512	8	-	200k	8k
Alkanes	Alkanes C6–13	Orbformer	molecule-transferable fine-tuning	32	32[b]	-	180k	20k

Supplementary References

[1] W. M. Foulkes, L. Mitas, R. Needs & G. Rajagopal. "Quantum Monte Carlo simulations of solids." *Reviews of Modern Physics* **73**, 33 (2001).

[2] R. J. Needs, M. D. Towler, N. D. Drummond & P. L. Ríos. "Continuum variational and diffusion quantum Monte Carlo calculations." *Journal of Physics: Condensed Matter* **22**, 023201 (2009).

[3] G. Carleo & M. Troyer. "Solving the quantum many-body problem with artificial neural networks." *Science* **355**, 602 (2017).

[4] J. Hermann, Z. Schätzle & F. Noé. "Deep-neural-network solution of the electronic Schrödinger equation." *Nature Chemistry* **12**, 891 (2020). DOI: https://doi.org/10.1038/s41557-020-0544-y.

[5] D. Pfau, J. S. Spencer, A. G. D. G. Matthews & W. M. C. Foulkes. "Ab initio solution of the many-electron Schrödinger equation with deep neural networks." *Phys. Rev. Res.* **2**, 033429 (3 2020). DOI: 10.1103/PhysRevResearch.2.033429.

[6] J. S. Spencer, D. Pfau, A. Botev & W. M. C. Foulkes. "Better, Faster Fermionic Neural Networks." *arXiv preprint arXiv:2011.07125* (2020). arXiv: 2011.07125.

[7] Z. Schätzle, P. B. Szabó, M. Mezera, J. Hermann & F. Noé. "DeepQMC: An open-source software suite for variational optimization of deep-learning molecular wave functions." *J. Chem. Phys.* **159**, 094108 (2023). DOI: 10.1063/5.0157512.

[8] N. Gao & S. Günnemann. "Neural Pfaffians: Solving Many Many-Electron Schrödinger Equations." *arXiv preprint arXiv:2405.14762* (2024).

[9] R. Li, H. Ye, D. Jiang, X. Wen, C. Wang, Z. Li, X. Li, D. He, J. Chen, W. Ren, et al. "A computational framework for neural network-based variational Monte Carlo with Forward Laplacian." *Nat. Mach. Intell.* **6**, 209 (2024). DOI: https://doi.org/10.1038/s42256-024-00794-x.

[10] L. Gerard, M. Scherbela, P. Marquetand & P. Grohs. "Gold-standard solutions to the Schrödinger equation using deep learning: How much physics do we need?" In *Advances in Neural Information Processing Systems* (Curran Associates, Inc., 2022). Ed. by S. Koyejo, S. Mohamed, A. Agarwal, D. Belgrave, K. Cho & A. Oh. Vol. 35, 10282–10294.

[11] J. Lin, G. Goldshlager & L. Lin. "Explicitly antisymmetrized neural network layers for variational Monte Carlo simulation." *J. Comput. Phys.* **474**, 111765 (2023). DOI: https://doi.org/10.1016/j.jcp.2022.111765.

[12] J. Kim, G. Pescia, B. Fore, J. Nys, G. Carleo, S. Gandolfi, M. Hjorth-Jensen & A. Lovato. "Neural-network quantum states for ultra-cold Fermi gases." *Commun. Phys.* **7**, 148 (2024). DOI: https://doi.org/10.1038/s42005-024-01613-w.

[13] X. Li, C. Fan, W. Ren & J. Chen. "Fermionic neural network with effective core potential." *Phys. Rev. Res.* **4**, 013021 (1 2022). DOI: 10.1103/PhysRevResearch.4.013021.

[14] I. von Glehn, J. S. Spencer & D. Pfau. "A self-attention ansatz for ab-initio quantum chemistry." *arXiv preprint arXiv:2211.13672* (2022).

[15] J. Hermann, J. Spencer, K. Choo, A. Mezzacapo, W. M. C. Foulkes, D. Pfau, G. Carleo & F. Noé. "Ab initio quantum chemistry with neural-network wavefunctions." *Nat. Rev. Chem.* **7**, 692 (2023). DOI: https://doi.org/10.1038/s41570-023-00516-8.

[16] J. A. Pople & D. L. Beveridge. "Molecular orbital theory." *C0., NY* (1970).

[17] D. A. Kleier, T. A. Halgren, J. H. Hall Jr & W. N. Lipscomb. "Localized molecular orbitals for polyatomic molecules. I. A comparison of the Edmiston-Ruedenberg and Boys localization methods." *The Journal of Chemical Physics* **61**, 3905 (1974).

[18] T. Helgaker, P. Jorgensen & J. Olsen. *Molecular electronic-structure theory* (John Wiley & Sons, 2013).

[19] P. Pulay. "Localizability of dynamic electron correlation." *Chemical physics letters* **100**, 151 (1983).

[20] A. Nakata, J. S. Baker, S. Y. Mujahed, J. T. Poulton, S. Arapan, J. Lin, Z. Raza, S. Yadav, L. Truflandier, T. Miyazaki, et al. "Large scale and linear scaling DFT with the CONQUEST code." *The Journal of chemical physics* **152** (2020).

[21] P. B. Szabó, J. Csóka, M. Kállay & P. R. Nagy. "Linear-scaling local natural orbital CCSD(T) approach for open-shell systems: Algorithms, benchmarks, and large-scale applications." *Journal of Chemical Theory and Computation* **19**, 8166 (2023).

[22] M. Hanrath. "On the concepts of connectivity, separability, and consistency: An illustration by partitioned diagrams and numerical probing." *Chemical Physics* **356**, 31 (2009).

[23] M. Scherbela, L. Gerard & P. Grohs. "Towards a transferable fermionic neural wavefunction for molecules." *Nat. Commun.* **15**, 120 (2024).

[24] N. Gao & S. Günnemann. "Generalizing neural wave functions." In *International Conference on Machine Learning* (2023). PMLR, 10708–10726.

[25] J. Bradbury et al. *JAX: composable transformations of Python+NumPy programs.* Version 0.3.13. 2018. URL: http://github.com/jax-ml/jax.

[26] C.-J. Huang, C. Filippi & C. Umrigar. "Spin contamination in quantum Monte Carlo wave functions." *The Journal of chemical physics* **108**, 8838 (1998).

[27] P. B. Szabó, Z. Schätzle, M. T. Entwistle & F. Noé. "An improved penalty-based excited-state variational Monte Carlo approach with deep-learning ansatzes." *Journal of Chemical Theory and Computation* **20**, 7922 (2024).

[28] T. Kato. "On the eigenfunctions of many-particle systems in quantum mechanics." *Commun. Pure Appl. Math.* **10**, 151 (1957). DOI: https://doi.org/10.1002/cpa.3160100201.

[29] A. Vaswani, N. Shazeer, N. Parmar, J. Uszkoreit, L. Jones, A. N. Gomez, L. Kaiser & I. Polosukhin. "Attention Is All You Need." *arXiv preprint arXiv:1706.03762* (2017).

[30] J. Lei Ba, J. R. Kiros & G. E. Hinton. "Layer normalization." *ArXiv e-prints*, arXiv (2016).

[31] Q. Wang, B. Li, T. Xiao, J. Zhu, C. Li, D. F. Wong & L. S. Chao. "Learning deep transformer models for machine translation." *arXiv preprint arXiv:1906.01787* (2019).

[32] C. Ying, T. Cai, S. Luo, S. Zheng, G. Ke, D. He, Y. Shen & T.-Y. Liu. "Do transformers really perform badly for graph representation?" *Advances in neural information processing systems* **34**, 28877 (2021).

[33] T. Dao, D. Fu, S. Ermon, A. Rudra & C. Ré. "FlashAttention: Fast and Memory-Efficient Exact Attention with IO-Awareness." In *Advances in Neural Information Processing Systems* (Curran Associates, Inc., 2022). Ed. by S. Koyejo, S. Mohamed, A. Agarwal, D. Belgrave, K. Cho & A. Oh. Vol. 35, 16344–16359.

[34] The JAX Team. *Pallas: a JAX kernel language.* 2023. URL: https://docs.jax.dev/en/latest/pallas/index.html.

[35] J. Gilmer, S. S. Schoenholz, P. F. Riley, O. Vinyals & G. E. Dahl. "Neural message passing for quantum chemistry." In *International conference on machine learning* (2017). PMLR, 1263–1272.

[36] J. Gilmer, S. S. Schoenholz, P. F. Riley, O. Vinyals & G. E. Dahl. "Message passing neural networks." *Machine learning meets quantum physics*, 199 (2020).

[37] N. Gao & S. Günnemann. "Ab-initio potential energy surfaces by pairing GNNs with neural wave functions." *arXiv preprint arXiv:2110.05064* (2021).

[38] M. Scherbela, L. Gerard & P. Grohs. "Variational Monte Carlo on a Budget—Fine-tuning pre-trained Neural Wavefunctions." *Advances in Neural Information Processing Systems* **36**, 23902 (2023).
[39] N. Metropolis, A. W. Rosenbluth, M. N. Rosenbluth, A. H. Teller & E. Teller. "Equation of state calculations by fast computing machines." *The Journal of Chemical Physics* **21**, 1087 (1953).
[40] U. Grenander & M. I. Miller. "Representations of knowledge in complex systems." *Journal of the Royal Statistical Society: Series B (Methodological)* **56**, 549 (1994).
[41] Y. Song & S. Ermon. "Generative Modeling by Estimating Gradients of the Data Distribution." In *Advances in Neural Information Processing Systems* (Curran Associates, Inc., 2019). Ed. by H. Wallach, H. Larochelle, A. Beygelzimer, F. d'Alché-Buc, E. Fox & R. Garnett. Vol. 32.
[42] G. O. Roberts & R. L. Tweedie. "Exponential convergence of Langevin distributions and their discrete approximations." *Bernoulli* (1996).
[43] G. O. Roberts & J. S. Rosenthal. "Optimal scaling of discrete approximations to Langevin diffusions." *Journal of the Royal Statistical Society: Series B (Statistical Methodology)* **60**, 255 (1998).
[44] R. M. Neal. "Annealed importance sampling." *Statistics and computing* **11**, 125 (2001).
[45] M. Welling & Y. W. Teh. "Bayesian learning via stochastic gradient Langevin dynamics." In *Proceedings of the 28th international conference on machine learning (ICML-11)* (2011). Citeseer, 681–688.
[46] J. Martens & R. Grosse. "Optimizing neural networks with kronecker-factored approximate curvature." In *International conference on machine learning* (2015). PMLR, 2408–2417.
[47] A. Botev & J. Martens. *KFAC-JAX*. Version 0.0.6. 2022. URL: https://github.com/google-deepmind/kfac-jax.
[48] N. Gao, J. Köhler, A. Foster & P. B. Szabó. *folx - Forward Laplacian for JAX*. Version 0.2.13. 2023. URL: http://github.com/microsoft/folx.
[49] Y. Bengio, J. Louradour, R. Collobert & J. Weston. "Curriculum learning." In *Proceedings of the 26th annual international conference on machine learning* (2009), 41–48.
[50] J. Clayden, N. Greeves & S. Warren. *Organic chemistry* (Oxford university press, 2012).
[51] U. Wietelmann & J. Klett. "200 years of lithium and 100 years of organolithium chemistry." *Zeitschrift für anorganische und allgemeine Chemie* **644**, 194 (2018).
[52] R. J. Grams, W. L. Santos, I. R. Scorei, A. Abad-García, C. A. Rosenblum, A. Bita, H. Cerecetto, C. Viñas & M. A. Soriano-Ursúa. "The rise of boron-containing compounds: advancements in synthesis, medicinal chemistry, and emerging pharmacology." *Chemical reviews* **124**, 2441 (2024).
[53] W. Jiang, N. J. DeYonker & A. K. Wilson. "Multireference character for 3d transition-metal-containing molecules." *Journal of chemical theory and computation* **8**, 460 (2012).
[54] T. J. Lee & G. E. Scuseria. "Achieving chemical accuracy with coupled-cluster theory." In *Quantum Mechanical Electronic Structure Calculations with Chemical Accuracy* (Springer, 1995), 47–108.
[55] J. Parsons, J. B. Holmes, J. M. Rojas, J. Tsai & C. E. Strauss. "Practical conversion from torsion space to Cartesian space for in silico protein synthesis." *Journal of computational chemistry* **26**, 1063 (2005).
[56] W. R. Gilks & P. Wild. "Adaptive rejection sampling for Gibbs sampling." *Journal of the Royal Statistical Society: Series C (Applied Statistics)* **41**, 337 (1992).
[57] P. Kharya. *TensorFloat-32 in the A100 GPU Accelerates AI Training, HPC up to 20x*. 2020. URL: https://blogs.nvidia.com/blog/tensorfloat-32-precision-format/.
[58] P. J. Huber. "Robust estimation of a location parameter." In *Breakthroughs in statistics: Methodology and distribution* (Springer, 1992), 492–518.
[59] Q. Sun, T. C. Berkelbach, N. S. Blunt, G. H. Booth, S. Guo, Z. Li, J. Liu, J. D. McClain, E. R. Sayfutyarova, S. Sharma, et al. "PySCF: the Python-based simulations of chemistry framework." *Wiley Interdiscip. Rev. Comput. Mol. Sci.* **8**, e1340 (2018). DOI: https://doi.org/10.1002/wcms.1340.
[60] V. K. Prasad, M. H. Khalilian, A. Otero-de-la-Roza & G. A. DiLabio. "BSE49, a diverse, high-quality benchmark dataset of separation energies of chemical bonds." *Scientific data* **8**, 300 (2021).
[61] H. Jónsson, G. Mills & K. W. Jacobsen. "Nudged elastic band method for finding minimum energy paths of transitions." In *Classical and quantum dynamics in condensed phase simulations* (World Scientific, 1998), 385–404.
[62] G. Henkelman, B. P. Uberuaga & H. Jónsson. "A climbing image nudged elastic band method for finding saddle points and minimum energy paths." *The Journal of chemical physics* **113**, 9901 (2000).
[63] E. L. Kolsbjerg, M. N. Groves & B. Hammer. "An automated nudged elastic band method." *The Journal of Chemical Physics* **145** (2016).
[64] A. H. Larsen, J. J. Mortensen, J. Blomqvist, I. E. Castelli, R. Christensen, M. Dułak, J. Friis, M. N. Groves, B. Hammer, C. Hargus, et al. "The atomic simulation environment—a Python library for working with atoms." *Journal of Physics: Condensed Matter* **29**, 273002 (2017).
[65] J. P. Perdew, K. Burke & M. Ernzerhof. "Generalized gradient approximation made simple." *Physical review letters* **77**, 3865 (1996).
[66] F. Weigend & R. Ahlrichs. "Balanced basis sets of split valence, triple zeta valence and quadruple zeta valence quality for H to Rn: Design and assessment of accuracy." *Phys. Chem. Chem. Phys.* **7**, 3297 (2005). DOI: 10.1039/b508541a.
[67] S. Grimme, J. Antony, S. Ehrlich & H. Krieg. "A consistent and accurate ab initio parametrization of density functional dispersion correction (DFT-D) for the 94 elements H-Pu." *The Journal of Chemical Physics* **132** (2010).
[68] S. Grimme, S. Ehrlich & L. Goerigk. "Effect of the damping function in dispersion corrected density functional theory." *Journal of Computational Chemistry* **32**, 1456 (2011).
[69] F. Neese. "Software update: the ORCA program system, version 5.0." *WIRES Comput. Molec. Sci.* **12**, e1606 (2022). DOI: 10.1002/wcms.1606.
[70] S. Smidstrup, A. Pedersen, K. Stokbro & H. Jónsson. "Improved initial guess for minimum energy path calculations." *The Journal of chemical physics* **140** (2014).
[71] E. Bitzek, P. Koskinen, F. Gähler, M. Moseler & P. Gumbsch. "Structural relaxation made simple." *Physical review letters* **97**, 170201 (2006).
[72] H. Lischka, E. Ventura & M. Dallos. "The Diels–Alder Reaction of Ethene and 1,3-Butadiene: An Extended Multireference ab initio Investigation." *ChemPhysChem* **5**, 1365 (2004). DOI: 10.1002/cphc.200400104.
[73] C.-X. Cui & Y.-J. Liu. "A thorough understanding of the Diels—Alder reaction of 1,3-butadiene and ethylene." *Journal of Physical Organic Chemistry* **27**, 652 (2014). DOI: 10.1002/poc.3313.
[74] M. Uchiyama, T. Tomioka & A. Amano. "Thermal decomposition of cyclohexene." *The Journal of Physical Chemistry* **68**, 1878 (1964).

[75] R. J. Le Roy, Y. Huang & C. Jary. "An accurate analytic potential function for ground-state N2 from a direct-potential-fit analysis of spectroscopic data." *The Journal of chemical physics* **125** (2006).

[76] R. J. Gdanitz. "Accurately solving the electronic Schrödinger equation of atoms and molecules using explicitly correlated (r12-)MR-CI: the ground state potential energy curve of N2." *Chemical Physics Letters* **283**, 253 (1998). DOI: https://doi.org/10.1016/S0009-2614(97)01392-4.

[77] M. S. Mirzaei, A. A. Taherpour & C. Wentrup. "Azulene–Naphthalene, Naphthalene–Naphthalene, and Azulene–Azulene Rearrangements." *The Journal of Organic Chemistry* **87**, 11503 (2022). DOI: 10.1021/acs.joc.2c01099.

[78] J. M. L. Martin & G. de Oliveira. "Towards standard methods for benchmark quality ab initio thermochemistry—W1 and W2 theory." *The Journal of Chemical Physics* **111**, 1843 (1999). DOI: 10.1063/1.479454.

[79] B. Ruscic, R. E. Pinzon, G. v. Laszewski, D. Kodeboyina, A. Burcat, D. Leahy, D. Montoy & A. F. Wagner. "Active Thermochemical Tables: thermochemistry for the 21st century." *Journal of Physics: Conference Series* **16**, 561 (2005). DOI: 10.1088/1742-6596/16/1/078.

[80] A. Karton, E. Rabinovich, J. M. L. Martin & B. Ruscic. "W4 theory for computational thermochemistry: In pursuit of confident sub-kJ/mol predictions." *The Journal of Chemical Physics* **125**, 144108 (2006). DOI: 10.1063/1.2348881.

[81] F. M. Bickelhaupt & E. J. Baerends. "Kohn-Sham density functional theory: predicting and understanding chemistry." *Reviews in computational chemistry*, 1 (2000).

[82] C. Roothaan. "Self-consistent field theory for open shells of electronic systems." *Reviews of modern physics* **32**, 179 (1960).

[83] Q. Sun, J. Yang & G. K.-L. Chan. "A general second order complete active space self-consistent-field solver for large-scale systems." *Chemical Physics Letters* **683**, 291 (2017).

[84] P. J. Knowles, C. Hampel & H.-J. Werner. "Coupled Cluster Theory for High Spin Open Shell Reference Wavefunctions." *J. Chem. Phys.* **99**, 5219 (1993). DOI: 10.1063/1.465990.

[85] C. Angeli, R. Cimiraglia, S. Evangelisti, T. Leininger & J.-P. Malrieu. "Introduction of n-electron valence states for multireference perturbation theory." *The Journal of Chemical Physics* **114**, 10252 (2001).

[86] P. G. Szalay, T. Muller, G. Gidofalvi, H. Lischka & R. Shepard. "Multiconfiguration self-consistent field and multireference configuration interaction methods and applications." *Chemical reviews* **112**, 108 (2012).

[87] R. A. Kendall, T. H. Dunning & R. J. Harrison. "Electron Affinities of the First-row Atoms Revisited. Systematic Basis Sets and Wave Functions." *J. Chem. Phys.* **96**, 6796 (1992). DOI: 10.1063/1.462569.

[88] F. Neese & E. F. Valeev. "Revisiting the atomic natural orbital approach for basis sets: Robust systematic basis sets for explicitly correlated and conventional correlated ab initio methods?" *Journal of chemical theory and computation* **7**, 33 (2011).

[89] A. D. Becke. "Density-functional thermochemistry. III. The role of exact exchange." *The Journal of Chemical Physics* **98**, 5648 (1993). DOI: 10.1063/1.464913.

[90] S. H. Vosko, L. Wilk & M. Nusair. "Accurate spin-dependent electron liquid correlation energies for local spin density calculations: a critical analysis." *Canadian Journal of physics* **58**, 1200 (1980).

[91] C. Lee, W. Yang & R. G. Parr. "Development of the Colle-Salvetti correlation-energy formula into a functional of the electron density." *Physical review B* **37**, 785 (1988).

[92] P. J. Stephens, F. J. Devlin, C. F. Chabalowski & M. J. Frisch. "Ab initio calculation of vibrational absorption and circular dichroism spectra using density functional force fields." *The Journal of physical chemistry* **98**, 11623 (1994).

[93] F. Weigend, F. Furche & R. Ahlrichs. "Gaussian basis sets of quadruple zeta valence quality for atoms H–Kr." *The Journal of chemical physics* **119**, 12753 (2003).

[94] S. Ehlert. "Simple DFT-D3: Library first implementation of the D3 dispersion correction." *Journal of Open Source Software* **9**, 7169 (2024).

[95] Y. Zhao & D. G. Truhlar. "The M06 suite of density functionals for main group thermochemistry, thermochemical kinetics, noncovalent interactions, excited states, and transition elements: two new functionals and systematic testing of four M06-class functionals and 12 other functionals." *Theoretical chemistry accounts* **120**, 215 (2008).

[96] M. Cafiero. "Role of exact exchange and empirical dispersion in density functional theory-based three-body noncovalent interactions." *The Journal of Physical Chemistry A* **128**, 8777 (2024).

[97] M. Cafiero. "Role of exact exchange and empirical dispersion in density functional theory-based three-body noncovalent interactions." *The Journal of Physical Chemistry A* **128**, 8777 (2024).

[98] J.-D. Chai & M. Head-Gordon. "Long-range corrected hybrid density functionals with damped atom–atom dispersion corrections." *Physical Chemistry Chemical Physics* **10**, 6615 (2008).

[99] N. Mardirossian & M. Head-Gordon. "ωB97X-V: A 10-parameter, range-separated hybrid, generalized gradient approximation density functional with nonlocal correlation, designed by a survival-of-the-fittest strategy." *Physical Chemistry Chemical Physics* **16**, 9904 (2014).

[100] J. D. Watts, J. Gauss & R. J. Bartlett. "Coupled-cluster methods with noniterative triple excitations for restricted open-shell Hartree–Fock and other general single determinant reference functions. Energies and analytical gradients." *The Journal of chemical physics* **98**, 8718 (1993).

[101] C. Angeli, R. Cimiraglia & J.-P. Malrieu. "N-electron valence state perturbation theory: a fast implementation of the strongly contracted variant." *Chemical physics letters* **350**, 297 (2001).

[102] C. Angeli, R. Cimiraglia & J.-P. Malrieu. "n-electron valence state perturbation theory: A spinless formulation and an efficient implementation of the strongly contracted and of the partially contracted variants." *The Journal of chemical physics* **117**, 9138 (2002).

[103] P. J. Knowles & N. C. Handy. "A new determinant-based full configuration interaction method." *Chemical Physics Letters* **111**, 315 (1984).

[104] J. Pipek & P. G. Mezey. "A fast intrinsic localization procedure applicable for ab initio and semiempirical linear combination of atomic orbital wave functions." *The Journal of Chemical Physics* **90**, 4916 (1989).

[105] G. L. Stoychev, A. A. Auer & F. Neese. "Automatic generation of auxiliary basis sets." *Journal of chemical theory and computation* **13**, 554 (2017).
