# Supplementary material for: An ab initio foundation model of wavefunctions that accurately describes chemical bond breaking
Source: Nat Commun. 2026 Aug 21;17:9976. doi: 10.1038/s41467-026-76604-2 (PMC13590640; doi:10.1038/s41467-026-76604-2)
Supplement: Supplementary file 2 [file 41467_2026_76604_MOESM2_ESM.pdf]

nature portfolio

Peer Review File

An ab initio foundation model of wavefunctions that accurately describes chemical bond breaking

Corresponding Author: Dr Adam Foster

This file contains all reviewer reports in order by version, followed by all author rebuttals in order by version.

Version 0:

Reviewer comments:

Reviewer #1

(Remarks to the Author)
The authors present an attempt to generalize machine-learning many-body wave functions to be general across multiple chemical systems. They study systems smaller than 48 electrons (mostly less than 25 electrons it seems) and that are limited to contain only of H, Li, F, C, Li, N, F, O. I think it's a nice work that represents progress in the field, and leaves more work to be done, since as presented the architecture is fairly limited, in that it has a maximum number of atoms/electrons that it can treat.

I support publication of this work, although I have a number of concerns with it that I think can be fixed with another revision:

MEP appears to never be defined. Minimum energy pathway? Not sure.

Fig 3g-i are hopelessly convoluted. Why do they have apparently random xticks? The top xticks overlap with the bottom xticks in such a way that it is almost unreadable. If they are converting between GPU and CPU time in some way, just plot the single universal quantity on the y-axis. If they are performing a log-log plot then mark the powers of 10 with subticks in between, not random numbers with 5 digits of precision. Similarly, it is almost impossible to read anything useful from these plots, with there being multiple circles with almost the same color, and the other methods scattered around. They reference a Pareto front but do not tell us on the graph where they think that it is.

Can they explain why they needed a very small step size to obtain a reasonable acceptance rate? There are algorithms that have been around for a long time (see for example Umrigar, Nightingale, and Runge) that fix many of the problems they seem to be having and would not require complicated procedures. Step sizes of approximately 1 Hartree^-1 are typical for systems like the ones they are studying.

Mean absolute relative error is also not clearly defined. What formula is actually being used here?

There are a few over-the-top statements like "In totality, these cost reductions bring highly accurate deep QMC to the Pareto frontier of classical methods for the first time."

They did not test all classical methods. For example, diffusion Monte Carlo on a single determinant often gets high accuracy for fairly low costs for systems like that. Maybe it's not as high accuracy as the NN wave function but it seems odd to omit it. Also they only tested UCCSD(T)/CBS, which is quite expensive [and did they define it?]. For relative energies, CCSD(T) with a finite basis is often pretty accurate but this is not shown. The work is sufficient for saying that their method is worth a look and an impressive advance but they have not shown that it's better than all possible classical methods.

pg 7 line 184: What does "we fine-tune transferably" mean?

Some of the formatting is odd, with solitary sentences instead of paragraphs.

--- In the

"Perhaps the most straightforward approach to constructing a size consistent ansatz is to employ a single Slater-determinant built from orbitals localized on either one of the two subsystems, with the correct number of orbitals assigned to each. Then, if the subsystems are sufficiently distant, the determinant will factorize into the above-mentioned tensor product form."

A Slater determinant is invariant to unitary transformations. I understand what they are trying to say, but at the end of the day their wave function is not size consistent so I'm not sure what the point of this discussion is.

(Remarks on code availability)
I looked at the repository and it appears they have provided a nice set of documentation and the code is public. I didn't review the code beyond that.

Reviewer #2

(Remarks to the Author)
The manuscript presents an approach to perform Quantum Monte Carlo (QMC) using deep neural networks. The main novelty is considering also the position of the ions as input of the network, and not as parameters. This strategy, is similar to the one used in ref 29 for QMC on lattice models allows improving the transferability of the ansatz on the molecular orbitals between different molecules. This, in turn, allows improving the convergence speed with respect to other NN-based QMC approaches (for example ref 26 and 27). The results are of a good quality, and (in my opinion)m deserve publication) but I have some concerns and questions:

1) (CRITICAL) Validations agains experimental results is partially performed (in particular in Fig 2), but it is not sufficiently convincing for a high-level paper on this topic. Experimental dissociation and association free energies in the gas phase and kinetic measurements are available for several small molecules. Using QMC as a reference, as done in fig 3 and elsewhere, appears somewhat self-referential given that this same method was used to generate to train the network. In any case, the term "chemical accuracy" should be used with care, since QMC itself uses critical assumptions to optimise a fermionic many-electron wave function, and, to my knowledge, no guarantee exists that its variational ansatz is correct in general. I think it would be appropriate searching carefully the literature for databases with experimental measurements in the gas phase and perform large scale validation against those.

2) Is it possible to determine whether a molecule lies outside the training distribution (i.e., is off-sample)? Does the approach offer an internal reliability or uncertainty estimation metric for this purpose?

3) The term “foundational” appears in the title and throughout the introduction, yet the text repeatedly states that the approach requires fine-tuning for each specific task. Could you please clarify the intended meaning of “foundational” in this context? In my opinion the approach introduces a very smart manner for initialising NN-QMC calculations, but does not offer the genuine the zero-shot functionalities which one would would expect in a foundational model.

4) (CRITICAL) At the end of page 2, it is stated that everything is fine-tuned to work with localised orbitals. In this regard, can the approach adequately describe aromaticity and large-scale conjugation (for example the Azulene to Naftalene transition)? Achieving chemical accuracy in QMC simulations for large-scale conjugated systems remains, in my view, one of the major open challenges in computational quantum chemistry. It is not clear from the manuscript whether the proposed approach provides a pathway to address this issue. If not, the scope of the work is in my opinion more limited than claimed in the abstract and in the introduction. This limitation, if present, should be at least explicitly declared in the abstract and discussed in depth.

(Remarks on code availability)

Version 1:

Reviewer comments:

Reviewer #1

(Remarks to the Author)
The authors have addressed my concerns to my satisfaction.

The one small thing I would say is that it does not appear that Fig 3a-f are labeled. They say what the black lines are but not what the brown ones are from what I can tell.

(Remarks on code availability)

Reviewer #2

(Remarks to the Author)
I thank the authors for having carefully taken into account my concerns. I am happy about the test performed on azulene-nafalene, and of the explanation of the manner in which the approach takes into account of orbital delocalisation. I am also happy of the new comparison of Orbformer energies to experimental atomization energies.
I am still slightly confused by the statements in paragraph Validation of the QMC reference protocol for Figure 3, which I find not very convincing. I suggest adding this paragraph in the text, in such a manner that the readers will be able to judge how QMC was precisely used as a benchmark.
I also doubt that a generic reader would be able to appreciate the difference between "foundational model"--all purpose and zero shot in the common perception and "foundation model"--ready for fine tuning. My suggestion would be to avoid using this term in the title, and explaining where it first appears what it means.

(Remarks on code availability)

Open Access This Peer Review File is licensed under a Creative Commons Attribution 4.0 International License, which permits use, sharing, adaptation, distribution and reproduction in any medium or format, as long as you give appropriate credit to the original author(s) and the source, provide a link to the Creative Commons license, and indicate if changes were made.
In cases where reviewers are anonymous, credit should be given to 'Anonymous Referee' and the source.
The images or other third party material in this Peer Review File are included in the article’s Creative Commons license, unless indicated otherwise in a credit line to the material. If material is not included in the article’s Creative Commons license and your intended use is not permitted by statutory regulation or exceeds the permitted use, you will need to obtain permission directly from the copyright holder.
To view a copy of this license, visit https://creativecommons.org/licenses/by/4.0/

Response to Reviewers

We thank the reviewers for investing their time to review our manuscript and we appreciate the thoughtful comments. Below, we provide a point-by-point response to the separate reviews. Here, we point out some important points for the updated version of our paper:

- We have added a new computational experiment on the azulene–naphthalene isomerization (Appendix K.5). We showed that fine-tuning Orbformer can achieve excellent agreement with CCSD(T)/CBS on the overall reaction energy. This provides firm evidence that Orbformer can handle conjugated systems well.
- We added new calculations benchmarking Orbformer on a range of experimental atomization energies from ATcT [Ruscic et al., 2005], finding across the board good agreement between our predictions and the experimental values (Appendix K.6). This provides stronger confirmation that Orbformer, like other deep QMC methods, has the capacity to reach excellent agreement with experiment.
- We would draw reviewers' attention to the existing experiment presented in Section K.2, in which Orbformer was compared to an experimental dissociation curve for the nitrogen dimer. Here, Orbformer was shown to agree every well with experiment with errors that are within the reported tolerance of the experiment itself. In a new comparison, the divergence from the experiment is shown to be consistent with a non-QMC computational method.
- Figure 3 (g)–(k) has been remade, with no changes to the underlying data.

Reviewer #1

> The authors present an attempt to generalize machine-learning many-body wave functions to be general across multiple chemical systems. They study systems smaller than 48 electrons (mostly less than 25 electrons it seems) and that are limited to contain only of H, Li, F, C, Li, N, F, O. I think it's a nice work that represents progress in the field, and leaves more work to be done, since as presented the architecture is fairly limited, in that it has a maximum number of atoms/electrons that it can treat.

We would like to emphasize that our model does *not* have a maximum number of electrons/atoms that it can treat, nor did we limit the experiments presented to 48 electrons. First, the largest system that we applied our model to in experiments in the paper was the $C_{13}H_{28}$ alkane with 106 electrons (see Sec. K.4 for full details on the alkanes experiment). This was mentioned in Sections 2.5 and 4.5, but we have now mentioned this achievement more prominently in Section 2.1. Second, it is correct that a maximum number of electrons/atoms

is set *for a specific JAX compilation of the model* (see Sec. C.1). However, no parameter of our model depends on these maximum sizes and the same parameters can be recompiled into a model that handles larger systems. Indeed, this is exactly how we trained our model—first compiling and training up to 10 electrons, then recompiling and continuing training at 24 electrons, before taking the same parameters, recompiling again, and fine-tuning on a variety of system sizes up to 106 electrons. We agree that this was not clearly explained and have updated the text to draw attention to this key strong point of our method (updated Sec. 2.1 from line 99).

> I support publication of this work, although I have a number of concerns with it that I think can be fixed with another revision:

We are very pleased to hear that you are supportive of publication of this work.

> MEP appears to never be defined. Minimum energy pathway? Not sure.

Yes, MEP means minimum energy pathway. This has been corrected in the text (line 151).

> Fig 3g-i are hopelessly convoluted. Why do they have apparently random xticks? The top xticks overlap with the bottom xticks in such a way that it is almost unreadable. If they are converting between GPU and CPU time in some way, just plot the single universal quantity on the y-axis. If they are performing a log-log plot then mark the powers of 10 with subticks in between, not random numbers with 5 digits of precision. Similarly, it is almost impossible to read anything useful from these plots, with there being multiple circles with almost the same color, and the other methods scattered around. They reference a Pareto front but do not tell us on the graph where they think that it is.

You are correct that the multiple x-axes show a conversion between CPU and GPU hours as described in the caption. We have updated this to now show only GPU hours and with more regular x labels, CPU hours are implicitly converted. We have also updated the colour convention to be simpler: all non-QMC baselines use the same orange colour with marker symbols to pick out specific methods. The Pareto fronts refer here to all plots (g)-(k) which are all cost against error plots for different systems. We have explained this more carefully in the supporting text (updated Sec. 2.3 from line 143). We believe that with these changes the presentation of the figure is now clearer.

> Can they explain why they needed a very small step size to obtain a reasonable acceptance rate? There are algorithms that have been around for a long time (see for example Umrigar, Nightingale, and Runge) that fix many of the problems they seem to be having and would not require complicated procedures. Step sizes of approximately 1 Hartree^{-1} are typical for systems like the ones they are studying.

We first emphasize that our discussion of sampling methods focused on the problem of re-equilibrating for transferable training, i.e. if the same ansatz is to be applied to many different molecules, and a completely different molecule will be seen at each gradient step. We do not believe that earlier authors focused on this specific problem. Indeed, existing sampling approaches are completely suitable for us when we train on one or a few molecules, because the samplers need only be equilibrated at the start of training, rather than continually re-equilibrated throughout training. All fine-tuning on single or small numbers of molecules used existing sampling procedures. To specifically answer your question, when running MALA, the step size is set automatically to reach a target acceptance rate of 57%, which is inline with best practices for Langevin Monte Carlo. Numerous proposal schemes exist, which likely accounts for specific numerical difference between your quoted step size and our plot. Our proposal scheme updates all electron positions simultaneously, whereas updating only a few electrons at a time admits a larger step size but needs more total steps. Finally, we argue that our proposed approach for faster burn-in using ULA is not particularly complicated, if anything it is slightly simpler than MALA itself.

> Mean absolute relative error is also not clearly defined. What formula is actually being used here?

Suppose we have a reaction curve with geometries indexed by $i = 1, \dots, n$. Suppose that we have corresponding energy curves $(E_i)_{i=1}^n$ and $(E'_i)_{i=1}^n$. Let $\bar{E} = \frac{1}{n}\sum_{i=1}^n E_i$ and $\bar{E}' = \frac{1}{n}\sum_{i=1}^n E'_i$ Then the mean absolute relative error is defined by

$$\mathrm{MARE}(E_1, \dots, E_n; E'_1, \dots, E'_n) = \frac{1}{n}\sum_{i=1}^n |(E_i - \bar{E}) - (E'_i - \bar{E}')| \qquad (1)$$

We have now included this definition in the manuscript for clarity in Section H.3.

> There are a few over-the-top statements like ”In totality, these cost reductions bring highly accurate deep QMC to the Pareto frontier of classical methods for the first time.”

> They did not test all classical methods. For example, diffusion Monte Carlo on a single determinant often gets high accuracy for fairly low costs for systems like that. Maybe it’s not as high accuracy as the NN wave function but it seems odd to omit it. Also they only tested UCCSD(T)/CBS, which is quite expensive [and did they define it?]. For relative energies, CCSD(T) with a finite basis is often pretty accurate but this is not shown. The work is sufficient for saying that their method is worth a look and an impressive advance but they have not shown that it’s better than all possible classical methods.

We fully accept that our statement only applies to the classical methods and settings that we studied in this paper, and we have updated the statement

accordingly (line 163). The exact UCCSD(T)/CBS protocol that was used is described in Appendix L.

> pg 7 line 184: What does "we fine-tune transferably" mean?

'Transferable' or 'joint' fine-tuning refers to the approach described at the end of Section 2.1: "We do not resort to separate fine-tuning for every structure; instead, we group target structures together and fine-tune them using a single set of model parameters. For example, we routinely fine-tune all geometries with the same set of nuclei together." The key point is that a single set of parameters is used for ∼20 molecules all the way down to convergence. We also updated the appropriate sentence on page 7 to be clearer (now line 190).

> Some of the formatting is odd, with solitary sentences instead of paragraphs.

We have sought to correct any single sentence paragraphs in the latest revision.

> In the "Perhaps the most straightforward approach to constructing a size consistent ansatz is to employ a single Slater-determinant built from orbitals localized on either one of the two subsystems, with the correct number of orbitals assigned to each. Then, if the subsystems are sufficiently distant, the determinant will factorize into the above-mentioned tensor product form." A Slater determinant is invariant to unitary transformations. I understand what they are trying to say, but at the end of the day their wave function is not size consistent so I'm not sure what the point of this discussion is.

We emphasize that the Orbformer wavefunction *is* size consistent with a single determinant. The point of this discussion is to motivate certain design choices in our model. In Section C.1, we discuss at length the principle of 'Composability', which builds on this earlier discussion about size consistency. We found that this principle was important in achieving generalization between different molecules, particularly molecules of different sizes. Surprisingly, this holds even for > 1 determinant. We would be happy to clarify further if you have any specific questions on this topic.

> I looked at the repository and it appears they have provided a nice set of documentation and the code is public. I didn't review the code beyond that.

We appreciate your taking the time to look at the public repository.

Reviewer #2

> The manuscript presents an approach to perform Quantum Monte Carlo (QMC) using deep neural networks. The main novelty is considering also the position of the ions as input of the network, and not as parameters. This strategy, is similar to the one used in ref 29 for QMC on lattice models allows improving the transferability of the ansatz on the molecular orbitals

> between different molecules. This, in turn, allows improving the convergence speed with respect to other NN-based QMC approaches (for example ref 26 and 27). The results are of a good quality, and (in my opinion)m deserve publication) but I have some concerns and questions:

We thank the reviewer for this positive overall assessment of the work.

> (CRITICAL) Validations against experimental results is partially performed (in particular in Fig 2), but it is not sufficiently convincing for a high-level paper on this topic. Experimental dissociation and association free energies in the gas phase and kinetic measurements are available for several small molecules. Using QMC as a reference, as done in fig 3 and elsewhere, appears somewhat self-referential given that this same method was used to generate to train the network. In any case, the term "chemical accuracy" should be used with care, since QMC itself uses critical assumptions to optimise a fermionic many-electron wave function, and, to my knowledge, no guarantee exists that its variational ansatz is correct in general. I think it would be appropriate searching carefully the literature for databases with experimental measurements in the gas phase and perform large scale validation against those.

Motivation for Figure 3. In Fig. 3 we explore the capability of Orbformer *in strongly correlated regimes*. It is exactly these regimes where existing methods most often break down, and so is the most practical use case for a new quantum chemistry method. Obtaining experimental references for the strongly correlated regime is difficult—dissociation energies, for example, compare the bound and dissociated state which are both typically single reference. Coupled cluster algorithms can already handle these types of problem. The Diels–Alder activation energy was one of the few exceptions because it involves the transition state which is multi-reference. To really show the potential of Orbformer in the strongly correlated regime, we required a reference method of sufficient accuracy covering the 20-point reaction curves shown in Fig 3. (a)–(c).

Validation of the QMC reference protocol for Figure 3. After a process of elimination of other insufficiently accurate methods, we settled on using deep QMC as the reference for Figure 3. We then took several steps to ensure and validate the reliability of our reference energies. First, we used a different wavefunction ansatz, Psiformer, for as many of the reference energies as possible. This reduces concerns about using the same ansatz twice. When Psiformer became unstable to train, we switched to using Orbformer without any transferability and without using the pretrained checkpoint. Then, in Section I.2, we compute the level of Monte Carlo error (a transform of the local energy variance), a good indication of the level of error of QMC calculation (Table 7). We continued training of the reference wavefunctions until the error here was acceptably low. Further, in Table 8, we ***compare the end-point energies with highly accurate (RO)CBS-QB3***. These endpoints represent the

single-reference components of the curve where traditional methods are still expected to be highly accurate. For the endpoints, we found excellent agreement between our QMC calculations and (RO)CBS-QB3, providing another level of confidence in these reference energies.

Existing experimental comparison for dinitrogen dissociation. We would also like to draw your attention to Section K.2, in which we presented an experiment comparing Orbformer and other QMC predictions to an *experimental* dissociation curve for the nitrogen dimer. This is the only case we are aware of in which an experimental reference for the interior of a bond-breaking curve is given, and Orbformer matches well with the experiment. Nevertheless, the original experimental paper from which this reference is obtained [Le Roy et al., 2006] contains numerous caveats about the accuracy of the experimental reference in the interior region (i.e. away from the bound and unbound endpoints). Our previous write-up of this experiment focused on the ability to deal with 'distractions', i.e. Orbformer achieves essentially the same N_2 results when ethene is also included in the joint fine-tuning set. We have now brought up the equally important point that it provides an experimental benchmark (updated Sec. K.2 and pointer in the main text on line 127). We also added a comparison with r12-MR-ACPF [Gdanitz, 1998], which closely aligns with ours and previous QMC computations, indicating that divergences from the reported experimental dissociation are not artifacts of the QMC approximation.

References for other experiments. No other experiment uses QMC as a reference—Fig. 2 is experimental, Fig. 4 is CCSD(T), Fig. 14 is experimental, Fig. 17 is CCSD(T) and Fig. 19 is experimental.

Prior evidence for deep QMC as an accurate but expensive method. We also mention that several previous deep QMC papers have conducted comparison with experiment. The consensus of the community appears to be that deep QMC is a reliable method that is primarily limited by computation cost, not expressivity of the ansatz [von Glehn et al., 2022, Scherbela et al., 2025].

New experimental evidence comparing Orbformer to experimental atomization energies. Following your comments we have performed an additional experiment by comparing Orbformer's total atomization energies with experimentally determined values for 19 small molecules. The experiment is described in the new Section K.6 of the Supporting Information which is referenced in Section 2.2 of the main text (updated line 128).

> Is it possible to determine whether a molecule lies outside the training distribution (i.e., is off-sample)?

When it comes to practical out-of-distribution decisions for the pretrained checkpoint that we ship with the model, we believe Figure 4 is the most useful guidance here—it shows that changing the chemical composition of a molecule

by introducing a completely unseen atom type will negate almost all the benefits of pretraining, whilst changing the number of specific atoms types and their geometry retains some substantial benefit from pretraining in every case that we explored (Fig. 3 also matches this latter case).

> Does the approach offer an internal reliability or uncertainty estimation metric for this purpose?

We repeatedly found that *the variance of the local energy* is by far the most reliable and repeatable measure of uncertainty. At exact convergence to the ground state, the variance of the local energy is exactly zero. Several recent papers have proposed power laws that relate the local energy variance to anticipated energy error [Fu et al., 2024]. Without advocating for a very specific formula, we reliably find that the order of magnitude of the local energy variance is highly predictive of energy error.

> The term "foundational" appears in the title and throughout the introduction, yet the text repeatedly states that the approach requires fine-tuning for each specific task. Could you please clarify the intended meaning of "foundational" in this context? In my opinion the approach introduces a very smart manner for initialising NN-QMC calculations, but does not offer the genuine the zero-shot functionalities which one would would expect in a foundational model.

We use term 'foundation model' in the sense in which it was rather recently (ca 2021) established in the field of machine learning. (Note that the established term that we use is 'foundation model', not 'foundational model'.[1]) That is, a foundation model is a large general machine-learning model that has been trained on a broad range of data and can be later fine-tuned for specific applications.

> (CRITICAL) At the end of page 2, it is stated that everything is fine-tuned to work with localised orbitals. In this regard, can the approach adequately describe aromaticity and large-scale conjugation (for example the Azulene to Naftalene transition)? Achieving chemical accuracy in QMC simulations for large-scale conjugated systems remains, in my view, one of the major open challenges in computational quantum chemistry. It is not clear from the manuscript whether the proposed approach provides a pathway to address this issue. If not, the scope of the work is in my opinion more limited than claimed in the abstract and in the introduction. This limitation, if present, should be at least explicitly declared in the abstract and discussed in depth.

We agree with the reviewer that accurately describing conjugated systems is important. To answer your question directly, we have performed an additional experiment, described in the new Section K.5 of the Supporting Information, where the performance of Orbformer is compared to CCSD(T) at the complete basis set limit on the task of describing the azulene–naphthalene isomerization.

[1] `https://en.wikipedia.org/wiki/Foundation_model`

(A pointer to this section was added in Sec. 2.5 line 200.) We found excellent agreement between the Orbformer reaction energy and the CCSD(T) reference. As part of this experiment, we also present some illustrations of the orbital *envelopes* learned by the model on naphthalene. We can see that relatively delocalised orbitals that span a number of nuclei are learned. This shows the first key point—the degree of localization can be learned and adapted by the model depending on the desired electronic structure. Second, we emphasize that the isosurfaces that we are showing are only the orbital envelopes. As discussed in Section 4.1, the Slater determinants used in our model are a point-wise product between envelopes and transformer representations. Transformer representations, unlike classical orbitals, are functions of all electron positions and spins rather than of one electron's position. The electron–electron attention does decay to zero with distance, but at a slow rate that is not coupled to the envelope localisation. This means that, even with a rather localised envelope, some long-range electron correlation can be captured by the transformer representations. For the specific case of the core orbitals discussed in Section 2.5, we checked that most of the electron–electron communication from the transformer representation is 'switched off', but this is not the case for general Orbformer orbitals.

Finally, we note that benzene was one of our key benchmark systems during Orbformer development and, in combination with this new experiment on azulene/naphthalene, we are confident of the model's ability to describe conjugated systems to high accuracy.

Second Review Round

Reviewer #1

> The one small thing I would say is that it does not appear that Fig 3a-f are labeled. They say what the black lines are but not what the brown ones are from what I can tell.

The caption has been updated to define the black lines, which show the reference energies.

Reviewer #2

> I thank the authors for having carefully taken into account my concerns. I am happy about the test performed on azulene-nafalene, and of the explanation of the manner in which the approach takes into account of orbital delocalisation. I am also happy of the new comparison of Orbformer energies to experimental atomization energies

We are very pleased to hear that the new experimental results were well received.

> I am still slightly confused by the statements in paragraph Validation of the QMC reference protocol for Figure 3, which I find not very convincing. I suggest adding this paragraph in the text, in such a manner that the readers will be able to judge how QMC was precisely used as a benchmark.

We have updated the text of Section 2.3 to be clearer. First, more details about the validation of the reference protocol have been added. We have explicitly stated that any reference to 'accuracy' refers to our chosen reference. Finally, in the closing paragraph of this section we remind the reader that our Orbformer results recover the deep QMC reference energies rather than an experimental reference.

> I also doubt that a generic reader would be able to appreciate the difference between "foundational model"–all purpose and zero shot in the common perception and "foundation model"–ready for fine tuning. My suggestion would be to avoid using this term in the title, and explaining where it first appears what it means.

We fully agree that this term should be directly and clearly defined. We have added a definition and clarification in lines 39-42 of the main Introduction.

References

Weizhong Fu, Weiluo Ren, and Ji Chen. Variance extrapolation method for neural-network variational monte carlo. *Machine Learning: Science and Technology*, 5(1):015016, 2024.

Robert J. Gdanitz. Accurately solving the electronic schrödinger equation of atoms and molecules using explicitly correlated (r12-)MR-CI: the ground state potential energy curve of n2. *Chemical Physics Letters*, 283(5):253–261, 1998. ISSN 0009-2614. doi: https://doi.org/10.1016/S0009-2614(97)01392-4. URL `https://www.sciencedirect.com/science/article/pii/S0009261497013924`.

Robert J Le Roy, Yiye Huang, and Calvin Jary. An accurate analytic potential function for ground-state n2 from a direct-potential-fit analysis of spectroscopic data. *The Journal of chemical physics*, 125(16), 2006.

Branko Ruscic, Reinhardt E. Pinzon, Gregor von Laszewski, Deepti Kodeboyina, Alexander Burcat, David Leahy, David Montoy, and Albert F. Wagner. Active Thermochemical Tables: thermochemistry for the 21st century. *Journal of Physics: Conference Series*, 16(1):561, 2005. ISSN 1742-6596. doi: 10.1088/1742-6596/16/1/078.

Michael Scherbela, Nicholas Gao, Philipp Grohs, and Stephan Günnemann. Accurate Ab-initio Neural-network Solutions to Large-Scale Electronic Structure Problems. *arXiv preprint arXiv:2504.06087*, April 2025. doi: 10.48550/arXiv.

2504.06087. URL http://arxiv.org/abs/2504.06087. arXiv:2504.06087 [physics].

Ingrid von Glehn, James S Spencer, and David Pfau. A self-attention ansatz for ab-initio quantum chemistry. *arXiv preprint arXiv:2211.13672*, 2022.
